# Robust analysis of prokaryotic pangenome gene gain and loss rates with Panstripe

**DOI:** 10.1101/gr.277340.122

**Published:** 2023-01

**Authors:** Gerry Tonkin-Hill, Rebecca A. Gladstone, Anna K. Pöntinen, Sergio Arredondo-Alonso, Stephen D. Bentley, Jukka Corander

**Affiliations:** 1 Department of Biostatistics, University of Oslo, 0372 Blindern, Norway;; 2 Parasites and Microbes, Wellcome Sanger Institute, Cambridge CB10 1RQ, United Kingdom;; 3 Helsinki Institute for Information Technology HIIT, Department of Mathematics and Statistics, University of Helsinki, 00014 Helsinki, Finland

## Abstract

Horizontal gene transfer (HGT) plays a critical role in the evolution and diversification of many microbial species. The resulting dynamics of gene gain and loss can have important implications for the development of antibiotic resistance and the design of vaccine and drug interventions. Methods for the analysis of gene presence/absence patterns typically do not account for errors introduced in the automated annotation and clustering of gene sequences. In particular, methods adapted from ecological studies, including the pangenome gene accumulation curve, can be misleading as they may reflect the underlying diversity in the temporal sampling of genomes rather than a difference in the dynamics of HGT. Here, we introduce Panstripe, a method based on generalized linear regression that is robust to population structure, sampling bias, and errors in the predicted presence/absence of genes. We show using simulations that Panstripe can effectively identify differences in the rate and number of genes involved in HGT events, and illustrate its capability by analyzing several diverse bacterial genome data sets representing major human pathogens.

Genetic variation within microbial populations is shaped by both the accumulation of variation from point mutations as well as by the acquisition and loss of genetic material through horizontal gene transfer (HGT). HGT can occur via the uptake of DNA from the environment, with the help of mobile genetic elements (MGEs; phages, integrative conjugative elements, and plasmids), or from direct contact between bacterial cells (Thomas and Nielsen 2005). Genes are also frequently duplicated and lost vertically upon cell division (Arnold et al. 2022). The influence of these sources of variation varies by species. Clonal species such as *Mycobacterium tuberculosis* (Mtb) typically accumulate variation nearly entirely through point mutations, whereas naturally transformable species such as *Streptococcus pneumoniae* and *Neisseria meningitidis* have very high rates of homologous recombination (Dubnau 1999). In other species such as *Salmonella enterica*, horizontal exchange is generally restricted to the movement of MGEs (Harris et al. 2010). Although HGT does not always have an impact on a microbe's fitness, it can lead to critical phenotypic changes such as the acquisition of antimicrobial resistance, virulence factors, and vaccine escape (Croucher et al. 2013; Wyres et al. 2019).

A common approach to analyzing horizontal exchange in microbial genomics is to group homologous gene sequences into orthologous and paralogous gene clusters. The union of these clusters within a particular species or group is commonly referred to as the pangenome (Medini et al. 2005). Genes are often further classified into either the “core” genome, which is found in almost all members of the group, or the “accessory” genome, which is only found in a subset of genomes. Species with a limited accessory genome such that all genes are likely to have already been observed are often described as “closed,” whereas species with a diverse accessory genome are described as “open.”

A number of tools have been developed to infer a pangenome given a collection of annotated genomes (Page et al. 2015; Ding et al. 2018; Bayliss et al. 2019; Gautreau et al. 2020; Tonkin-Hill et al. 2020; Zhou et al. 2020). A common output of these algorithms is a binary gene presence/absence matrix where genomes are represented by rows and orthologous gene clusters by columns. After generating a gene presence/absence matrix, researchers are often interested in comparing the size of pangenomes between data sets, determining the rate of horizontal gene exchange as well as identifying whether a pangenome is “open” or “closed.”

A gene accumulation curve, as is often performed in ecological studies of species diversity, is often used to investigate these questions (Ugland et al. 2003; Medini et al. 2005). Here, the number of unique gene clusters identified is plotted against the number of genomes. Random permutations are often used to account for the variation caused by the order in which genomes are considered in the plot. In some cases, a power law such as Heaps’ or Zipf's law is fit to this curve to give a parameter estimate of the diminishing number of new genes found with each additional genome and to determine whether the pangenome is open or closed (Tettelin et al. 2008).

A neglected problem with this approach is that it fails to account for the underlying diversity of the set of sampled genomes. For example, a set of genomes taken from within an outbreak is likely to involve far fewer gene exchange events than a diverse sample from a species with thousands of years of evolution separating isolates. Methods that make use of a phylogeny constructed from the genetic diversity present in genes found in all the genomes (the “core” genome) help to address this issue by controlling for the underlying diversity of the sample. The branch lengths of the core genome phylogeny indicate the evolutionary time over which gene gain and loss events could have occurred. Shorter branch lengths separating more closely related taxa would be expected to have fewer associated gene exchange events. Methods that rely on the construction of such a phylogeny include those based on maximum parsimony (Mirkin et al. 2003), maximum likelihood (Hao and Golding 2006; Cohen and Pupko 2010; Han et al. 2013), and Bayesian phylogenetics (Liu et al. 2011). Two notable models that use this approach are the infinitely many genes (IMG) model and the finitely many genes (FMG) model (Baumdicker et al. 2010, 2012; Collins and Higgs 2012; Zamani-Dahaj et al. 2016). The IMG model assumes an infinite pool of genes and that a particular gene can only be gained once, whereas the FMG model assumes that genes belong to a finite pool and that multiple gene gain and loss events of the same gene can occur. Many models also collapse paralogous clusters into gene families before the inference of gene gain and loss rates (Mirkin et al. 2003; Cohen and Pupko 2010; Han et al. 2013).

A significant limitation of these approaches is that they generally assume that there is no error in the inferred pangenome presence/absence matrix. We and others have shown that gene annotation errors and the complexities of clustering genes into orthologous families can introduce substantial numbers of erroneous gene clusters (Han et al. 2013; Salzberg 2019; Tonkin-Hill et al. 2020; Zhou et al. 2020). Although a subset of models do account for errors in the predicted presence/absence of genes, these have mostly been optimized for the analysis of eukaryotes and focus on a small number of gene families involving multiple genes (Han et al. 2013). Most models also make the simplifying assumption that genes are gained or lost individually, which can significantly bias estimates of the rate of gene exchange, particularly when the exchange of MGEs is frequent (Baumdicker et al. 2012; Zamani-Dahaj et al. 2016).

To address these limitations, we have developed Panstripe, an approach that compares the rates of core and accessory genome evolution to account for both population structure and errors in the pangenome gene presence/absence matrix. Using extensive simulations and by analyzing a diverse range of bacterial genome data sets, we show that Panstripe can effectively identify the rate of gene exchange in pangenomes, detect the presence of a temporal signal in the accessory genome, and discern whether the size of gene exchange events varies between pangenomes.

## Results

### Overview

**Figure 1.**
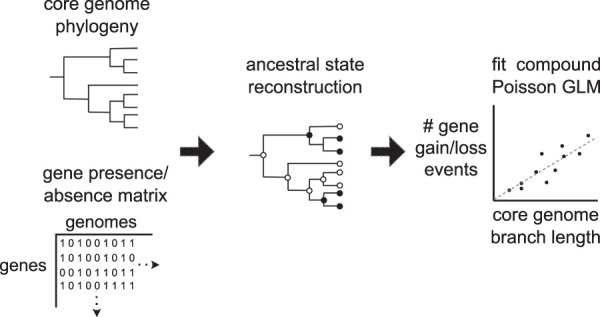
A schematic of the Panstripe algorithm. A binary gene presence/absence matrix and a core genome phylogeny are taken as input. The ancestral states of each gene are then determined before fitting a compound Poisson GLM to compare the core branch length with the number of gene gain and loss events on each branch. Additional terms are included to account for the depth of a branch and whether or not it occurs at the tips of the phylogeny.

Panstripe accepts a phylogeny produced using standard pipelines and a corresponding gene presence/absence matrix as produced by most pangenome inference tools (Page et al. 2015; Lees et al. 2018; Tonkin-Hill et al. 2020). The length of each branch in the phylogeny is then compared with the number of gene gain and loss events inferred to have occurred on that branch using a generalized linear model (GLM) (Fig. 1). The ancestral gene gain and loss events on each branch can be inferred using common ancestral state reconstruction (ASR) methods including maximum parsimony, maximum likelihood, and stochastic mapping (Sankoff 1975; Yang et al. 1995; Cohen and Pupko 2010; Louca and Doebeli 2018). Although this is a critical step in the Panstripe algorithm, we have found that the approach is robust to the choice of ASR method (see Results). The Panstripe algorithm is similar to root-to-tip regression, which is used to test for temporal signal in phylogenies (Rambaut et al. 2016). However, in contrast to TempEst, the regression is not performed on the root-to-tip distance but rather on the individual branch lengths. This avoids the problematic dependence structure that makes the root-to-tip regression not suitable for statistical hypothesis testing (Drummond et al. 2003; Rambaut et al. 2016).

Panstripe assumes that the number of gene gain and loss events on each branch are distributed according to a compound Poisson distribution. This allows for multiple genes to be gained and lost in a single event (see Methods). Gene duplications are treated as gene gain events and are typically represented by multiple rows in the matrix output of common pangenome inference pipelines. Errors in the gene presence/absence matrix introduced at either the sequencing, assembly, annotation, or pangenome clustering stage are unlikely to correlate with the core genome phylogeny. That is, we expect to see a similar number of errors on shorter terminal branches that originate from a closely related clade as we would on a long terminal branch leading to a taxon with no close relatives. ASR of these errors will place them at the terminal branches (tips) of the phylogeny. By including a binary covariate that indicates whether or not a branch is located at the tip of the phylogeny, we can control for the presence of errors in the pangenome. Differences in the error rates of different data sets will often be reflected in this “tip” covariate. Without careful consideration of the individual gene sequences, rare genes that persist within genomes for periods of time that are much shorter than the length of terminal branches can be difficult to distinguish from errors. As a result, Panstripe groups the signal from both these sources into a single term.

The GLM framework used by Panstripe can also be used to compare the rates of HGT between pangenomes or to identify associations with additional covariates of interest. This relies on the given phylogenies having the same scale, which can be achieved using timescaled trees. Alternatively, SNP-scaled phylogenies can be used if differences in the ratio of core to accessory genome variation are of interest. To test whether the number of genes involved in each recombination event is significantly different between two pangenomes, Panstripe allows different models to be fitted separately to the mean and dispersion structure of the GLM (Smyth 1989).

The Panstripe package provides several plotting functions, including an alternative to the popular pangenome accumulation curve that controls for both errors and differences in the core genome diversity of the underlying isolates. The package is written in R and is available freely under an MIT license (see Software availabilty).

### Panstripe is robust to errors in pangenome clustering

To assess the robustness of the Panstripe algorithm to the choice of pangenome clustering method, we considered the pangenome analysis of a large outbreak of highly clonal Mtb in London spanning 14 yr (Casali et al. 2016). This collection of genomes has been previously analyzed using both the Roary and Panaroo pangenome clustering pipelines (Tonkin-Hill et al. 2020). Because of the very low mutation rate and highly clonal nature of the outbreak, we would expect there to be no pangenome variation in this data set, making it a useful control for assessing whether pangenome inference tools can account for errors.

**Figure 2.**
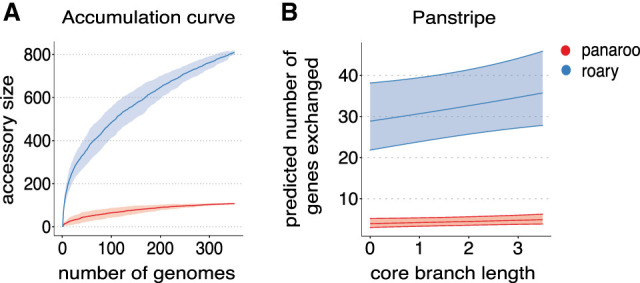
Comparison of methods on a highly clonal *Mycobacterium tuberculosis* (Mtb) outbreak data set. (*A*) Pangenome accumulation curves after running both Roary (blue) and Panaroo (red) on 351 Mtb genomes from an outbreak in London. The ribbon indicates the variation in the curve found by permuting the genome order 100 times. (*B*) Plot of the core branch length versus the predicted number of accessory genes for the same set of Mtb genomes according to the Panstripe model. The ribbon displays the 95% confidence interval of the Panstripe model fit and indicates that the inferred slopes are not significantly different from zero for both the Panaroo and Roary presence–absence matrices.

Figure 2A presents the pangenome accumulation curves of the resulting gene presence/absence matrices output by the Roary and Panaroo pipelines. The very large difference in the two curves shows that the accumulation curve method is highly sensitive to the different error rates of the two pipelines. We have previously shown that Panaroo can significantly reduce the number of errors when generating a pangenome clustering, as observed in Figure 2A. However, Panaroo still estimates a small amount of accessory variation in this clonal Mtb data set.

Figure 2B indicates that the Panstripe algorithm can accurately account for the differences in error rates of the two pipelines. By plotting the core branch lengths against the predicted number of gene gain and loss events, it becomes clear that the large number of accessory genes identified by the Roary pipeline do not correlate with the core phylogeny and thus are likely to be erroneous. Panstripe correctly estimates a very similar association between the core genome branch length and the number of inferred gene gain and loss events on each branch for both the Roary and Panaroo pangenomes. The inferred coefficients of the GLM were found to be β_core_ = 0.0621 and 0.0625, respectively. In both cases, Panstripe found that the coefficients were not significantly different from zero (*P* = 0.871), which is consistent with the closed pangenome of Mtb. Panstripe can also be used to compare the differences in the rate of gene exchange events associated with the tips of the phylogeny. Here, it correctly identifies a significantly elevated rate of errors in the Roary gene presence/absence matrix (*P* < 0.001).

### Panstripe outperforms alternative methods on simulated data

**Figure 3.**
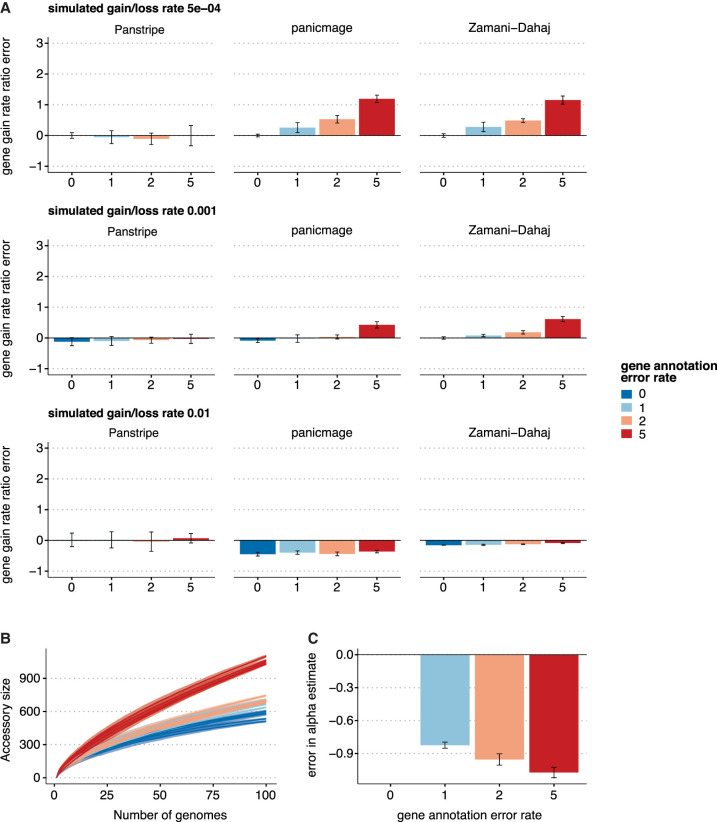
Results of each algorithm on simulated data with different error rates. (*A*) Bars indicate the mean percentage error in the estimated ratio of gene gain and loss rate compared to the reference rate of 5 × 10^−4^ with a simulated annotation error of zero. The three rows represent increasingly large gene gain and loss rates of 5 × 10^−4^, 0.001, and 0.01. The simulated annotation error rates are given along the *x*-axis. (*B*) Pangenome accumulation curves with a simulated gene gain and loss rate of 0.001. The colors represent the increasing annotation error rates. (*C*) The corresponding error in the α parameter estimates after fitting Heaps' law to the curves in *B*. Lower α estimates indicate a more “open” pangenome. Thus, higher rates of annotation error can lead to incorrect estimates of whether a pangenome is open or closed.

Simulations indicate that Panstripe is more robust to the impacts of errors and population structure than alternative methods, including those based on information theory (Tettelin et al. 2005, 2008) and phylogenetically informed approaches (Fig. 3; Supplemental Fig. 1; Baumdicker et al. 2012; Collins and Higgs 2012; Zamani-Dahaj et al. 2016). To simulate pangenome data sets, we first generate core genome phylogenies before simulating the number and size of gene gain and loss events along each branch (see Methods). In line with the assumptions of Dollo's parsimony, we assume an elevated frequency of gene loss relative to gene gain as such an asymmetry is frequently observed in bacterial genomes (Farris 1977; Apagyi et al. 2018). Errors in the pangenome matrix were generated by randomly adding or removing single entries in the matrix.

It is challenging to compare the output of the various pangenome dynamics inference tools as each makes different assumptions and they do not attempt to infer the same set of parameters. To cope with this, we focused on the ability of each tool to accurately quantify differences in the dynamics of gene gain and loss between pangenome data sets. For those tools that infer a gene gain rate, we tested whether the ratio of inferred gene gain rates matched that of the simulations. Thus, we would expect a ratio of the estimated gene gain rate of one for a pair of simulations with the same parameters and a ratio of two if one simulation used a gene gain rate that was twice as large. This allows us to investigate the ability of each tool to distinguish the dynamics of gene gain and loss between data sets. Approaches based on information theory, such as the pangenome accumulation curve and fitting a Heaps' power law, do not have a parameter that is easily compared with the gene gain rate. Instead, we compare the results of these methods using a fixed gene gain and loss rate subject to different levels of error and bias.

Figure 3A indicates that Panstripe was the only tool that provided consistent parameter estimates both at higher error rates and across a range of gene gain and loss rates. The phylogenetic informed methods such as the IMG model of panicmage and the FMG model of Zamani-Dahaj et al. (2016) both performed poorly at higher error rates. Although these methods were generally accurate in the absence of errors in the presence–absence matrix, they both systematically underestimated higher gene gain rates. In our analyses, we could not get the IMG model of Collins and Higgs (2012) to give consistent results (Supplemental Fig. 1). The information theory–based approaches were highly sensitive to errors in the presence–absence matrix (Fig. 3B,C). Increased error rates led to both substantial differences in the slopes of the pangenome accumulation curve and an underestimate in the α parameter of Heaps' power law. This suggests that under realistic error rates, the use of Heaps' power law to classify pangenomes into “open” and “closed” is problematic. To test whether these results held for different ratios of gene gain and loss, we repeated the analysis assuming equal frequencies of gene gain and loss, as well as an elevated rate of gene gain. In both cases, we observed very similar results (Supplemental Figs. 3, 4).

ASR forms a critical component of the Panstripe algorithm. To test the sensitivity of the approach to the choice of algorithm, we simulated multiple pangenome data sets with increasing rates of annotation error. We then ran Panstripe using the included ASR algorithms (maximum parsimony, maximum likelihood, and stochastic mapping). We found that Panstripe performed similarly using all three of the included algorithms with the variation in the estimated rate of gene gain and loss using the same set of simulation parameters exceeding the variation between the different ASR algorithms (Supplemental Fig. 5A). These simulations represent relatively well behaved data sets. When we compared the gene gain and loss estimates on the more challenging clonal Mtb data set, there was a larger difference between the different ASR algorithms, and only maximum parsimony correctly indicated a gene gain and loss rate that was indistinguishable from zero (Supplemental Fig. 5B,C). The very low temporal signal in the Mtb phylogeny leads to very short branch lengths and multichotomies. This causes the maximum likelihood and stochastic mapping algorithms to incorrectly assign gene gain and loss events to longer branches. Consequently, as the major goal of the Panstripe algorithm is to enable robust inference of gene gain and loss rates, we make use of maximum parsimony by default. However, the program includes the option to use both maximum likelihood and stochastic mapping algorithms so that users can easily test the sensitivity of their analyses to the assumptions of these different algorithms.

**Figure 4.**
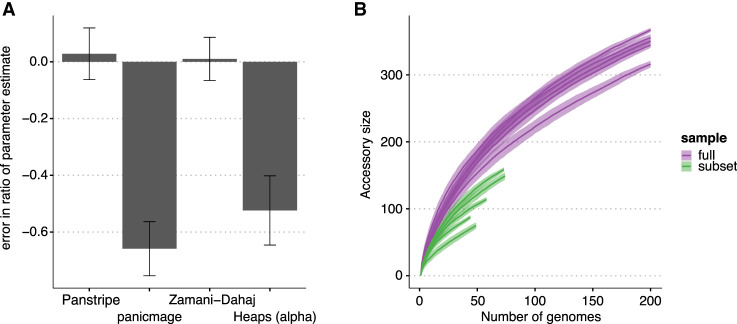
Impact of simulated population structure on each algorithm. (*A*) Bars indicate the mean percentage error in the estimated ratio of gene gain and loss rate in a smaller subclade of the simulated data compared with estimating the rate on the full simulation. Only Panstripe and the method of Zamani-Dahaj et al. (2016) accurately reported similar parameters in both the small and large sets. This suggests that panicmage and fitting a Heaps' power law are highly sensitive to sampling biases in the data. (*B*) Similar to the Heaps' power law, pangenome accumulation curves provide misleading differences when comparing the subclade and full data sets.

We found that similar to the impacts of errors in the pangenome gene presence–absence matrix, Panstripe was more robust to sampling biases and the underlying phylogenetic structure of a pangenome data set. To test this, we simulated five large phylogenies and accompanying gene presence–absence matrices. We then selected a small subclade (30 or more genomes) within each simulation to create a smaller, more closely related data set. Here, the simulation parameters of the large data set and the subclade are the same, and thus, the ratio of the estimated parameter for each method on each data set should be one. Figure 4A indicates that both Panstripe and the phylogenetically informed method of Zamani-Dahaj et al. (2016) provided consistent parameter estimates of the full data set and subclade. Conversely, the ratios of the gene gain rate estimated by panicmage and the Heaps' power law α parameter between the full data set and the subclade both had error rates >50%. Similarly, the pangenome accumulation curves (Fig. 4B) were highly sensitive to sampling bias. Overall, these simulations indicate that Panstripe outperforms other methods by providing gene gain and loss rate estimates that better reflect the true difference between data sets while being robust to both sampling bias and error in the gene presence–absence matrix.

To verify that Panstripe can differentiate between different rates of annotation error, we generated simulated pangenomes using three different gene gain and loss rates and five annotation error rates. Supplemental Figure 2 indicates the resulting *P*-values associated with the tip parameter of the Panstripe GLM. Panstripe was able to accurately differentiate different rates of annotation error for all data sets except those generated using the highest gene gain and loss rate. At the highest gene gain and loss rate, the number of real gene exchange events tends to dominate the impact of errors, and thus, it is more challenging for the Panstripe algorithm to distinguish different rates of annotation error. We also considered whether Panstripe can distinguish between the size of gene exchange events by simulating two gene gain and loss rates and four different recombination sizes. Supplemental Figure 6 shows that Panstripe was able to accurately differentiate between all four simulated recombination sizes.

### Panstripe accurately identifies within- and between-species differences in gene gain and loss rates

**Figure 5.**
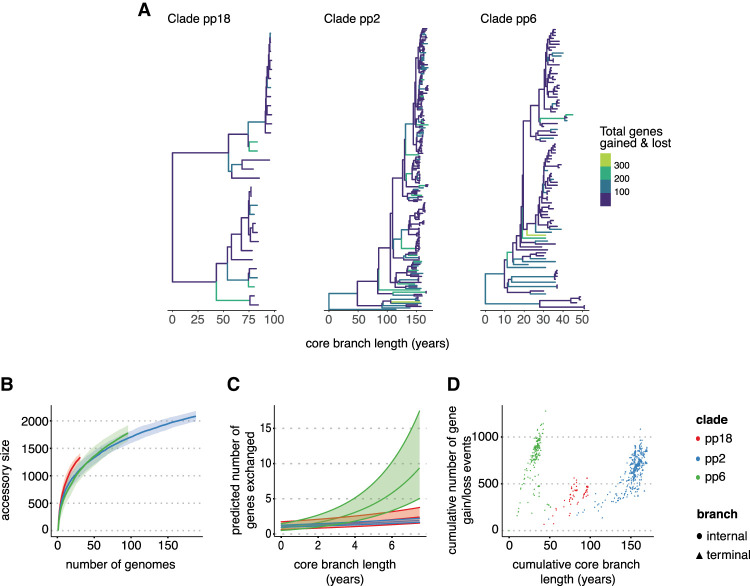
Analysis of the pangenome dynamics of an *E. faecalis* data set. (*A*) Phylogenies of three major *E. faecalis* clades taken from Pöntinen et al. (2021) with branches colored by the number of gene gain and loss events inferred using maximum parsimony. (*B*) The pangenome accumulation curves of the same clades using the pangenomes inferred using Panaroo in Pöntinen et al. (2021). (*C*) The predicted slope of the relationship between core genome branch length and the number of gene gain and loss events is inferred by the Panstripe algorithm. (*D*) The cumulative number of gene gain and loss events versus the cumulative branch length starting from the root node of each tree in *A*. This is a similar plot to the common “root-to-tip” plot used in phylogenetic dating.

To compare the estimates of the Panstripe algorithm on species that are known to have a diverse accessory genome, we considered two previously described data sets. The first included 315 *Enterococcus faecalis* genomes from three major hospital-associated clades sampled in the Netherlands, Spain, and Portugal (Pöntinen et al. 2021). *E. faecalis* is both a commensal and nosocomial pathogen and can successfully inhabit a wide range of host niches. The generalist ecological lifestyle of the microbe is facilitated by a diverse accessory genome with little association between specific niches and particular accessory genes (Palmer et al. 2012; Neumann et al. 2019). Instead, adaptation to the hospital-associated niche is thought to be owing to selection for survivability in a broader set of niches (Pöntinen et al. 2021). We generated pangenome curves and ran the Panstripe algorithm on three of the major hospital-associated *E. faecalis* clades: pp18, pp2, and pp6 (Fig. 5A).

Although clade pp18 (including sequence types ST159 and ST525) appeared to have a larger accessory genome according to the pangenome accumulation curve (Fig. 5B), the Panstripe algorithm revealed that this was likely to be driven by sampling biases as clade pp18 included a more diverse set of genomes (Fig. 5C,D). Instead, the pp6 clade (mainly of ST28) had a higher rate of gene exchange compared with both the pp2 (mainly of ST6) (*P* < 0.001) and pp18 (*P* < 0.001) clades. The pp6 clade is the most recent hospital-associated clade to have emerged in this data set, and thus, the elevated rate of gene exchange could be a result of the additional selection pressures acting within the hospital environment, which may drive the acquisition of antimicrobial resistance plasmids (Pöntinen et al. 2021). Consistent with this hypothesis, Panstripe found that the size of gene gain and loss events was higher in clade pp6.

**Figure 6.**
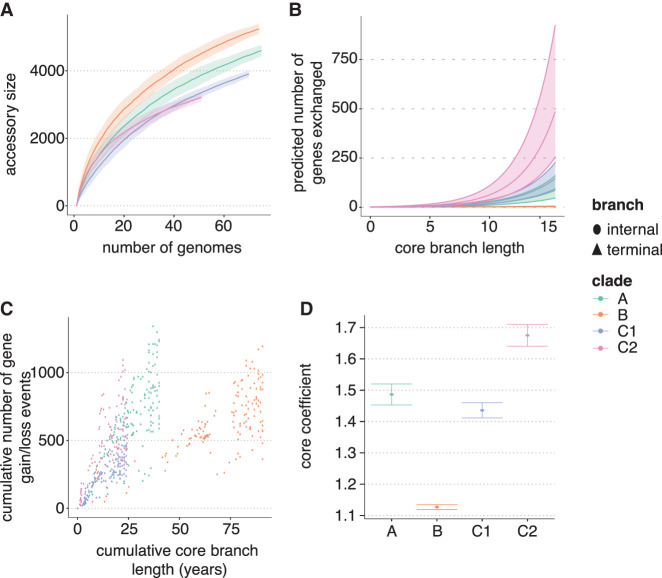
Comparison of the gene gain and loss rate in major clades of *E. coli*. (*A*) Pangenome accumulation curves for the clades in the *E. coli*-ST131 data sets. Pangenome data sets were taken from Gladstone et al. (2021) and were constructed using Panaroo. (*B*) The corresponding predicted slope of the relationship between core genome branch length and the number of gene gain and loss events as inferred by the Panstripe algorithm. (*C*) The cumulative number of gene gain and loss events versus the cumulative branch length starting from the root node of each tree in *A*. This is a similar plot to the common “root-to-tip” plot used in phylogenetic dating. (*D*) The estimated parameters of the generalized linear model used in Panstripe. Error bars represent the 95% confidence interval of the parameter estimates. Higher values of the core coefficient indicate an increased rate of gene gain and loss.

The second data set consisted of four subclades (A, B, C1, C2) of the globally disseminated ST131 clone from a longitudinal study of *Escherichia coli* isolates from the Norwegian surveillance on resistant microbes (NORM) program (Gladstone et al. 2021). Similar to our analysis of *E. faecalis*, the estimated pangenome accumulation curve for these clades gave different results to that produced by Panstripe (Fig. 6A–C). According to the pangenome accumulation curve, clade B showed the largest accessory genome diversity. However, this is likely because of the increased age of clade B as its expansion in the Norwegian population occurred nearly a decade earlier than the other clades (Gladstone et al. 2021). Thus, the pangenome accumulation curve is reflecting the underlying population structure of the data set rather than a difference in the accessory genome dynamics of the clades. Instead, Panstripe predicts an increased rate of gene gain and loss for the C1 and C2 clades, which expanded more recently and have a higher prevalence of drug-resistance loci (Fig. 6D). The emergence of C1 and C2 clades has been shaped by the acquisition of specific F-plasmids involving a series of plasmid gene gains and losses and the exchange of gene modules mediated by the insertion sequence IS26 (Johnson et al. 2016). The increased rate of gains and loss in these two clades is consistent with them acquiring and exchanging multi-drug-resistant (MDR) plasmids more easily than clade A, which has also undergone a recent expansion but is generally more susceptible to different classes of antibiotics (Gladstone et al. 2021).

As we have dated phylogenies for both the *E. faecalis* and *E. coli* data sets, it is possible to use Panstripe to compare the rates of gene gain and loss between the two species. We found that the *E. coli* clades generally had significantly higher rates of gene gain and loss than did the *E. faecalis* clades. This is in agreement with the more generalistic lifestyle of *E. faecalis*. In contrast to the specialization of each of the *E. coli* ST131 clades mediated by the gain and loss of specific MDR plasmids, there was not a significant difference between the *E. faecalis* clades and the *E. coli* clade B (Johnson et al. 2016; Kondratyeva et al. 2020). Our ability to detect smaller differences in the rates of gene exchange is limited by the large differences in the structure of the underlying phylogenies, which leads to increased uncertainty and a reduction in the statistical power to detect differences in these pairwise comparisons.

Although we found small differences in the error rates between the *E. coli* and *E. faecalis* data sets, there was a large difference in the dispersion parameters of the Panstripe GLM. This suggests that the number of genes involved in each gain and loss event differs significantly between the two species. The different mechanisms driving HGT in the two species and the difference in the size of the MGEs could explain this difference (Brockhurst et al. 2019). HGT in *E. coli* is predominantly the result of phage interactions and the exchange of large F-plasmids (size on average >100 kbp), whereas the total plasmid content of *E. faecalis* is generally lower (average ∼24 kbp) (Johnson et al. 2016; Pöntinen et al. 2021). Therefore, *E. coli* has a substantial part of the accessory genome residing on large MGEs, which have shaped the evolution of the subclades, whereas the events of gain and loss for *E. faecalis* may involve smaller MGEs, which allocate fewer genes.

### Improved estimates of associations between phenotypes and pangenome evolutionary dynamics

The GLM framework used in Panstripe allows for other covariates and phenotypes of interest to be easily incorporated into the model and tested to identify significant associations with the rate of gene gain and loss. Common covariates of interest include whether lineages are associated with particular environments such as hospitals, drug resistance, or invasive disease. Previously, we have used the FMG model, as implemented in Panaroo, to estimate the rates of gene gain and loss in 51 major Global Pneumococcal Sequencing Clusters (GPSCs) (Zamani-Dahaj et al. 2016; Gladstone et al. 2019, 2020; Tonkin-Hill et al. 2020). The correlation between the estimated rates and the invasiveness of each lineage was estimated using Spearman's correlation coefficient. This approach identified an association between the rate of gene gain and loss and whether the lineage had a significant odds ratio of invasive disease (Gladstone et al. 2019). Although Panaroo significantly reduced the number of errors, this approach was still sensitive to any remaining errors in the gene presence/absence matrix.

**Figure 7.**
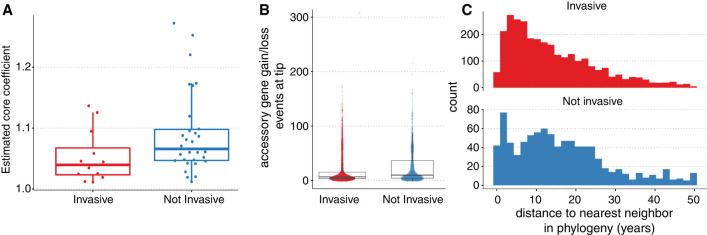
Associations between invasiveness and the dynamics of gene gain and loss in major sequencing clusters of *S. pneumoniae*. (*A*) The estimated coefficient of the core genome parameter of the Panstripe GLM for each of the GPSCs. A higher value indicates an increased rate of gene gain and loss, and invasive GPSCs were found to have a decreased rate of gene exchange. (*B*) The number of accessory genes found to have been either gained or lost at the tips of the core genome phylogenies in each GPSC. (*C*) Histograms of the pairwise patristic distance (in years) between isolates in invasive and noninvasive GPSCs.

To address this, we redid the analysis using the same gene presence/absence matrix inferred by Panaroo but with the Panstripe algorithm. Similar to our previous analysis, Panstripe identified a lower rate of gene gain and loss in GPSCs that had a significant odds ratio of severe disease (*P* < 1 × 10^−3^) (Fig. 7A; Gladstone et al. 2019, 2020; Tonkin-Hill et al. 2020). This could be a signal of genome reduction, which has been linked with pathogenicity across multiple divergence scales (Murray et al. 2021).

Unlike our previous analysis, Panstripe found that this result was sensitive to which branches were included in the analysis with low bootstrap support for the identified association between invasiveness and gene gain and loss rate. Instead, Panstripe identified an enrichment for gene gain and loss events located on terminal branches of invasive lineages (Fig. 7B). A potential explanation is that the annotation error rates differed between severe and nonsevere lineages. However, given that similar sequencing and annotation procedures were used for all genomes in the Global Pneumococcal Sequencing project, this is unlikely. Another explanation is that sampling bias or differences in the underlying population size of the lineages are driving the signal. Most invasive isolates, such as those expressing capsule serotype 1, are known to have outbreak epidemiology (Gladstone et al. 2019). This could lead to a greater sampling of highly related pneumococcal strains from invasive lineages. The greater evolutionary time separating noninvasive lineages would thus allow for a higher number of unique gene exchange events to occur at the tips of the phylogenies, which are more heavily influenced by faster-moving MGEs. This hypothesis is supported by looking at the pairwise patristic distance separating invasive and noninvasive isolates (Fig. 7C). Here, invasive isolates are more closely related, leading to a smaller number of rare unique genes being identified at the tips of the phylogeny. This highlights the utility of the Panstripe algorithm and helps to show its ability to account for annotation errors, population structure, and sampling biases.

## Discussion

Determining the presence/absence of genes in prokaryotic genomes is a complex and error-prone process. Annotation and clustering errors, as well as sampling bias and differences in the underlying population structure of strains and species, can all complicate the analysis of pangenome dynamics. To address these problems, we developed Panstripe, an algorithm that is robust to both errors in the gene presence/absence matrix and the diversity of the underlying genomes. Panstripe can compare the gene exchange rates between pangenomes and determine if these differences are associated with the underlying core genome diversity, rare and erroneous genes occurring at the tips of a phylogeny, or the average size of gene gain and loss events. The use of a GLM framework also allows for associations between gene exchange and covariates of interest to be investigated.

We found that methods that do not account for the diversity of the underlying genomes perform particularly badly, including the commonly used pangenome accumulation curve and Heaps' power law. This was most evident in the analysis of two gene presence/absence matrices obtained after running the Panaroo and Roary pipelines on the same data set. The pangenome accumulation curve incorrectly indicated a diverse pangenome, whereas Panstripe was able to use the additional core genome phylogenetic information to correct for the large difference in error rates of the two pangenome clustering tools. Another common approach is to separate genes into different classes with different rates of gene gain and loss. This is often performed using fixed thresholds indicating the prevalence of a gene within a pangenome, although statistical methods have recently been suggested (Collins and Higgs 2012; Page et al. 2015; Gautreau et al. 2020). Although allowing for different rates of gene gain and loss can improve the fit of a model, these approaches still neglect to account for errors. In the previous example, filtering genes present in <5% of genomes would likely reduce the difference between the Roary and Panaroo algorithms. However, without accounting for the core genome variation, it would be impossible to tell if the filtered genes were erroneous or reflected real biology.

Given a lack of an association between the core genome branch length and the accessory genome, it is tempting to say that such a pangenome is “closed.” However, the binary classification of pangenome into “open” and “closed” can be problematic as it does not incorporate the evolutionary timescale being considered. For example, although there is unlikely to be any gene exchange in an outbreak of Mtb, there is evidence of pangenome variation across the Mycobacterium genus (Rosas-Magallanes et al. 2006; Becq et al. 2007; Chiner-Oms et al. 2019). Instead, we suggest that it is better to report whether the core and accessory genome variation is “coupled” in that there is a significant association between the core and accessory genome diversity in a particular data set.

Although we have shown that Panstripe provides considerable improvements to the analysis of pangenome dynamics, it does not implement a formal evolutionary model. Panstripe also assumes that annotation and clustering errors occur randomly with respect to the core genome phylogeny. Systematic errors that are associated with the underlying population structure may thus be underestimated. This issue can be mitigated when combining different data sets by including the data set as a covariate in the Panstripe model. As most pangenome inference tools are not optimized for metagenome-assembled genomes (MAGs), we did not consider this application. We hope Panstripe provides a substantial improvement over the use of pangenome accumulation curves and allows for simple hypotheses to be tested. As more complex evolutionary models are proposed, we expect that Panstripe will be used similarly to TempEst: as a check for a temporal signal before running more computationally intensive algorithms.

## Methods

### Overview

The Panstripe algorithm takes a binary gene presence/absence matrix and an accompanying core genome phylogeny as input for each pangenome data set being considered. Initially, the ancestral state of the presence of each gene at each node in the core genome phylogeny is inferred using either maximum parsimony or maximum likelihood methods. Using these estimates, the total number of gene gain and loss events on each branch of the phylogeny is calculated. A GLM framework implemented in R is then used to compare the number of gene gain and loss events with the branch length of the core genome phylogeny (R Core Team 2022). Terms are also included in the GLM to indicate the depth of the branch within the phylogeny and whether or not the branch occurs at the tips of the tree. These help to control for the reduced ability to observe gene gain and loss events at higher branches of the tree as well as errors in the gene presence/absence matrix, respectively. Comparisons between pangenomes are made by including an additional categorical covariate in the GLM for the pangenome and investigating the interaction terms between this variable and the core, tip, and depth terms. The Panstripe algorithm scales approximately linearly in the number of genes and number of branches when using the default settings as both the Sankoff's ASR algorithm and the GLM fit are linear in the number of genes and branches, respectively. The resulting runtime is substantially less than the computational time required to run the preceding pangenome clustering steps using tools such as Roary, Panaroo, and PPanGGOLiN (see Supplemental Fig. 7). The size of data sets will thus be limited by these tools.

### Ancestral state reconstruction

Panstripe includes three methods for inferring whether a gene was present or not at each ancestral node of the core genome phylogeny. The default maximum parsimony approach uses a version of Sankoff's dynamic programming algorithm adapted from the Castor R package to determine the ancestral states that correspond with the smallest number of state changes along the phylogeny (Sankoff 1975; Louca and Doebeli 2018). Alternatively, either a maximum likelihood–based method or stochastic mapping can be used. The included maximum likelihood–based method assumes a fixed-rate continuous-time Markov model (Mk model), as implemented in the APE R package (Yang et al. 1995; Paradis et al. 2004). The simulation-free version of stochastic mapping is included as implemented in the SFREEMAP R package v1.1 (Minin and Suchard 2008; Pasqualin et al. 2017). After reconstructing the ancestral state for each gene independently, the total number of gene gain and loss events for each branch is found by taking the sum of events for each gene, assuming that, at most, one change occurs between consecutive nodes.

Although maximum likelihood–based methods can provide improved estimates of the ancestral states in some instances (Pagel 1999), using a maximum likelihood method effectively uses the branch lengths of the core genome phylogeny twice: once in the ASR and once in the regression. This can artificially inflate the correlation between the branch lengths and the number of gene gain and loss events. Consequently, we prefer to use maximum parsimony. In practice, we have found that using either approach gives very similar results in most instances.

### Tweedie generalized linear regression model

After inferring the number of gene gain and loss events, each branch of the phylogeny is subsequently treated as an independent data point. For each branch *i*, let *g*_*i*_ be the number of gene gain and loss events, *l*_*i*_ the core branch length, *d*_*i*_ the core genetic distance from the root of the phylogeny to the node at the start of the branch, and *k*_*i*_ a binary variable indicating whether or not the branch occurred at the tip of the phylogeny.

We assume that the number of gene gain and loss events, *g*_*i*_, follows a compound Poisson distribution. That is, the number of HGT events follows a Poisson distribution, but the size of each event follows a Gamma distribution. This is important as HGT events frequently involve the acquisition or loss of multiple genes. To estimate the association between the number of genes gained and lost and the other covariates, we make use of a Tweedie GLM implemented in the Statmod R package (Dunn and Smyth 2018). The Tweedie distribution generalizes many distributions commonly used in GLMs including the normal, Poisson, and Gamma distributions depending upon the index power parameter *p*. For 1 < *p* < 2, the Tweedie distribution is equivalent to a compound Poisson distribution (Jørgensen 1987).

A Tweedie GLM with a log link function assumes that the expectation of the *i*th response *µ*_i_: = *E*(*g*_*i*_) is related to a vector *x*_*i*_ of covariates with corresponding coefficients *β* by

(1) $$\log(\mu_{i})=x_{i}^{T}\beta \;={\left(l_{i}+k_{i}+d_{i}+l_{i}k_{i}\right)}^{T}\beta .$$

The corresponding variance of *g*_*i*_ is given by

(2) $$var(g_{i})=\mu_{i}^{p}.$$

We include an interaction term between the branch length and whether a branch is terminal to account for the increased chance that a gene is both gained and erroneously omitted on longer terminal branches. For data sets with reliable phylogenies, it is generally safe to assume that annotation errors will be unlikely to propagate to nonterminal branches during ASR. The ability of the algorithm to accurately characterize the gene gain and loss rate can then be improved by setting the intercept of the GLM to zero. This effectively assumes that no gene gain and loss events can occur on internal branches of zero length. If a phylogeny is thought to be unreliable, an intercept can be included to improve the convergence of the algorithm and to account for the mean error of inferred gain and loss events on internal branches being nonzero. This has the side effect of reducing the sensitivity of the approach to detect differences between pangenome data sets. Panstripe allows the user to choose which approach is best suited for their analysis with the intercept excluded by default.

Using this framework, it is possible to perform standard hypothesis tests based on a Student's *t* distribution to determine whether each of the parameters is significantly associated with gene gain and loss. A significant association with the core branch length indicates that there is evidence of HGT at the upper branches of the phylogeny. The association with the “tip” covariate can be driven by a combination of both the error rate in the inference of the gene presence/absence matrix and the acquisition and loss of rare genes that are seen only once. The depth parameter accounts for the potential loss in power to identify HGT at higher branches of the phylogeny. Because of the number of parameters in the GLM, it is recommended that at least 30 genomes are available when running Panstripe.

### Comparing pangenomes

Panstripe uses interaction terms to compare the relationship of the covariates with gene gain and loss between two pangenome data sets. Although the use of a standard GLM framework would allow for the comparison of the inferred slope or β parameters between data sets, it assumes a single dispersion parameter. This effectively fixes the relationship between the rate of gene exchange and the size of each HGT event. This can be seen in the relationship between the Tweedie distribution parameters and the corresponding parameters of the Poisson and Gamma components of the compound Poisson distribution:

$$\lambda =\frac{\mu^{2-p}}{\varphi (2-p)},$$

$$\alpha =\frac{2-p}{\;p-1},$$

$$\theta =\varphi (\;p-1)\mu^{\;p-1}.$$

Here, λ is the mean of the Poisson distribution, and α and θ are the shape and scale parameters of the Gamma distribution, respectively.

To relax this assumption, Panstripe optionally allows for the dispersion parameter to vary between pangenomes using the double generalized linear model (DGLM) framework described by Smyth (1989). The DGLM framework models the mean and dispersion using two separate GLMs with both being a function of the covariates. A maximum likelihood estimate of the parameters is then found by alternating between the two submodels. Setting the pangenome being considered for branch *i* as a binary covariate *n*_*i*_, the DGLM used to compare pangenomes can be formulated as

(3) $$\log(\mu_{i})=x_{i}^{T}\beta_{1}={\left(l_{i}+k_{i}+d_{i}+l_{i}k_{i}+d_{i}n_{i}+k_{i}n_{i}+l_{i}n_{i}+l_{i}k_{i}n_{i}\right)}^{T}\beta_{1},$$

(4) $$\log(\varphi )=z_{i}^{T}\beta_{2}={\left(1+n_{i}\right)}^{T}\beta_{2}.$$

The significance of the inferred coefficients of the interaction terms can be used to determine whether the association between HGT and the depth, core, and tip covariates differs significantly between pangenomes. When investigating the dispersion parameter, Panstripe uses the likelihood ratio test to determine whether there is a significant benefit to accounting for a variable dispersion between two pangenome data sets. In some cases, such as when comparing lineages from the same species, it may be safe to assume that the relationship between the mean and dispersion is similar in both pangenomes. In this case, it can be preferable to fix the dispersion parameter to improve the statistical power of an analysis.

### Identifying associations with additional covariates

The GLM framework used by Panstripe allows for other covariates that may influence pangenome dynamics to be considered. This could include whether or not a lineage is highly drug resistant or hospital associated or is frequently linked to invasive disease. In general, this is most useful when many separate lineages from a large genome collection of a single species are available as is becoming increasingly common with the advent of large sequencing studies and the introduction of WGS as a routine service for public health surveillance (Chattaway et al. 2019; Gladstone et al. 2019, 2020). To investigate such associations, Panstripe replaces the binary pangenome covariate in Equation 3 with the covariate(s) of interest. These can be logical, continuous, or categorical.

### Bootstrap confidence intervals

Panstripe estimates confidence intervals for each coefficient using the bootstrap, by resampling each branch with replacement (Efron 1987). Although it is possible to use the GLM model to obtain confidence interval estimates, this makes some assumptions about the distribution of the coefficients and is more susceptible to outliers. Panstripe uses the boot R package, which implements several commonly used methods for calculating the interval from the resampled set of coefficients, to calculate these intervals (Efron 1987; Davison and Hinkley 1997). In general, we have found the bootstrap confidence intervals to provide similar estimates to that of the GLM model. However, in some cases, differences have indicated a high sensitivity of the result to particular branches. Optionally, Panstripe also includes the possibility of estimating bootstrap *P*-values using the confidence interval inversion method (Hall 2013).

### Simulations

To compare the performance of Panstripe, we simulated core genome phylogenies using the “rtree” function from the APE package (Paradis 2006). The number of gene gain and loss events on each branch was simulated using the simSeq function in the phangorn package (Schliep 2011) with an elevated frequency of gene loss events (base frequency vector of [0.3, 0.2]). To verify that Panstripe is robust to different gene gain/loss ratios, we repeated the analysis with both equal base frequencies and an elevated rate of gene gain events. We assume that each gain or loss event will involve the same set of genes and simulate the size of the set using a Poisson distribution as implemented in R. Errors in the pangenome matrix were simulated by randomly adding or removing single entries in the final gene presence/absence matrix. The number of errors for each genome was simulated using a Poisson distribution. The full set of parameters used in the simulations is given in Supplemental Table 1. All of the code to reproduce the analyses is available in the accompanying GitHub repository.

### Data set preparation

The phylogenies and pangenome gene presence–absence matrices for the *E. coli* and *E. faecalis* data sets were taken directly from the respective publications (Gladstone et al. 2021; Pöntinen et al. 2021). Briefly, in both data sets, clades were defined via alignment-free whole-genome clustering using Population Partitioning Using Nucleotide *K*-mers (Pop-PUNK) v.1.2.2 (Lees et al. 2019). Phylogenies of the resulting clades were then constructed using RAxML v8.2.8 with the GTR+ Gamma rate model after removing recombination using Gubbins v2.4.0. Pangenomes for both data sets were generated using Panaroo v1.2. A more detailed description of the methods can be found in the original publications.

The pangenome of the Mtb data set was taken directly from the publication of the Panaroo algorithm (Panaroo v1.0.0). This included 414 genomes that were very closely related, originally published as part of an analysis into an outbreak of isoniazid-resistant tuberculosis in London (Casali et al. 2016). As our pangenome data set included some genomes that were not included in the phylogeny of the original Mtb publication, we reconstructed a new phylogeny. This was achieved by generating a core genome from the original assemblies using Snippy v4.6 with the “snippy-core” subcommand. Gubbins v2.4.0 was then used to remove poor-quality regions of the alignment, and the final phylogeny was generated using FastTree v2.1.11.

### Software availability

Panstripe is available under an MIT open-source license on GitHub (https://github.com/gtonkinhill/panstripe) and Zenodo (https://zenodo.org/record/6404363). The code, phylogenies, and gene presence/absence matrices used in our analyses are available at GitHub (https://github.com/gtonkinhill/panstripe-manuscript), Zenodo (https://zenodo.org/record/6404403), and as Supplemental Material.

## Supplementary Material

Supplemental Material
