# Supplementary material for: Robust analysis of prokaryotic pangenome gene gain and loss rates with Panstripe
Source: Genome Res. 2023 Jan;33(1):129–40. doi: 10.1101/gr.277340.122 (PMC9977150; doi:10.1101/gr.277340.122)
Supplement: Supplemental Material [file supp_33_1_129__DC1.html]

Robust analysis of prokaryotic pangenome gene gain and loss rates with Panstripe — Robust analysis of prokaryotic pangenome gene gain and loss rates with Panstripe — Supplemental Material 

# Robust analysis of prokaryotic pangenome gene gain and loss rates with Panstripe

## Supplemental Material

- Supplementary\_Material.pdf
- Supplemental\_Code\_0.1.0.tar.gz.zip
