# Supplementary material for: Robust analysis of prokaryotic pangenome gene gain and loss rates with Panstripe
Source: Genome Res. 2023 Jan;33(1):129–40. doi: 10.1101/gr.277340.122 (PMC9977150; doi:10.1101/gr.277340.122)
Supplement: Supplemental Material [file supp_gr.277340.122_Supplementary_Material.pdf]

Supplementary Figures

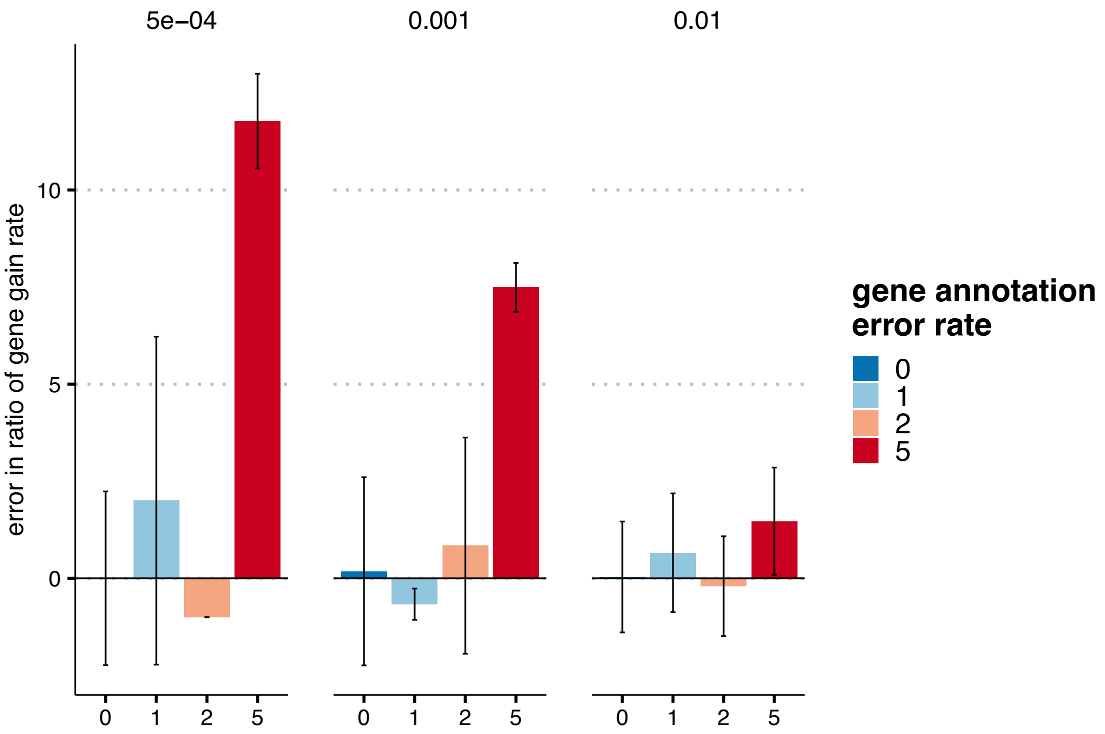

Supplementary Figure 1. Bars indicate the mean percentage error in the estimated ratio of gene gain and loss rate compared to the reference rate of 5×10^{-4} with a simulated annotation error of 0. The R code of Collins et al. gave very inconsistent results as indicated by the very large standard deviation in the estimated ratio (error bars) that often span multiple orders of magnitude. This inconsistency makes it challenging to compare results between runs using this method.

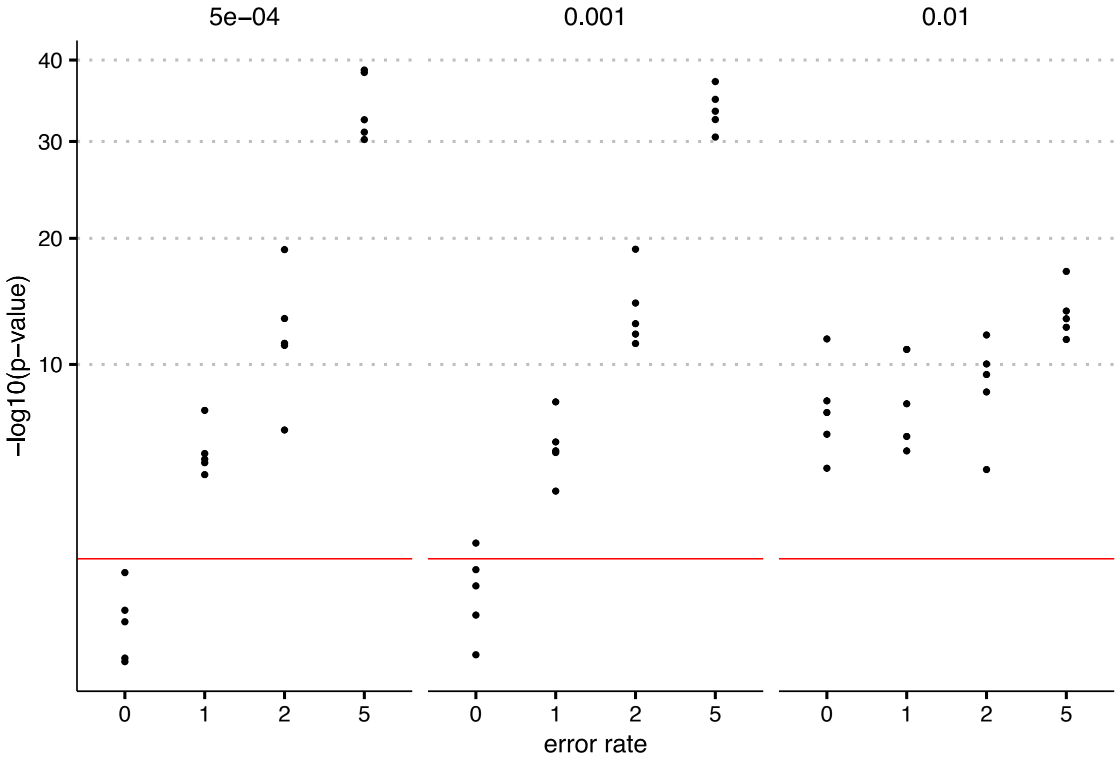

Supplementary Figure 2. P-values for the covariate indicating whether or not a branch occurred at the tip of a phylogeny. Five different error rates were simulated along with four symmetric gene gain/loss rates. Five replicates were simulated for each parameter set. The top row of plots indicates the resulting p-values when comparing each dataset with a pangenome with a mean error rate of 1 while the bottom row shows comparisons with an error rate of 5. The horizontal red line indicates a p-value of 0.05 and points above this line are considered significantly different. Panstripe was less sensitive to differences in error rates when the gene gain and loss rate was very high ($\geq 1e-2$) which is rarely seen in practice.

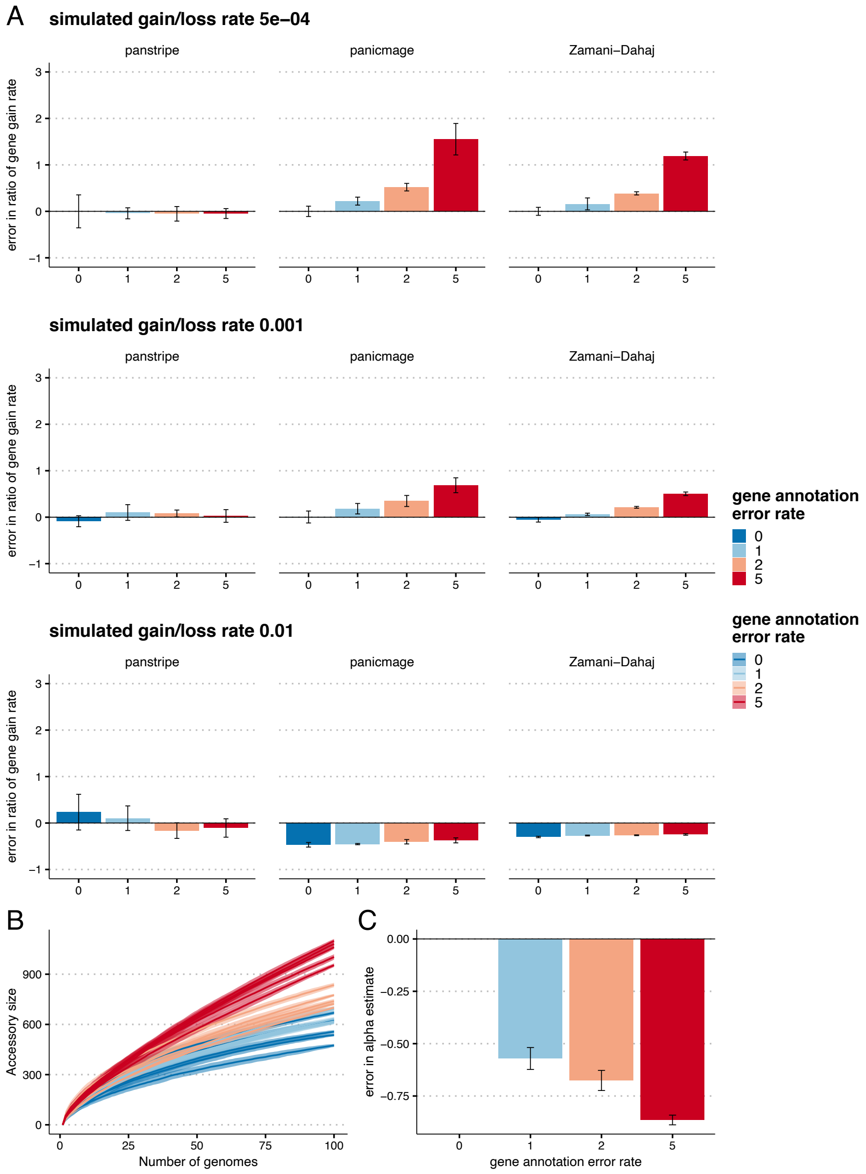

Supplementary Figure 3. This figure reproduces the results of Figure 3 but assumes an even rate of gene gain and loss. **A.)** Bars indicate the mean percentage error in the estimated ratio of gene gain and loss rate compared to the reference rate of 5×10^{-4} with a simulated annotation error of 0. The three rows represent increasingly large gene gain and loss rates of 5×10^{-4}, 0.001 and 0.01. The simulated annotation error rates are given along the x-axis. **B.)** Pangenome accumulation curves with a simulated gene gain and loss rate of 0.001. The colours represent the increasing annotation error rates. **C.)** The corresponding error in the α parameter estimates after fitting Heaps law to the curves in B. Lower α estimates indicate a more 'open' pangenome. Thus, higher rates of annotation error can lead to incorrect estimates of whether a pangenome is open or closed.

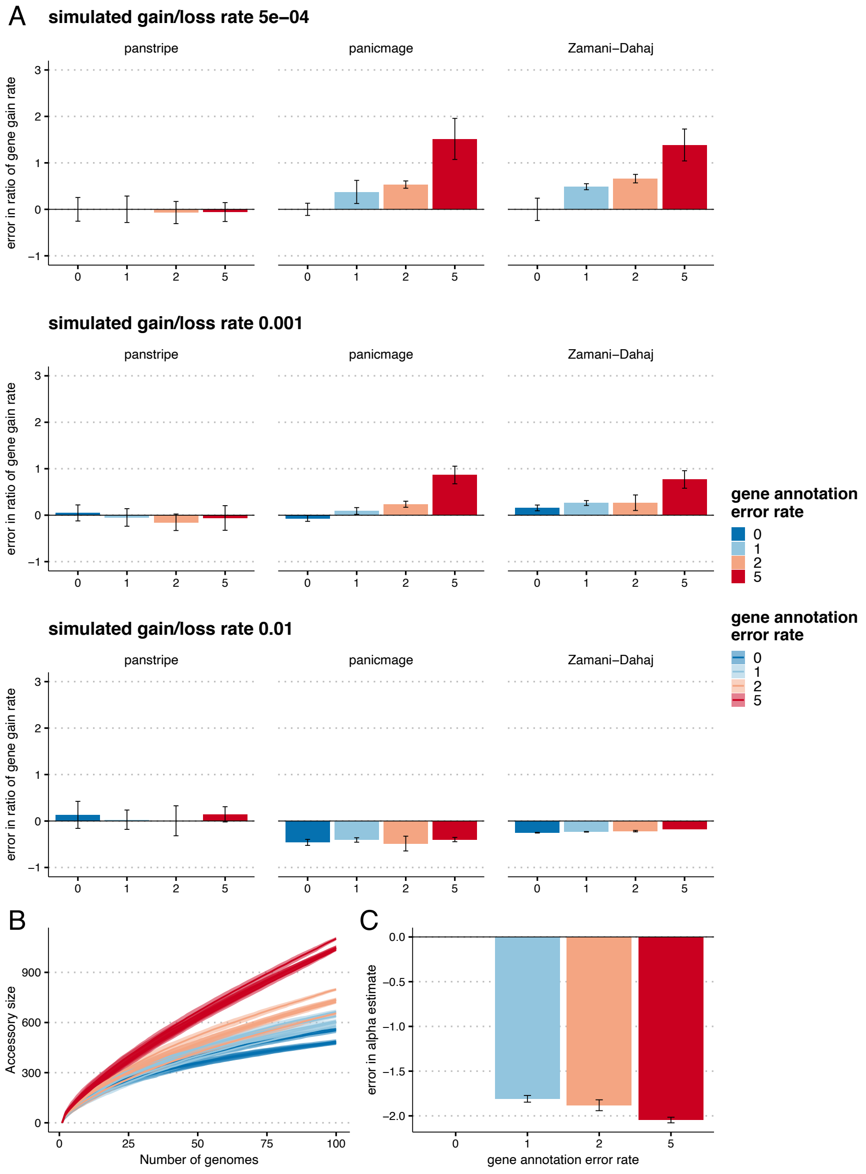

Supplementary Figure 4. This figure reproduces the results of Figure 3 but assumes a greater rate of gene gain relative to gene loss. **A.)** Bars indicate the mean percentage error in the estimated ratio of gene gain and loss rate compared to the reference rate of 5×10^{-4} with a simulated annotation error of 0. The three rows represent increasingly large gene gain and loss rates of 5×10^{-4}, 0.001 and 0.01. The simulated annotation error rates are given along the x-axis. **B.)** Pangenome accumulation curves with a simulated gene gain and loss rate of 0.001. The colours represent the increasing annotation error rates. **C.)** The corresponding error in the α parameter estimates after fitting Heaps law to the curves in B. Lower α estimates indicate a more 'open' pangenome. Thus, higher rates of annotation error can lead to incorrect estimates of whether a pangenome is open or closed.

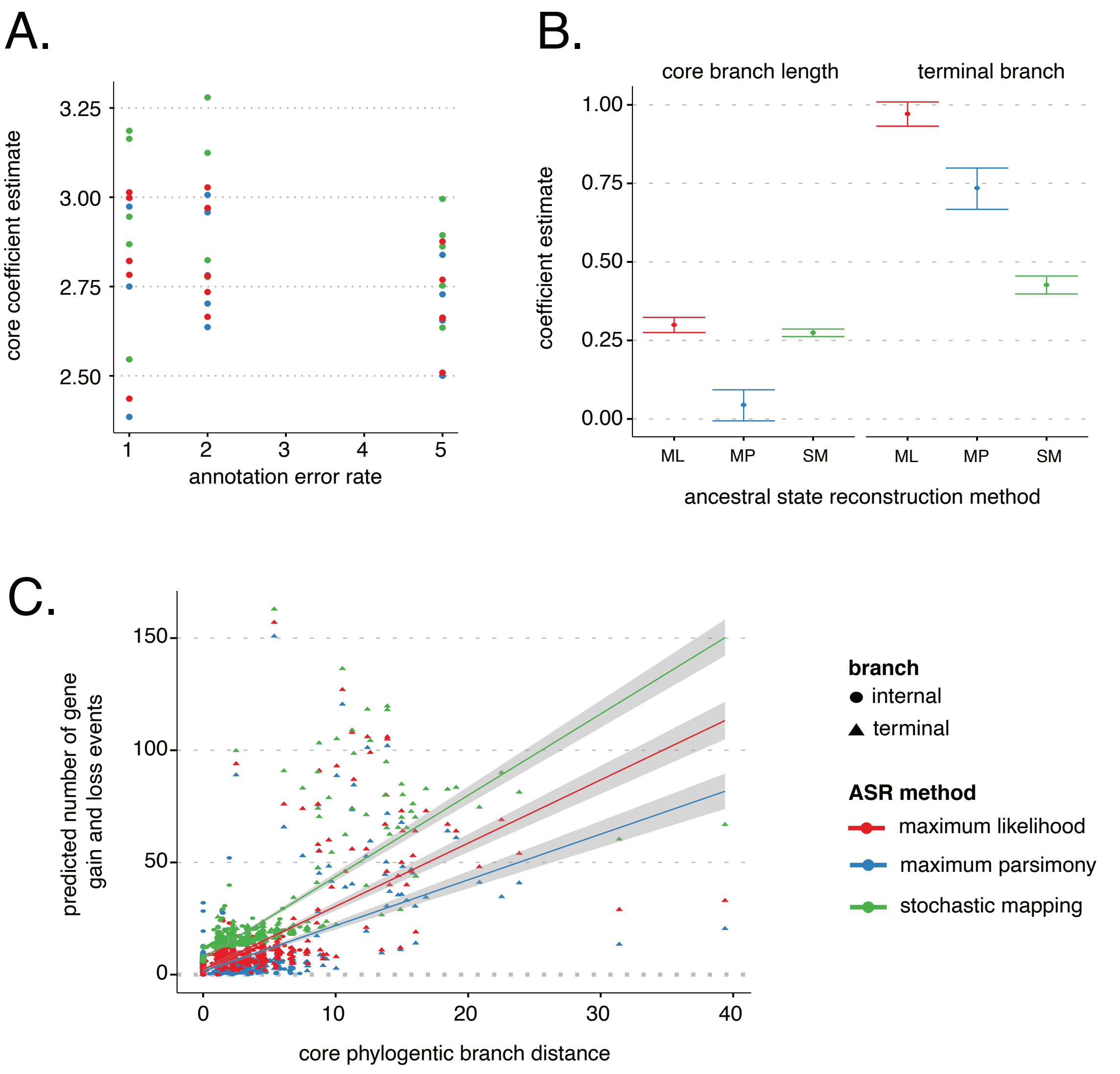

Supplementary Figure 5. A.) The estimated core coefficient of the Panstripe GLM after running the algorithm on simulated pangenomes using a gene gain and loss rate of 1e-3. This was chosen as it provided a sufficient signal to enable a comparison between the different ASR algorithms and resulted in simulations that were similar to real world datasets. Three different gene annotation error rates were also simulated (1, 2 and 5). The colours of the points indicate the ancestral state reconstruction algorithm used. The higher variation within each error rate indicates that the Panstripe algorithm is robust to the choice of ASR algorithm on relatively well-behaved datasets. **B.)** The estimated core and tip coefficients of the Panstripe GLM after running the algorithm on the highly clonal Mtb dataset. Here, the very low core genome diversity made constructing an accurate phylogeny difficult. The resulting phylogeny had very short branch lengths and several multichotomies that led to greater discrepancies between the results of each ASR algorithm. Maximum parsimony, which ignores branch lengths, provided the most reliable result indicating a core coefficient that was not significantly different from zero. **C.)** A dot plot indicating the inferred core genome branch lengths versus the corresponding number of gene gain and loss events inferred to have occurred on each branch using the three ASR algorithms. The lines represent the best fit of a linear model and help to highlight the average differences between the three ASR methods.

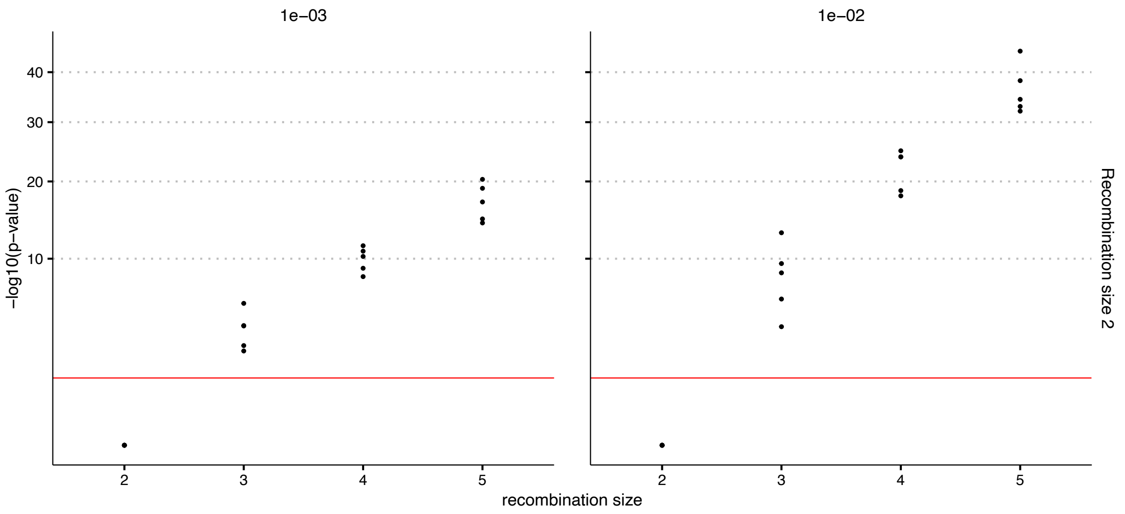

Supplementary Figure 6. P-values obtained after performing a Likelihood Ratio Test to compare a model which allowed the dispersion parameter to vary between pangenomes with a model with a fixed dispersion parameter. The mean number of genes involved in a recombination event was simulated for five different parameter values and each was compared with a mean size of 2. The horizontal red line indicates a p-value of 0.05 and points above this line are considered significantly different.

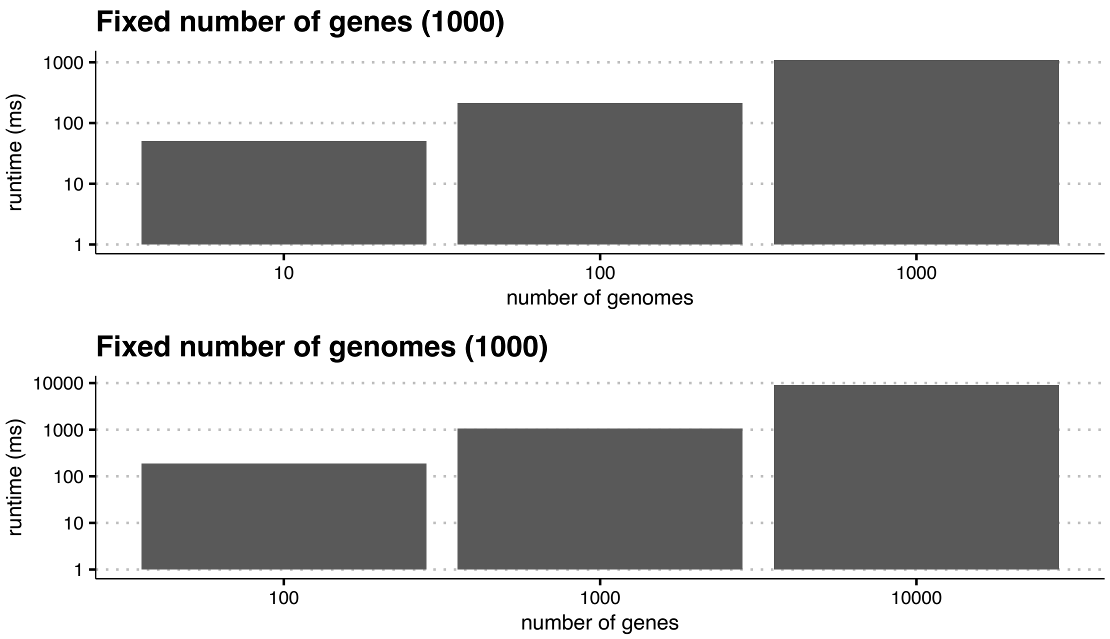

Supplementary Figure 7. A.) The cpu time in milliseconds to run Panstripe (without bootstrap replicates) on datasets with increasing numbers of genomes and a fixed pangenome size. **B.)** Similar to A., but with a fixed number of 1000 genomes and increasing pangenome sizes. Both plots indicate that the algorithm scales approximately linearly with the number of genomes and pangenome size.

Parameter	Method comparison (annotation error)	Method comparison (sampling bias)	Error rate comparisons	Recombination comparisons
Gene gain and loss rate	0.0005, 0.001, 0.01	0.001	0.0005, 0.001, 0.01	0.001, 0.01
Gene annotation error rate	0, 1, 2, 5	0	0, 1, 2, 5	2
Recombination size	3	1	3	2, 3, 4, 5

Supplementary Table 1. The parameters used to compare the results of each method on the simulated pangenome datasets.
