# Supplementary material for: Robust analysis of prokaryotic pangenome gene gain and loss rates with Panstripe
Source: Genome Res. 2023 Jan;33(1):129–40. doi: 10.1101/gr.277340.122 (PMC9977150; doi:10.1101/gr.277340.122)
Supplement: Supplemental Material [file supp_gr.277340.122_Supplemental_Code_0.1.0.tar.gz.zip › panstripe-manuscript-0.1.0/ecoli.html]

E. coli NORM data set


# E. coli NORM data set

```
library(panstripe)
library(tidyverse)
library(data.table)
library(ape)
library(ggthemes)
library(ggtree)
```

## Load data

Load phylogenies and the gene presence/absence matrix from Gladstone et al., 2020.

```
pa <- read_rtab("./data/ecoli/gene_presence_absence.Rtab")
rownames(pa) <- gsub("\\.contigs.*", "", gsub("#", "_", rownames(pa)))

tree_files <- Sys.glob("./data/ecoli/clade*.nwk")
.x <- tree_files[[1]]

clades <- map(tree_files, ~{
    tr <- read.tree(.x)
    tr$tip.label <- gsub("_[0-9]+$", "", tr$tip.label)
    stopifnot(all(tr$tip.label %in% rownames(pa)))
    tpa <- pa[rownames(pa) %in% tr$tip.label, ]
    tpa <- tpa[, colSums(tpa) > 0]
    
    return(list(clade = gsub("_.*", "", gsub(".*ecoli/", "", .x)), tree = tr, pa = tpa))
})
names(clades) <- map_chr(clades, ~.x$clade)
```

## Run panstripe

```
fits <- imap(clades, ~{
    panstripe(.x$pa, .x$tree)
})
```

## Plot results

First we plot the input phylogenies for each clade.

```
patchwork::wrap_plots(imap(clades, ~ggtree(.x$tree) + theme_tree2() + ggtitle(.y)))
```

We first plot a standard accumulation curve. This does not account for differences in error rates and the underlying population structure. Interestingly without controlling for population structure it would be easy to assume clade B was the most diverse. However, we show below that this is not that case which fits much better with previous phylogenetic analysis of the NORM data set.

```
plot_acc(map(clades, ~.x$pa)) + theme_clean(base_size = 20) + theme(plot.background = element_blank(), 
    legend.background = element_blank(), axis.title.x = element_blank())
```

```
ggsave("./figures/ecoli_accumulation_curves.png", width = 12, height = 7)
ggsave("./figures/ecoli_accumulation_curves.pdf", width = 12, height = 7)
```

If we instead plot the panstripe curves we can see that clade B appears to be quite different to the other clades and actually has lower rates of gene gain and loss. This makes sense given it is older and fits with the gain and loss of resistance described in Gladstone et al., 2020.

```
plot_pangenome_fits(fits, include_data = TRUE, trim = FALSE, facet = TRUE)
```

We can confirm this difference by performing pairwise comparisons between the slopes of fitted models. This can be done both for the association with the core branch length and with the tips of the phylogeny. Significant associations with the tips are likely to be driven by both highly mobile elements that do not persist long enough to be observed more than once as well as the effects of annotation errors.

```
comb <- combinat::combn2(1:length(clades))

comparisons <- map2_dfr(comb[, 1], comb[, 2], ~{
    fA <- fits[[.x]]
    fB <- fits[[.y]]
    
    return(compare_pangenomes(fA, fB)$summary %>% add_column(clade1 = names(clades)[[.x]]) %>% 
        add_column(clade2 = names(clades)[[.y]]))
})
```

Look at comparisons of the rate of gene/gain loss at the core genome level

```
pdf <- comparisons %>% filter(term == "core") %>% arrange(p.value)
pdf$p.adj <- p.adjust(pdf$p.value, method = "BH")
knitr::kable(pdf[, c("clade1", "clade2", "statistic", "p.value", "p.adj")])
```

| clade1 | clade2 | statistic | p.value | p.adj |
| --- | --- | --- | --- | --- |
| cladeB | cladeC1 | 5.6183114 | 0.0000000 | 0.0000003 |
| cladeB | cladeC2 | 4.6302382 | 0.0000060 | 0.0000181 |
| cladeA | cladeB | -2.7651666 | 0.0060607 | 0.0121213 |
| cladeC1 | cladeC2 | 2.1426420 | 0.0331967 | 0.0497951 |
| cladeA | cladeC2 | 1.6419633 | 0.1019127 | 0.1222953 |
| cladeA | cladeC1 | 0.1756059 | 0.8607318 | 0.8607318 |

Look at comparisons of the rate of gene/gain loss at the tips of the phylogenies

```
pdf <- comparisons %>% filter(term == "istip") %>% arrange(p.value)
pdf$p.adj <- p.adjust(pdf$p.value, method = "BH")
knitr::kable(pdf[, c("clade1", "clade2", "statistic", "p.value", "p.adj")])
```

| clade1 | clade2 | statistic | p.value | p.adj |
| --- | --- | --- | --- | --- |
| cladeB | cladeC1 | 1.1645386 | 0.2452150 | 0.8487711 |
| cladeA | cladeC1 | 0.7973069 | 0.4259557 | 0.8487711 |
| cladeA | cladeB | -0.6526868 | 0.5144843 | 0.8487711 |
| cladeC1 | cladeC2 | -0.3930507 | 0.6946477 | 0.8487711 |
| cladeB | cladeC2 | 0.3759245 | 0.7073093 | 0.8487711 |
| cladeA | cladeC2 | 0.1163089 | 0.9075054 | 0.9075054 |

Look at comparisons of the rate of gene/gain loss at the tips of the phylogenies

```
pdf <- comparisons %>% filter(term == "istip") %>% arrange(p.value)
pdf$p.adj <- p.adjust(pdf$p.value, method = "BH")
knitr::kable(pdf[, c("clade1", "clade2", "statistic", "p.value", "p.adj")])
```

| clade1 | clade2 | statistic | p.value | p.adj |
| --- | --- | --- | --- | --- |
| cladeB | cladeC1 | 1.1645386 | 0.2452150 | 0.8487711 |
| cladeA | cladeC1 | 0.7973069 | 0.4259557 | 0.8487711 |
| cladeA | cladeB | -0.6526868 | 0.5144843 | 0.8487711 |
| cladeC1 | cladeC2 | -0.3930507 | 0.6946477 | 0.8487711 |
| cladeB | cladeC2 | 0.3759245 | 0.7073093 | 0.8487711 |
| cladeA | cladeC2 | 0.1163089 | 0.9075054 | 0.9075054 |

We can also investigate whether there are differences in the rates of recombination events versus the size of these events between the clades.

```
pdf <- comparisons %>% filter(term == "dispersion model") %>% arrange(p.value)
pdf$p.adj <- p.adjust(pdf$p.value, method = "BH")
knitr::kable(pdf[, c("clade1", "clade2", "statistic", "p.value", "p.adj")])
```

| clade1 | clade2 | statistic | p.value | p.adj |
| --- | --- | --- | --- | --- |
| cladeB | cladeC1 | 10.4105935 | 0.0012529 | 0.0075177 |
| cladeB | cladeC2 | 6.5235600 | 0.0106455 | 0.0319364 |
| cladeA | cladeC1 | 3.6585925 | 0.0557807 | 0.1115615 |
| cladeA | cladeC2 | 2.2526461 | 0.1333861 | 0.2000792 |
| cladeA | cladeB | 1.5078423 | 0.2194686 | 0.2633624 |
| cladeC1 | cladeC2 | 0.0281808 | 0.8666844 | 0.8666844 |

## Compare with *E. faecalis*

```
# load efaelcis fits
efits <- readRDS("./data/efaecalis_fits.RDS")

combined_fits <- c(fits, efits)
```

```
pdf <- plot_pangenome_fits(combined_fits, include_data = TRUE, trim = TRUE, facet = TRUE, 
    plot = FALSE)
pdf$point_data$species <- ifelse(grepl("pp.*", pdf$point_data$pangenome), "E. faecalis", 
    "E. coli")
pdf$fit_data$species <- ifelse(grepl("pp.*", pdf$fit_data$pangenome), "E. faecalis", 
    "E. coli")

ggplot2::ggplot(pdf$fit_data, ggplot2::aes(x = .data$core, y = .data$acc, colour = .data$pangenome)) + 
    ggplot2::geom_point(data = pdf$point_data, ggplot2::aes(y = .data$acc, colour = .data$pangenome), 
        alpha = 1) + ggplot2::geom_line() + ggplot2::geom_ribbon(ggplot2::aes(ymin = .data$lower, 
    ymax = .data$upper, fill = .data$pangenome), alpha = 0.3) + facet_grid(pangenome ~ 
    species) + ggplot2::scale_colour_brewer(type = "qual", palette = 3) + ggplot2::scale_fill_brewer(type = "qual", 
    palette = 3) + theme_clean(base_size = 20) + theme(plot.background = element_blank(), 
    legend.background = element_blank(), axis.title.x = element_blank()) + ggplot2::xlab("core phylogentic branch distance")
```

```
ggsave("./figures/ecoli_efaecalis_pangenome_fits.png", width = 9, height = 15)
ggsave("./figures/ecoli_efaecalis_pangenome_fits.pdf", width = 9, height = 15)
```

```
comb <- combinat::combn2(1:length(combined_fits))

comparisons <- map2_dfr(comb[, 1], comb[, 2], ~{
    fA <- combined_fits[[.x]]
    fB <- combined_fits[[.y]]
    
    return(compare_pangenomes(fA, fB, nboot = 0)$summary %>% add_column(clade1 = names(combined_fits)[[.x]]) %>% 
        add_column(clade2 = names(combined_fits)[[.y]]))
})

pdf <- comparisons %>% filter(term == "core") %>% arrange(p.value)
pdf$p.adj <- p.adjust(pdf$p.value, method = "BH")
knitr::kable(pdf[, c("clade1", "clade2", "estimate", "p.value", "p.adj")])
```

| clade1 | clade2 | estimate | p.value | p.adj |
| --- | --- | --- | --- | --- |
| cladeC1 | pp2 | -0.1179210 | 0.0000000 | 0.0000000 |
| cladeC2 | pp2 | -0.1535755 | 0.0000000 | 0.0000000 |
| cladeC1 | pp18 | -0.1169506 | 0.0000000 | 0.0000000 |
| cladeB | cladeC1 | 0.0957240 | 0.0000000 | 0.0000002 |
| cladeC2 | pp18 | -0.1501964 | 0.0000001 | 0.0000005 |
| cladeA | pp2 | -0.1315701 | 0.0000002 | 0.0000006 |
| cladeC2 | pp6 | -0.1105395 | 0.0000004 | 0.0000013 |
| cladeC1 | pp6 | -0.0746640 | 0.0000025 | 0.0000065 |
| cladeB | cladeC2 | 0.1288566 | 0.0000060 | 0.0000141 |
| cladeA | pp18 | -0.1217259 | 0.0003186 | 0.0006690 |
| pp2 | pp6 | 0.0350401 | 0.0011132 | 0.0021252 |
| cladeA | cladeB | -0.0921177 | 0.0060607 | 0.0106061 |
| cladeA | pp6 | -0.0725774 | 0.0071059 | 0.0114788 |
| pp18 | pp6 | 0.0280971 | 0.0110173 | 0.0165260 |
| cladeC1 | cladeC2 | 0.0511969 | 0.0331967 | 0.0464754 |
| cladeB | pp18 | -0.0193347 | 0.0801598 | 0.1052097 |
| cladeB | pp2 | -0.0095319 | 0.0982654 | 0.1188982 |
| cladeA | cladeC2 | 0.0589160 | 0.1019127 | 0.1188982 |
| cladeB | pp6 | 0.0136439 | 0.2931642 | 0.3240236 |
| cladeA | cladeC1 | 0.0052923 | 0.8607318 | 0.9037684 |
| pp18 | pp2 | 0.0004274 | 0.9575907 | 0.9575907 |

```
pdf <- comparisons %>% filter(term == "istip") %>% arrange(p.value)
pdf$p.adj <- p.adjust(pdf$p.value, method = "BH")
knitr::kable(pdf[, c("clade1", "clade2", "estimate", "p.value", "p.adj")])
```

| clade1 | clade2 | estimate | p.value | p.adj |
| --- | --- | --- | --- | --- |
| cladeC1 | pp2 | -1.2123586 | 0.0000456 | 0.0009575 |
| cladeC2 | pp2 | -0.8540567 | 0.0033857 | 0.0355498 |
| cladeA | pp2 | -0.8634859 | 0.0066134 | 0.0462938 |
| cladeC1 | pp6 | -0.8603867 | 0.0412070 | 0.2163369 |
| cladeB | pp2 | -0.5485022 | 0.0588120 | 0.2470103 |
| cladeC1 | pp18 | -1.0550458 | 0.0712276 | 0.2492967 |
| cladeA | pp6 | -0.6191140 | 0.1748901 | 0.4608102 |
| cladeC2 | pp6 | -0.5546974 | 0.1755467 | 0.4608102 |
| cladeC2 | pp18 | -0.7091404 | 0.2143857 | 0.4626142 |
| cladeA | pp18 | -0.7572843 | 0.2202925 | 0.4626142 |
| cladeB | cladeC1 | 0.5598526 | 0.2452150 | 0.4681378 |
| cladeB | pp6 | -0.4660805 | 0.2723241 | 0.4765672 |
| cladeB | pp18 | -0.5765139 | 0.3175483 | 0.5129626 |
| pp2 | pp6 | 0.2316669 | 0.4086394 | 0.5963380 |
| cladeA | cladeC1 | 0.3998273 | 0.4259557 | 0.5963380 |
| cladeA | cladeB | -0.3210117 | 0.5144843 | 0.6752607 |
| pp18 | pp6 | 0.1678748 | 0.6411253 | 0.7817629 |
| cladeC1 | cladeC2 | -0.1791053 | 0.6946477 | 0.7817629 |
| cladeB | cladeC2 | 0.1844445 | 0.7073093 | 0.7817629 |
| pp18 | pp2 | -0.0987072 | 0.7574540 | 0.7953267 |
| cladeA | cladeC2 | 0.0597987 | 0.9075054 | 0.9075054 |

```
pdf <- comparisons %>% filter(term == "dispersion model") %>% arrange(p.value)
pdf$p.adj <- p.adjust(pdf$p.value, method = "BH")
knitr::kable(pdf[, c("clade1", "clade2", "estimate", "p.value", "p.adj")])
```

| clade1 | clade2 | estimate | p.value | p.adj |
| --- | --- | --- | --- | --- |
| cladeC1 | pp2 | NA | 0.0000000 | 0.0000000 |
| cladeC1 | pp6 | NA | 0.0000000 | 0.0000000 |
| cladeC2 | pp2 | NA | 0.0000000 | 0.0000001 |
| cladeC2 | pp6 | NA | 0.0000002 | 0.0000012 |
| cladeA | pp2 | NA | 0.0000005 | 0.0000020 |
| cladeA | pp6 | NA | 0.0000086 | 0.0000266 |
| cladeC1 | pp18 | NA | 0.0000089 | 0.0000266 |
| cladeB | pp2 | NA | 0.0002078 | 0.0005455 |
| cladeC2 | pp18 | NA | 0.0004596 | 0.0010724 |
| cladeB | cladeC1 | NA | 0.0012529 | 0.0025854 |
| cladeB | pp6 | NA | 0.0013543 | 0.0025854 |
| cladeB | cladeC2 | NA | 0.0106455 | 0.0186296 |
| cladeA | pp18 | NA | 0.0115933 | 0.0187276 |
| cladeA | cladeC1 | NA | 0.0557807 | 0.0836711 |
| cladeA | cladeC2 | NA | 0.1333861 | 0.1867406 |
| cladeB | pp18 | NA | 0.1612102 | 0.2115884 |
| cladeA | cladeB | NA | 0.2194686 | 0.2711083 |
| pp18 | pp6 | NA | 0.2501810 | 0.2918779 |
| pp18 | pp2 | NA | 0.3501297 | 0.3869855 |
| pp2 | pp6 | NA | 0.7860377 | 0.8253396 |
| cladeC1 | cladeC2 | NA | 0.8666844 | 0.8666844 |

## commbined accumulation curve

```
plot_acc(map(combined_fits, ~.x$pa), color_pallete = 3) + theme_clean(base_size = 20) + 
    theme(plot.background = element_blank(), legend.background = element_blank(), 
        axis.title.x = element_blank())
```

```
ggsave("./figures/ecoli_efaecalis_accumulation_curves.png", width = 12, height = 7)
ggsave("./figures/ecoli_efaecalis_accumulation_curves.pdf", width = 12, height = 7)
```
