# Supplementary material for: Robust analysis of prokaryotic pangenome gene gain and loss rates with Panstripe
Source: Genome Res. 2023 Jan;33(1):129–40. doi: 10.1101/gr.277340.122 (PMC9977150; doi:10.1101/gr.277340.122)
Supplement: Supplemental Material [file supp_gr.277340.122_Supplemental_Code_0.1.0.tar.gz.zip › panstripe-manuscript-0.1.0/efaecalis.html]

E. faecalis


# E. faecalis

```
library(panstripe)
library(tidyverse)
library(data.table)
library(ape)
library(ggthemes)
library(ggtree)
library(ggtreeExtra)
```

## Load data

```
pa <- read_rtab("./data/efaecalis/Efcs_gene_presence_absence.Rtab")
rownames(pa) <- gsub("\\.velvet.*", "", rownames(pa))

tree_files <- Sys.glob("./data/efaecalis/*.tre")

clades <- map(tree_files, ~{
    tree <- read.nexus(.x)
    tree$tip.label <- gsub(".*/", "", gsub("_[0-9]_[0-9]+$", "", tree$tip.label))
    name <- gsub(".*/", "", gsub("_.*", "", .x))
    tpa <- pa[rownames(pa) %in% tree$tip.label, ]
    return(list(clade = name, pa = tpa, tree = tree))
})
names(clades) <- map_chr(clades, ~.x$clade)
```

## Plot trees

```
patchwork::wrap_plots(map(clades, ~ggtree(.x$tree) + ggtree::theme_tree2() + ggtitle(.x$clade)))
```

## Run panstripe

```
fits <- map(clades, ~panstripe(.x$pa, .x$tree, quiet = FALSE))
fits$pp18$summary
#> # A tibble: 7 × 7
#>   term   estimate std.error statistic  p.value `bootstrap CI …` `bootstrap CI …`
#>   <chr>     <dbl>     <dbl>     <dbl>    <dbl>            <dbl>            <dbl>
#> 1 Inter…  5.47       1.00       5.47   1.14e-6           3.33            7.60   
#> 2 istip   0.818      0.309      2.65   1.05e-2          -0.102           1.86   
#> 3 core    0.00970    0.0222     0.436  6.64e-1          -0.0509          0.0630 
#> 4 depth  -0.0236     0.0118    -1.99   5.11e-2          -0.0503          0.00460
#> 5 istip… -0.0123     0.0264    -0.465  6.44e-1          -0.156           0.101  
#> 6 p       1.87      NA         NA     NA                 1.03            2.00   
#> 7 phi     1.39      NA         NA     NA                 0.883           4.18
fits$pp2$summary
#> # A tibble: 7 × 7
#>   term   estimate std.error statistic  p.value `bootstrap CI …` `bootstrap CI …`
#>   <chr>     <dbl>     <dbl>     <dbl>    <dbl>            <dbl>            <dbl>
#> 1 Inter…  2.38      0.675        3.53  4.65e-4          1.04              3.64  
#> 2 istip   0.321     0.131        2.45  1.46e-2          0.0371            0.654 
#> 3 core    0.0592    0.0100       5.92  7.34e-9          0.0372            0.0791
#> 4 depth   0.00627   0.00440      1.42  1.55e-1         -0.00188           0.0152
#> 5 istip… -0.0161    0.00827     -1.95  5.20e-2         -0.0511            0.0108
#> 6 p       1.71     NA           NA    NA                1.66              1.76  
#> 7 phi     3.28     NA           NA    NA                2.84              3.99
fits$pp6$summary
#> # A tibble: 7 × 7
#>   term  estimate std.error statistic   p.value `bootstrap CI …` `bootstrap CI …`
#>   <chr>    <dbl>     <dbl>     <dbl>     <dbl>            <dbl>            <dbl>
#> 1 Inte…   4.08      0.401     10.2    1.25e-19           3.34            4.88   
#> 2 istip   0.501     0.224      2.24   2.66e- 2           0.0117          0.982  
#> 3 core    0.0688    0.0200     3.44   7.17e- 4           0.0201          0.110  
#> 4 depth  -0.0314    0.0131    -2.39   1.78e- 2          -0.0583         -0.00411
#> 5 isti…   0.0266    0.0278     0.957  3.40e- 1          -0.0498          0.0982 
#> 6 p       1.66     NA         NA     NA                  1.60            1.74   
#> 7 phi     4.33     NA         NA     NA                  3.68            5.58
saveRDS(fits, "./data/efaecalis_fits.RDS")
```

## Plot results

We first plot a standard accumulation curve. This does not account for differences in error rates and the underlying population structure.

```
plot_acc(map(clades, ~.x$pa)) + theme_clean(base_size = 20) + theme(plot.background = element_blank(), 
    legend.background = element_blank(), axis.title.x = element_blank(), panel.spacing = unit(2, 
        "lines"))
```

```
ggsave("./figures/efaecalis_pangenome_accumulation_curves.png", width = 12, height = 7)
ggsave("./figures/efaecalis_pangenome_accumulation_curves.pdf", width = 12, height = 7)
```

```
plot_pangenome_fits(fits, include_data = TRUE, facet = TRUE) + theme_clean(base_size = 20) + 
    theme(plot.background = element_blank(), legend.background = element_blank(), 
        axis.title.x = element_blank(), panel.spacing = unit(2, "lines"))
```

```
ggsave("./figures/efaecalis_pangenome_fits.png", width = 12, height = 7)
ggsave("./figures/efaecalis_pangenome_fits.pdf", width = 12, height = 7)
```

We can confirm this difference by performing pairwise comparisons between the slopes of fitted models. This can be done both for the association with the core branch length and with the tips of the phylogeny. Significant associations with the tips are likely to be driven by both highly mobile elements that do not persist long enough to be observed more than once as well as the effects of annotation errors.

```
comb <- combinat::combn2(1:length(clades))

comparisons <- map2_dfr(comb[, 1], comb[, 2], ~{
    fA <- fits[[.x]]
    fB <- fits[[.y]]
    
    return(compare_pangenomes(fA, fB, ci_type = "norm")$summary %>% add_column(clade1 = names(clades)[[.x]]) %>% 
        add_column(clade2 = names(clades)[[.y]]))
})
```

Look at comparisons of the rate of gene/gain loss at the core genome level

```
pdf <- comparisons %>% filter(term == "core") %>% arrange(p.value)
pdf$p.adj <- p.adjust(pdf$p.value, method = "BH")
knitr::kable(pdf[, c("clade1", "clade2", "statistic", "p.value", "p.adj")])
```

| clade1 | clade2 | statistic | p.value | p.adj |
| --- | --- | --- | --- | --- |
| pp2 | pp6 | 3.2774014 | 0.0011132 | 0.0033395 |
| pp18 | pp6 | 2.5619733 | 0.0110173 | 0.0165260 |
| pp18 | pp2 | 0.0532086 | 0.9575907 | 0.9575907 |

Look at comparisons of error rates

```
pdf <- comparisons %>% filter(term == "istip") %>% arrange(p.value)
pdf$p.adj <- p.adjust(pdf$p.value, method = "BH")
knitr::kable(pdf[, c("clade1", "clade2", "statistic", "p.value", "p.adj")])
```

| clade1 | clade2 | statistic | p.value | p.adj |
| --- | --- | --- | --- | --- |
| pp2 | pp6 | 0.8269171 | 0.4086394 | 0.757454 |
| pp18 | pp6 | 0.4667151 | 0.6411253 | 0.757454 |
| pp18 | pp2 | -0.3090250 | 0.7574540 | 0.757454 |

Look at comparisons of dispersion

```
pdf <- comparisons %>% filter(term == "dispersion model") %>% arrange(p.value)
pdf$p.adj <- p.adjust(pdf$p.value, method = "BH")
knitr::kable(pdf[, c("clade1", "clade2", "statistic", "p.value", "p.adj")])
```

| clade1 | clade2 | statistic | p.value | p.adj |
| --- | --- | --- | --- | --- |
| pp18 | pp6 | 1.3222926 | 0.2501810 | 0.5251946 |
| pp18 | pp2 | 0.8729870 | 0.3501297 | 0.5251946 |
| pp2 | pp6 | 0.0736902 | 0.7860377 | 0.7860377 |
