# Supplementary material for: Robust analysis of prokaryotic pangenome gene gain and loss rates with Panstripe
Source: Genome Res. 2023 Jan;33(1):129–40. doi: 10.1101/gr.277340.122 (PMC9977150; doi:10.1101/gr.277340.122)
Supplement: Supplemental Material [file supp_gr.277340.122_Supplemental_Code_0.1.0.tar.gz.zip › panstripe-manuscript-0.1.0/figures/fig5.pdf]

To Place or open this file in other applications, it should be re-saved from Adobe Illustrator with the "Create PDF Compatible File" option turned on. This option is in the Illustrator Native Format Options dialog box, which appears when saving an Adobe Illustrator file using the Save As command.

This is an Adobe® Illustrator® File that was saved without PDF Content.
To Place or open this file in other applications, it should be re-saved from Adobe Illustrator with the "Create PDF Compatible File" option turned on. This option is in the Illustrator Native Format Options dialog box, which appears when saving an Adobe Illustrator file using the Save As command.

This is an Adobe® Illustrator® File that was saved without PDF Content.
To Place or open this file in other applications, it should be re-saved from Adobe Illustrator with the "Create PDF Compatible File" option turned on. This option is in the Illustrator Native Format Options dialog box, which appears when saving an Adobe Illustrator file using the Save As command.

To Place or open this file in other applications, it should be re-saved from Adobe Illustrator with the "Create PDF Compatible File" option turned on. This option is in the Illustrator Native Format Options dialog box, which appears when saving an Adobe Illustrator file using the Save As command.

This is an Adobe® Illustrator® File that was saved without PDF Content.
To Place or open this file in other applications, it should be re-saved from Adobe Illustrator with the "Create PDF Compatible File" option turned on. This option is in the Illustrator Native Format Options dialog box, which appears when saving an Adobe Illustrator file using the Save As command.

This is an Adobe® Illustrator® File that was saved without PDF Content.
To Place or open this file in other applications, it should be re-saved from Adobe Illustrator with the "Create PDF Compatible File" option turned on. This option is in the Illustrator Native Format Options dialog box, which appears when saving an Adobe Illustrator file using the Save As command.
