# Supplementary material for: Robust analysis of prokaryotic pangenome gene gain and loss rates with Panstripe
Source: Genome Res. 2023 Jan;33(1):129–40. doi: 10.1101/gr.277340.122 (PMC9977150; doi:10.1101/gr.277340.122)
Supplement: Supplemental Material [file supp_gr.277340.122_Supplemental_Code_0.1.0.tar.gz.zip › panstripe-manuscript-0.1.0/figures/panstripe.drawio.pdf]

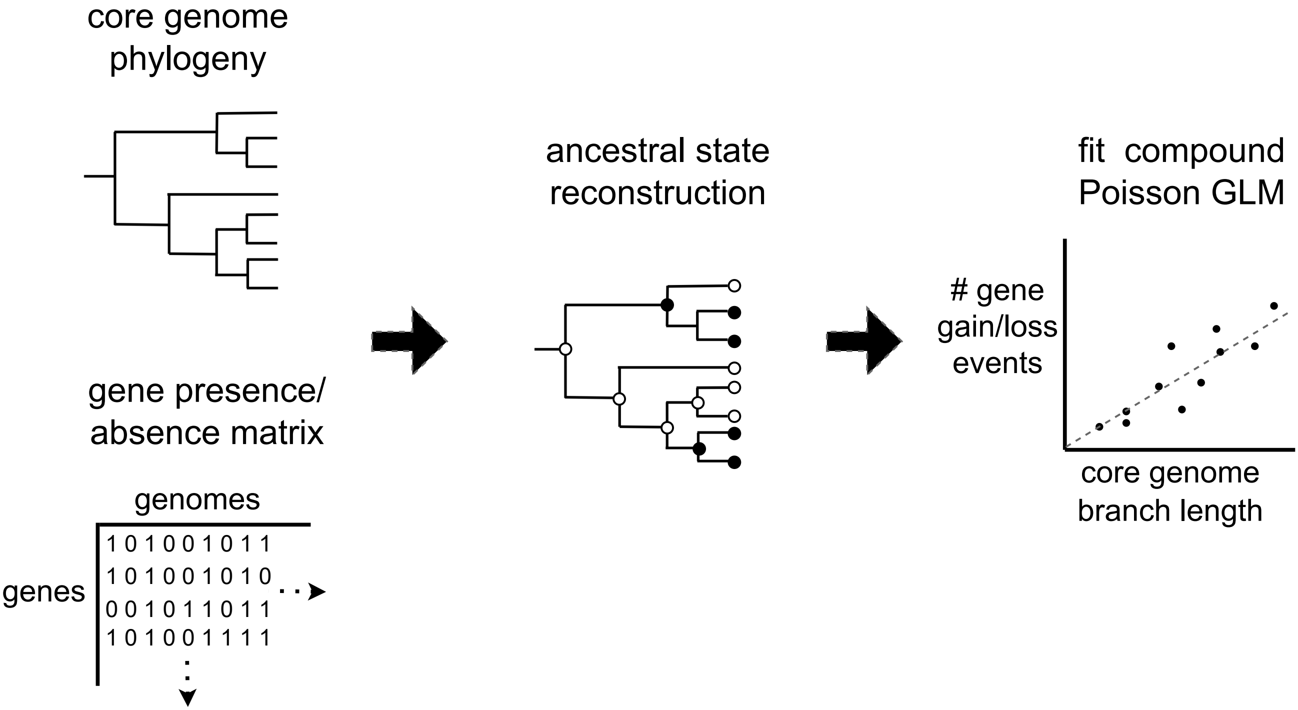
core genome
phylogeny
gene presence/
absence matrix
genomes
genes
1 0 1 0 0 1 0 1 1
1 0 1 0 0 1 0 1 0
0 0 1 0 1 1 0 1 1
1 0 1 0 0 1 1 1 1
ancestral state
reconstruction
fit compound
Poisson GLM
gene
gain/loss
events
core genome
branch length
