# Supplementary material for: Robust analysis of prokaryotic pangenome gene gain and loss rates with Panstripe
Source: Genome Res. 2023 Jan;33(1):129–40. doi: 10.1101/gr.277340.122 (PMC9977150; doi:10.1101/gr.277340.122)
Supplement: Supplemental Material [file supp_gr.277340.122_Supplemental_Code_0.1.0.tar.gz.zip › panstripe-manuscript-0.1.0/figures/simulation_sampling_bias_summary.pdf]

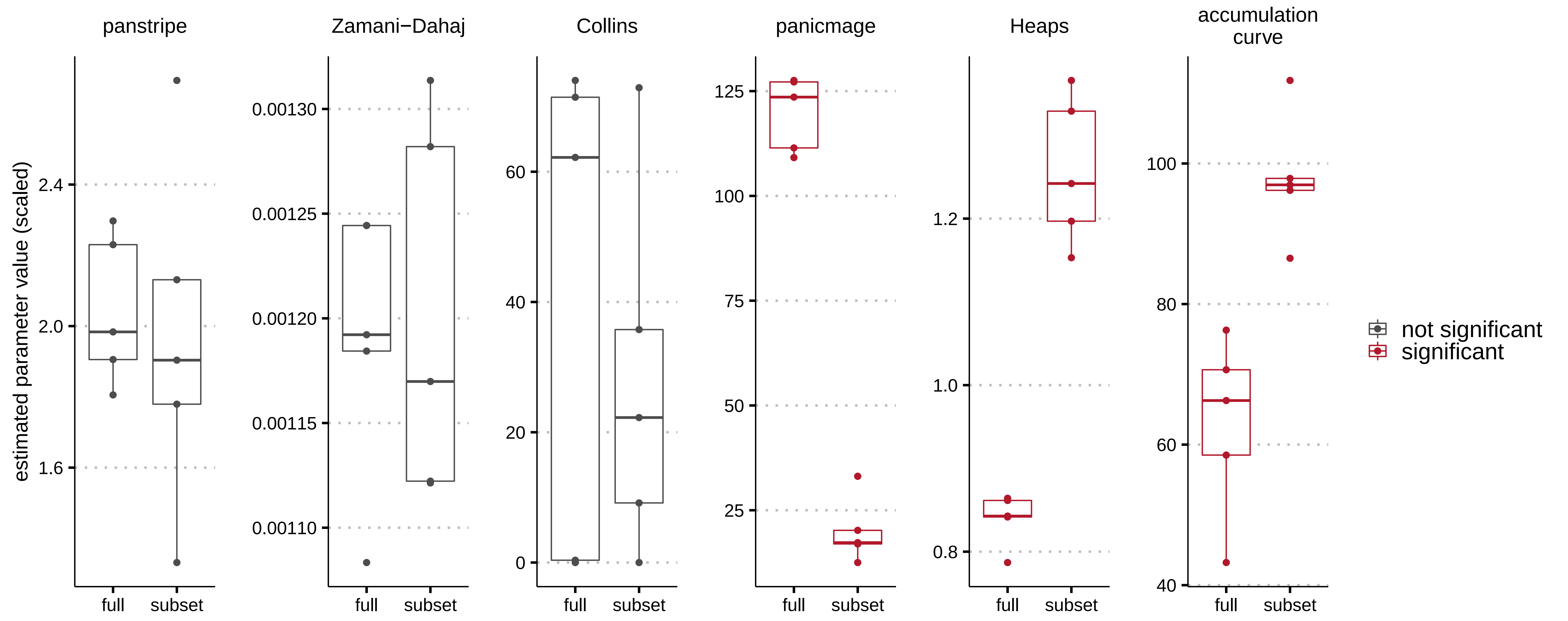
panstripe
Zamani−Dahaj
Collins
panicmage
Heaps
accumulation curve
estimated parameter value (scaled)
2.4
2.0
1.6
0.00130
0.00125
0.00120
0.00115
0.00110
60
40
20
0
125
100
75
50
25
1.2
1.0
0.8
100
80
60
40
full
subset
not significant
significant
