# Supplementary material for: Robust analysis of prokaryotic pangenome gene gain and loss rates with Panstripe
Source: Genome Res. 2023 Jan;33(1):129–40. doi: 10.1101/gr.277340.122 (PMC9977150; doi:10.1101/gr.277340.122)
Supplement: Supplemental Material [file supp_gr.277340.122_Supplemental_Code_0.1.0.tar.gz.zip › panstripe-manuscript-0.1.0/simulated_gain_loss_rates.html]

Method verification - simulated gene gain/loss rates


# Method verification - simulated gene gain/loss rates

```
library(panstripe)
library(tidyverse)
library(data.table)
library(ggthemes)
library(lemon)
library(ape)
```

Here, we considered the ability of the panstripe approach to distinguish different rates of gene gain and loss subject to different error rates.

### Variable gain/loss rate

```
set.seed(123)

error_rates <- c(1, 2, 5, 10)
names(error_rates) <- as.character(error_rates)
gl_rates <- c(1e-04, 5e-04, 0.001, 0.01)
nreps <- 5

params <- expand.grid(error_rates, gl_rates, 1:nreps) %>% as_tibble()
colnames(params) <- c("error rate", "gain loss rate", "replicate")

params$sim <- map2(params$`error rate`, params$`gain loss rate`, ~{
    simulate_pan(rate = .y, mean_trans_size = 3, fn_error_rate = .x, fp_error_rate = .x, 
        ngenomes = 100)
})
```

### Ability to distinguish gain/loss rates

```
rates_to_compare <- c(1e-04, 0.001)
params$fit <- map(params$sim, ~{
    panstripe(.x$pa, .x$tree, nboot = 0, quiet = TRUE)
})

comparisons_res <- map_dfr(split(params, paste(params$replicate, params$`error rate`)), 
    function(rep) {
        map_dfr(rates_to_compare, function(r1) {
            fit1 <- rep$fit[[which(rep$`gain loss rate` == r1)]]
            map_dfr(rep$`gain loss rate`, function(r2) {
                compare_pangenomes(fit1, rep$fit[[which(rep$`gain loss rate` == r2)]], 
                  nboot = 0)$summary %>% add_column(error_rate = unique(rep$`error rate`)) %>% 
                  add_column(`gain loss rate A` = r1) %>% add_column(`gain loss rate B` = r2) %>% 
                  add_column(replicate = unique(rep$replicate))
            })
        })
    })
```

```
pdf <- comparisons_res %>% filter(term == "core")
pdf$`gain loss rate B` <- factor(format(pdf$`gain loss rate B`, digits = 3, scientific = TRUE), 
    levels = format(sort(unique(pdf$`gain loss rate B`)), digits = 3, scientific = TRUE))
pdf$`gain loss rate A` <- format(pdf$`gain loss rate A`, digits = 3, scientific = TRUE)
pdf$error_rate <- factor(paste("Error rate", pdf$error_rate), levels = paste("Error rate", 
    sort(unique(pdf$error_rate))))

ggplot(pdf, aes(x = `gain loss rate B`, y = -log10(p.value))) + geom_jitter(height = 0, 
    width = 0.1) + geom_hline(yintercept = -log10(0.05), col = "red") + lemon::facet_rep_grid(`gain loss rate A` ~ 
    error_rate) + theme_clean(base_size = 16) + theme(plot.background = element_blank(), 
    legend.background = element_blank()) + theme(panel.border = element_blank(), 
    axis.line = element_line()) + # theme(axis.text.x = element_text(angle = 45, hjust=1)) +
scale_y_sqrt() + xlab("symmetric gene gain/loss rate") + ylab("-log10(p-value)")
```

```
ggsave("./figures/gl_rate_comparison.png", width = 10, height = 7)
ggsave("./figures/gl_rate_comparison.pdf", width = 10, height = 7)
```

### Ability to distinguish error rates

```
rates_to_compare <- c(1, 5)

comparisons_err_res <- map_dfr(split(params, paste(params$replicate, params$`gain loss rate`)), 
    function(rep) {
        map_dfr(rates_to_compare, function(r1) {
            fit1 <- rep$fit[[which(rep$`error rate` == r1)]]
            map_dfr(rep$`error rate`, function(r2) {
                compare_pangenomes(fit1, rep$fit[[which(rep$`error rate` == r2)]], 
                  nboot = 0)$summary %>% add_column(`gain loss rate` = unique(rep$`gain loss rate`)) %>% 
                  add_column(`error rate A` = r1) %>% add_column(`error rate B` = r2) %>% 
                  add_column(replicate = unique(rep$replicate))
            })
        })
    })
```

```
pdf <- comparisons_err_res %>% filter(term == "istip")

pdf$`gain loss rate` <- factor(format(pdf$`gain loss rate`, digits = 3, scientific = TRUE), 
    levels = format(sort(unique(pdf$`gain loss rate`)), digits = 3, scientific = TRUE))
pdf$`error rate A` <- factor(paste("Error rate", pdf$`error rate A`), levels = paste("Error rate", 
    sort(unique(pdf$`error rate A`))))
pdf$`error rate B` <- factor(pdf$`error rate B`, levels = sort(unique(pdf$`error rate B`)))

ggplot(pdf, aes(x = `error rate B`, y = -log10(p.value))) + geom_point() + geom_hline(yintercept = -log10(0.05), 
    col = "red") + lemon::facet_rep_grid(`error rate A` ~ `gain loss rate`) + theme_clean(base_size = 20) + 
    theme(plot.background = element_blank(), legend.background = element_blank()) + 
    theme(panel.border = element_blank(), axis.line = element_line()) + scale_y_sqrt() + 
    xlab("error rate") + ylab("-log10(p-value)")
```

```
ggsave("./figures/error_rate_comparison.png", width = 10, height = 7)
ggsave("./figures/error_rate_comparison.pdf", width = 10, height = 7)
```

### Ability to distinguish the rate versus size of gene gain/loss events

```
recomb_size <- c(2, 3, 4, 5)
gl_rates <- c(0.001, 0.01)
nreps <- 5

params <- expand.grid(recomb_size, gl_rates, 1:nreps) %>% as_tibble()
colnames(params) <- c("recomb size", "gain loss rate", "replicate")

params$sim <- map2(params$`recomb size`, params$`gain loss rate`, ~{
    simulate_pan(rate = .y, mean_trans_size = .x, fn_error_rate = 2, fp_error_rate = 2, 
        ngenomes = 200)
})
```

```
params$fit <- map(params$sim, ~{
    panstripe(.x$pa, .x$tree, nboot = 0)
})

rates_to_compare <- c(2)

comparisons_recomb <- map_dfr(split(params, paste(params$replicate, params$`gain loss rate`)), 
    function(rep) {
        map_dfr(rates_to_compare, function(r1) {
            fit1 <- rep$fit[[which(rep$`recomb size` == r1)]]
            map_dfr(rep$`recomb size`, function(r2) {
                compare_pangenomes(fit1, rep$fit[[which(rep$`recomb size` == r2)]], 
                  nboot = 0)$summary %>% add_column(`gain loss rate` = unique(rep$`gain loss rate`)) %>% 
                  add_column(`recomb size A` = r1) %>% add_column(`recomb size B` = r2) %>% 
                  add_column(replicate = unique(rep$replicate))
            })
        })
    })


pdf <- comparisons_recomb %>% filter(term == "dispersion model")

pdf$`gain loss rate` <- factor(format(pdf$`gain loss rate`, digits = 3, scientific = TRUE), 
    levels = format(sort(unique(pdf$`gain loss rate`)), digits = 3, scientific = TRUE))
pdf$`recomb size A` <- factor(paste("Recombination size", pdf$`recomb size A`), levels = paste("Recombination size", 
    sort(unique(pdf$`recomb size A`))))
pdf$`recomb size B` <- factor(pdf$`recomb size B`, levels = sort(unique(pdf$`recomb size B`)))

ggplot(pdf, aes(x = `recomb size B`, y = -log10(p.value))) + geom_point() + geom_hline(yintercept = -log10(0.05), 
    col = "red") + lemon::facet_rep_grid(`recomb size A` ~ `gain loss rate`) + theme_clean(base_size = 20) + 
    theme(plot.background = element_blank(), legend.background = element_blank()) + 
    theme(panel.border = element_blank(), axis.line = element_line()) + scale_y_sqrt() + 
    xlab("recombination size") + ylab("-log10(p-value)")
```

```
ggsave("./figures/recombination_size_comparison.png", width = 15, height = 7)
ggsave("./figures/recombination_size_comparison.pdf", width = 15, height = 7)
```

## Compare impact of changing gain/loss rate on size estiamtes

```
recomb_size <- c(2, 3, 4, 5)
gl_rates <- c(1e-04, 5e-04, 0.001, 0.005, 0.01)
nreps <- 5

params <- expand.grid(recomb_size, gl_rates, 1:nreps) %>% as_tibble()
colnames(params) <- c("recomb size", "gain loss rate", "replicate")

params$sim <- map2(params$`recomb size`, params$`gain loss rate`, ~{
    simulate_pan(rate = .y, mean_trans_size = .x, fn_error_rate = 2, fp_error_rate = 2, 
        ngenomes = 200)
})
```

```
params$fit <- map(params$sim, ~{
    panstripe(.x$pa, .x$tree, nboot = 0)
})

rates_to_compare <- c(0.001, 0.01)

comparisons_recomb_same <- map_dfr(split(params, paste(params$replicate, params$`recomb size`)), 
    function(rep) {
        map_dfr(rates_to_compare, function(r1) {
            fit1 <- rep$fit[[which(rep$`gain loss rate` == r1)]]
            map_dfr(unique(rep$`gain loss rate`)[unique(rep$`gain loss rate`) != 
                r1], function(r2) {
                compare_pangenomes(fit1, rep$fit[[which(rep$`gain loss rate` == r2)]], 
                  nboot = 0)$summary %>% add_column(`recomb size` = unique(rep$`recomb size`)) %>% 
                  add_column(`gain loss rate A` = r1) %>% add_column(`gain loss rate B` = r2) %>% 
                  add_column(replicate = unique(rep$replicate))
            })
        })
    })

pdf <- comparisons_recomb_same %>% filter(term == "dispersion model")

pdf$`gain loss rate A` <- factor(format(pdf$`gain loss rate A`, digits = 3, scientific = TRUE), 
    levels = format(sort(unique(pdf$`gain loss rate A`)), digits = 3, scientific = TRUE))
pdf$`gain loss rate B` <- factor(format(pdf$`gain loss rate B`, digits = 3, scientific = TRUE), 
    levels = format(sort(unique(pdf$`gain loss rate B`)), digits = 3, scientific = TRUE))
pdf$`recomb size` <- factor(paste("Recombination size", pdf$`recomb size`), levels = sort(paste("Recombination size", 
    unique(pdf$`recomb size`))))


ggplot(pdf, aes(x = `gain loss rate B`, y = -log10(p.value))) + geom_point() + geom_hline(yintercept = -log10(0.05), 
    col = "red") + lemon::facet_rep_grid(`gain loss rate A` ~ `recomb size`) + theme_clean(base_size = 20) + 
    theme(plot.background = element_blank(), legend.background = element_blank()) + 
    theme(panel.border = element_blank(), axis.line = element_line()) + scale_y_sqrt() + 
    xlab("Gene gain/loss rate") + ylab("-log10(p-value)")
```

```
ggsave("./figures/recombination_size_comparison_nochange.png", width = 15, height = 7)
ggsave("./figures/recombination_size_comparison_nochange.pdf", width = 15, height = 7)
```
