# Supplementary material for: Robust analysis of prokaryotic pangenome gene gain and loss rates with Panstripe
Source: Genome Res. 2023 Jan;33(1):129–40. doi: 10.1101/gr.277340.122 (PMC9977150; doi:10.1101/gr.277340.122)
Supplement: Supplemental Material [file supp_gr.277340.122_Supplemental_Code_0.1.0.tar.gz.zip › panstripe-manuscript-0.1.0/simulations_error_rates.html]

Method comparison - simulated error rates


# Method comparison - simulated error rates

```
library(panstripe)
library(tidyverse)
library(data.table)
library(ggthemes)
library(ape)
```

## Simulation

Simulate pangenome evolution with sampling error rate.

### Varying error rates

```
set.seed(12345)

rates <- c(0, 1, 2, 5, 10)
names(rates) <- as.character(rates)
nreps <- 5

sim_error <- map(1:nreps, function(rep) map(rates, ~simulate_pan(rate = 0.001, mean_trans_size = 3, 
    fn_error_rate = .x, fp_error_rate = .x, ngenomes = 100)))
names(sim_error) <- 1:nreps
```

Create the necessary input files and run each algorithm

#### panicmage

set up input data

```
imap(sim_error, function(rep, i) {
    imap(rep, ~{
        ape::write.tree(.x$tree, file = paste(c("./data/error_rates/panicmage/error_rate_", 
            .y, "_rep_", i, ".tree"), collapse = ""))
        gc <- table(factor(colSums(.x$pa), levels = 1:nrow(.x$pa)))
        writeLines(paste(gc, collapse = " "), paste(c("./data/error_rates/panicmage/error_rate_", 
            .y, "_rep_", i, ".txt"), collapse = ""))
    })
})
```

run panicmage

```
for f in ./data/error_rates/panicmage/*.tree
do
prefix="${f%.*}"
echo $prefix
~/Documents/panicmage/panicmage ${prefix}.tree ${prefix}.txt 100 -n > ${prefix}_results.txt
done
```

load results

```
resfiles <- Sys.glob("./data/error_rates/panicmage/error_rate_*_results.txt")

panicmage_results <- map_dfr(resfiles, ~{
    l <- read_lines(.x)
    i <- which(grepl("Some characteristics.*", l))
    params <- as.numeric(gsub(".*= ", "", unlist(str_split(l[[i + 1]], " \t "))))
    tibble(error_rate = gsub("_rep.*", "", gsub(".*rate_", "", .x)), rep = gsub("_results.*", 
        "", gsub(".*rep_", "", .x)), theta = params[[1]], tho = params[[2]], core = params[[3]])
})


pdf <- panicmage_results %>% pivot_longer(cols = colnames(panicmage_results)[3:5])
pdf$error_rate <- factor(pdf$error_rate, levels = sort(unique(as.numeric(pdf$error_rate))))

ggplot(pdf, aes(x = error_rate, y = value)) + geom_point() + facet_wrap(~name, scales = "free_y")
```

#### Collins et al.

We first need to convert the newick files into ‘tree table’ format.

```
for f in ./data/error_rates/panicmage/*.tree
do
prefix=$(basename $f)
prefix="${prefix%.*}"
echo $prefix
perl ~/Documents/pangenome/tre2table.pl $f > ./data/error_rates/collins/${prefix}.txt
done
```

We can now run the scripts from Collins et al., 2012.

```
source("./scripts/f-pangenome.R")

# coalescent
mymaxit <- 10000
myreltol <- 1e-06
mymodel <- "coalescent.spec"  #use the coalescent tree w/G(k)
myfitting <- "chi2"  #fit it using this error function
constr <- 1  # G0 constrained to the mean genome size during fitting
mymethod <- "Nelder"

collins_results <- imap_dfr(sim_error, function(rep, i) {
    imap_dfr(rep, ~{
        mat <- t(.x$pa)
        ng <- nrow(.x$pa)
        G0 <- mean(colSums(mat > 0))  # mean genome size measured in gene families
        treetable <- read.table(paste(c("./data/error_rates/collins/error_rate_", 
            .y, "_rep_", i, ".txt"), collapse = ""), sep = "\t", row.names = 1)
        colnames(treetable) <- c("desc_a", "desc_b", "dist")
        # calculate gene family frequency spectrum
        Gk <- f.getspectrum(mat)
        # set initial parameters and recursively optimize
        opt.spec.cde <- f.recurse(c(1, 100), r.data = Gk, r.genomesize = G0, r.ng = ng)
        # get optimized parameters, calculate constrained parameter
        params.spec.cde <- c(opt.spec.cde$par[1], opt.spec.cde$par[1] * (G0 - opt.spec.cde$par[2]), 
            opt.spec.cde$par[2])
        # set initial parameters and recursively optimize
        pinitial.spec.c2de <- c(params.spec.cde[1]/10, params.spec.cde[2]/100, params.spec.cde[3], 
            params.spec.cde[1] * 10000)
        opt.spec.c2de <- f.recurse(pinitial.spec.c2de, r.data = Gk, r.genomesize = G0, 
            r.ng = ng)
        
        # get optimized parameters, calculate constrained parameter
        params.spec.c2de <- c(opt.spec.c2de$par, opt.spec.c2de$par[4] * (G0 - opt.spec.c2de$par[2]/opt.spec.c2de$par[1] - 
            opt.spec.c2de$par[3]))
        print(params.spec.c2de)
        spec.c2de <- f.coalescent.spec(params.spec.c2de, ng)
        
        # summarize paramters
        return(tibble(error_rate = .y, rep = i, Gess = params.spec.c2de[3], theta1 = params.spec.c2de[2], 
            rho1 = params.spec.c2de[1], theta2 = params.spec.c2de[5], rho2 = params.spec.c2de[4], 
            fslow = params.spec.c2de[2]/params.spec.c2de[1]/G0, ffast = params.spec.c2de[5]/params.spec.c2de[4]/G0, 
            fess = params.spec.c2de[3]/G0, Gnew100 = f.coalescent(params.spec.c2de, 
                100)$pan[100] - f.coalescent(params.spec.c2de, 100)$pan[99], Gnew1000 = f.coalescent(params.spec.c2de, 
                1000)$pan[1000] - f.coalescent(params.spec.c2de, 1000)$pan[999], 
            Gcore100 = f.coalescent(params.spec.c2de, 100)$core[100], Gcore1000 = f.coalescent(params.spec.c2de, 
                1000)$core[1000], Gpan100 = f.coalescent(params.spec.c2de, 100)$pan[100], 
            Gpan1000 = f.coalescent(params.spec.c2de, 1000)$pan[1000]))
    })
})

pdf <- collins_results[, 1:10] %>% pivot_longer(cols = colnames(collins_results)[3:10])
pdf$error_rate <- factor(pdf$error_rate, levels = sort(unique(as.numeric(pdf$error_rate))))
```

Plot parameter estimates

```
ggplot(pdf, aes(x = error_rate, y = value)) + geom_point() + facet_wrap(~name, scales = "free_y")
```

#### CAFE

CAFE was originally designed for analysing the rates of gain and loss in gene families and is not currently optimised for investigating the size of the bacterial pangenome. However, it is the only model considered that accounts for annotation errors. In this analysis we assume that all genes belong to a single ‘family’.

We provide the error file using the same distribution as was used in the original simulation.

```
# imap(sim_error, function (rep, i) { imap(rep, ~{ ape::write.tree(.x$tree, file
# = paste(c('./data/error_rates/CAFE/error_rate_', .y,'_rep_',i, '.tree'),
# collapse = '')) gc <- rowSums(.x$pa) writeLines(c(paste(c('Desc', 'Family ID',
# names(gc)), collapse = '\t'), paste(c('NA', 'allD', gc), collapse = '\t')),
# paste(c('./data/error_rates/CAFE/error_rate_', .y,'_rep_',i,
# '_gene_families.txt'), collapse = '')) }) })
```

Generate error files.

```
# max_genes <- ceiling(1.2*max(map_dbl(sim_error, ~ max(map_dbl(.x, function(r)
# ncol(r$pa)))))) map(rates, ~{ p <- dexp(x=1, rate = 1/.x) error_tbl <- tibble(
# 'cntdiff'=0:max_genes, '-1'=c(0, rep(p, max_genes)), '0'= c(1-p, rep(1-2*p,
# max_genes)), '1'= rep(p, max_genes+1) ) file <-
# paste(c('./data/error_rates/CAFE/error_model_rate_', .x,'.txt'), collapse = '')
# writeLines(paste0('maxcnt:', max_genes), file) write.table(error_tbl, file =
# file, append = TRUE, quote = FALSE, sep = ' ', row.names = FALSE) })
```

Run CAFE

```
~/Documents/CAFE5-5.0/bin/cafe5 -i ./data/error_rates/CAFE/error_rate_10_rep_1_gene_families.txt -t ./data/error_rates/CAFE/error_rate_10_rep_1.tree -e./data/error_rates/CAFE/error_model_rate_10.txt
```

#### Finitely Many Genes model

Here we make use of the implementation of the FMG model as described in Zamani-Dahaj et al., 2016 and implemented in the Panaroo package.

```
imap(sim_error, function(rep, i) {
    imap(rep, ~{
        ape::write.tree(.x$tree, file = paste(c("./data/error_rates/zamani-dahaj/error_rate_", 
            .y, "_rep_", i, ".tree"), collapse = ""))
        tb <- as_tibble(t(.x$pa)) %>% add_column(gene = colnames(.x$pa), .before = 1)
        write.table(tb, sep = "\t", quote = FALSE, row.names = FALSE, file = paste(c("./data/error_rates/zamani-dahaj/error_rate_", 
            .y, "_rep_", i, "_pa.txt"), collapse = ""))
    })
})
```

```
for f in ./data/error_rates/zamani-dahaj/*.tree
do
prefix=$(basename $f)
prefix="${prefix%.*}"
echo $prefix
python ~/Documents/panaroo/panaroo-estimate-fmg.py -o ./data/error_rates/zamani-dahaj/${prefix}.txt --pa ./data/error_rates/zamani-dahaj/${prefix}_pa.txt --tree $f
done
```

Load results

```
resfiles <- Sys.glob("./data/error_rates/zamani-dahaj/error_rate_*[0-9].txt")

zamani_results <- map_dfr(resfiles, ~{
    df <- fread(.x, skip = 4, header = FALSE, col.names = c("parameter", "estimate", 
        "NA1", "NA2"))[, 1:2] %>% as_tibble() %>% add_column(error_rate = gsub("_rep.*", 
        "", gsub(".*rate_", "", .x)), .before = 1) %>% add_column(rep = gsub("\\.txt", 
        "", gsub(".*rep_", "", .x)), .before = 1)
    return(df)
})
zamani_results$error_rate <- factor(zamani_results$error_rate, levels = sort(unique(as.numeric(zamani_results$error_rate))))

pdf <- zamani_results %>% group_by(error_rate, rep) %>% summarise(parameter = c("u", 
    "v"), estimate = c(estimate[which(parameter == "a")] * estimate[which(parameter == 
    "M")], estimate[which(parameter == "v")]))

ggplot(pdf, aes(x = error_rate, y = estimate)) + geom_point() + facet_wrap(~parameter)
```

#### Accumulation curves (overlapping)

Accumulation curves do not use a statistical model by are very commonly used. Uncertainty is typically quantified using permutations. Here, we consider two curves to be different if there is no overlap in their curves generated by permutation.

```
nperm <- 100
curves <- imap_dfr(sim_error, function(rep, i) {
    print(i)
    return(imap_dfr(rep, ~{
        .x <- t(.x$pa)
        plotdf <- purrr::map_dfr(1:nperm, function(i) {
            ppa <- .x[sample(nrow(.x), replace = FALSE), sample(ncol(.x), replace = FALSE)]
            cumlative <- rowSums(apply(ppa, 1, cumsum) > 0)
            cumlative <- cumlative - cumlative[[1]]
            df <- tibble::tibble(N = 1:length(cumlative), naccessory = cumlative, 
                permutation = i)
            return(df)
        }) %>% tibble::add_column(pangenome = .y)
        
        plotdf <- plotdf %>% dplyr::group_by(N, pangenome) %>% dplyr::summarise(`accessory size` = mean(naccessory), 
            std = sd(naccessory)) %>% add_column(rep = i, .before = 1) %>% add_column(error_rate = .y, 
            .before = 1)
        return(plotdf)
    }))
})
#> [1] "1"
#> [1] "2"
#> [1] "3"
#> [1] "4"
#> [1] "5"

curves$group <- paste(curves$error_rate, curves$rep, sep = "_")
curves$error_rate <- factor(curves$error_rate, levels = sort(unique(as.numeric(curves$error_rate))))

ggplot(curves, aes(N, `accessory size`, col = error_rate, fill = error_rate, group = group)) + 
    geom_ribbon(aes(ymin = `accessory size` - std, ymax = `accessory size` + std), 
        alpha = 0.5, col = NA) + scale_color_brewer(type = "qual", palette = 5) + 
    scale_fill_brewer(type = "qual", palette = 5) + geom_line(size = 1) + theme_bw(base_size = 14) + 
    xlab("Number of genomes") + ylab("Accessory size") + labs(fill = "error rate", 
    color = "error rate")
```

### Heaps Law approach of Tettlin et al.

```
heap_df <- function(pa) {
    cm <- do.call(rbind, map(1:10, ~{
        ppa <- pa
        ppa <- ppa[sample(nrow(ppa), replace = FALSE), sample(ncol(ppa), replace = FALSE)]
        cumlative <- rowSums(apply(t(ppa), 1, cumsum) > 0)
        cumlative <- cumlative - cumlative[[1]]
        return(cumlative)
    }))
    cumulative_median <- apply(cm, 2, median)
    
    res <- broom::tidy(lm(nunique ~ logN, tibble(logN = log(1:length(cumulative_median)), 
        nunique = log(cumulative_median + 0.001))))
    return(res)
}

heap_results <- imap_dfr(sim_error, function(rep, i) {
    return(imap_dfr(rep, ~{
        pdf <- heap_df(.x$pa) %>% add_column(rep = i, .before = 1) %>% add_column(error_rate = as.numeric(.y), 
            .before = 1)
    }))
    return(pdf)
})

heap_results$term <- ifelse(heap_results$term == "logN", "alpha", "log(K)")
heap_results <- heap_results %>% filter(term == "alpha")
```

```
heap_results$error_rate <- factor(heap_results$error_rate, levels = sort(unique(heap_results$error_rate)))
ggplot(heap_results, aes(x = error_rate, y = estimate, col = rep)) + geom_point()
```

#### Panstripe

```
pp_results <- imap_dfr(sim_error, function(rep, i) {
    return(imap_dfr(rep, ~{
        fit <- panstripe(.x$pa, .x$tree, nboot = 0, quiet = TRUE, family = "gaussian")
        pdf <- fit$summary %>% add_column(rep = i, .before = 1) %>% add_column(error_rate = .y, 
            .before = 1)
        return(pdf)
    }))
})

pp_results$error_rate <- factor(pp_results$error_rate, levels = sort(unique(as.numeric(pp_results$error_rate))))
pp_results <- pp_results %>% filter(term == "core")
```

```
ggplot(pp_results, aes(x = error_rate, y = estimate, col = rep)) + geom_point()
```

####Summary plot

```
# panicmage
pannic_res <- panicmage_results %>% pivot_longer(cols = colnames(panicmage_results)[3:5]) %>% 
    filter(name == "theta")
pannic_res$value <- c(scale(pannic_res$value, scale = FALSE, center = FALSE))

pannic_test <- do.call(rbind, pannic_res %>% filter(error_rate > 0) %>% group_by(error_rate) %>% 
    group_map(~{
        broom::tidy(t.test(pannic_res$value[pannic_res$error_rate == 0], .x$value, 
            alternative = "two.sided")) %>% add_column(error_rate = unique(.x$error_rate), 
            .before = 1)
    }, .keep = TRUE))
pannic_test
#> # A tibble: 4 × 11
#>   error_rate estimate estimate1 estimate2 statistic   p.value parameter conf.low
#>   <chr>         <dbl>     <dbl>     <dbl>     <dbl>     <dbl>     <dbl>    <dbl>
#> 1 1             -10.2      131.      142.     -1.15   2.94e-1      5.85    -31.9
#> 2 10           -292.       131.      424.    -25.4    1.66e-6      5.03   -322. 
#> 3 2             -45.9      131.      177.     -8.42   3.02e-5      7.99    -58.5
#> 4 5            -145.       131.      276.     -7.74   1.04e-3      4.37   -195. 
#> # … with 3 more variables: conf.high <dbl>, method <chr>, alternative <chr>

# collins
collins_res <- collins_results[, 1:10] %>% pivot_longer(cols = colnames(collins_results)[3:10]) %>% 
    filter(name == "theta1")
collins_res$value <- c(scale(collins_res$value, scale = FALSE, center = FALSE))

collins_test <- do.call(rbind, collins_res %>% filter(error_rate > 0) %>% group_by(error_rate) %>% 
    group_map(~{
        broom::tidy(t.test(collins_res$value[collins_res$error_rate == 0], .x$value, 
            alternative = "two.sided")) %>% add_column(error_rate = unique(.x$error_rate), 
            .before = 1)
    }, .keep = TRUE))
collins_test
#> # A tibble: 4 × 11
#>   error_rate   estimate estimate1 estimate2 statistic p.value parameter conf.low
#>   <chr>           <dbl>     <dbl>     <dbl>     <dbl>   <dbl>     <dbl>    <dbl>
#> 1 1            -5.40e-4  0.000107   6.46e-4    -0.987 0.378        4.07 -2.05e-3
#> 2 10           -3.09e+2  0.000107   3.09e+2    -8.46  0.00107      4.00 -4.11e+2
#> 3 2            -1.41e+2  0.000107   1.41e+2    -3.99  0.0162       4.00 -2.39e+2
#> 4 5            -1.80e+2  0.000107   1.80e+2    -8.41  0.00109      4.00 -2.40e+2
#> # … with 3 more variables: conf.high <dbl>, method <chr>, alternative <chr>

# Zamani-Dahaj
zamani_res <- zamani_results %>% filter(parameter == "v")
zamani_res$estimate <- c(scale(zamani_res$estimate, scale = FALSE, center = FALSE))
zamani_res$error_rate <- as.numeric(as.character(zamani_res$error_rate))

zamani_test <- do.call(rbind, zamani_res %>% filter(error_rate > 0) %>% group_by(error_rate) %>% 
    group_map(~{
        broom::tidy(t.test(zamani_res$estimate[zamani_res$error_rate == 0], .x$estimate, 
            alternative = "two.sided")) %>% add_column(error_rate = unique(.x$error_rate), 
            .before = 1)
    }, .keep = TRUE))
zamani_test
#> # A tibble: 4 × 11
#>   error_rate  estimate estimate1 estimate2 statistic  p.value parameter conf.low
#>        <dbl>     <dbl>     <dbl>     <dbl>     <dbl>    <dbl>     <dbl>    <dbl>
#> 1          1 -0.000316   0.00203   0.00234     -2.77  2.47e-2      7.80 -5.80e-4
#> 2          2 -0.000690   0.00203   0.00272     -5.18  9.13e-4      7.81 -9.99e-4
#> 3          5 -0.00166    0.00203   0.00369    -11.5   5.47e-6      7.42 -2.00e-3
#> 4         10 -0.00357    0.00203   0.00560    -23.1   6.95e-8      7.03 -3.93e-3
#> # … with 3 more variables: conf.high <dbl>, method <chr>, alternative <chr>

# accumulation curves
curves_res <- curves %>% filter(N > 1)
curves_res <- map_dfr(split(curves_res, curves_res$N), ~{
    .x$value = c(scale(.x$`accessory size`, scale = FALSE, center = FALSE))
    return(.x)
})

curves_res <- curves_res %>% pivot_wider(id_cols = c("rep", "N"), names_from = "error_rate", 
    values_from = "value")

curve_test <- map_dfr(4:7, ~{
    broom::tidy(ks.test(curves_res$`0`, unlist(curves_res[, .x]), alternative = "two.sided")) %>% 
        add_column(error_rate = colnames(curves_res)[[.x]])
})
curve_test
#> # A tibble: 4 × 5
#>   statistic  p.value method                             alternative error_rate
#>       <dbl>    <dbl> <chr>                              <chr>       <chr>     
#> 1     0.251 6.46e-14 Two-sample Kolmogorov-Smirnov test two-sided   1         
#> 2     0.398 0        Two-sample Kolmogorov-Smirnov test two-sided   2         
#> 3     0.578 0        Two-sample Kolmogorov-Smirnov test two-sided   5         
#> 4     0.711 0        Two-sample Kolmogorov-Smirnov test two-sided   10

# Heaps
heap_results$value <- scale(heap_results$estimate, scale = FALSE, center = FALSE)
heap_results$error_rate <- as.numeric(as.character(heap_results$error_rate))
heap_test <- do.call(rbind, heap_results %>% filter(error_rate > 0) %>% group_by(error_rate) %>% 
    group_map(~{
        broom::tidy(t.test(heap_results$value[heap_results$error_rate == 0], .x$value, 
            alternative = "two.sided")) %>% add_column(error_rate = unique(.x$error_rate), 
            .before = 1)
    }, .keep = TRUE))


# panstripe
pp_results$value <- scale(pp_results$estimate, scale = FALSE, center = FALSE)
pp_results$error_rate <- as.numeric(as.character(pp_results$error_rate))
panstripe_test <- do.call(rbind, pp_results %>% filter(error_rate > 0) %>% group_by(error_rate) %>% 
    group_map(~{
        broom::tidy(t.test(pp_results$value[pp_results$error_rate == 0], .x$value, 
            alternative = "two.sided")) %>% add_column(error_rate = unique(.x$error_rate), 
            .before = 1)
    }, .keep = TRUE))

panstripe_test
#> # A tibble: 4 × 11
#>   error_rate estimate estimate1 estimate2 statistic p.value parameter conf.low
#>        <dbl>    <dbl>     <dbl>     <dbl>     <dbl>   <dbl>     <dbl>    <dbl>
#> 1          1   0.209       12.2      12.0    0.161    0.876      7.82    -2.80
#> 2          2   0.588       12.2      11.6    0.514    0.621      7.92    -2.05
#> 3          5   0.0664      12.2      12.1    0.0482   0.963      7.55    -3.15
#> 4         10   0.493       12.2      11.7    0.435    0.675      7.89    -2.13
#> # … with 3 more variables: conf.high <dbl>, method <chr>, alternative <chr>

tests <- tibble(method = rep(c("panicmage", "Zamani-Dahaj", "Collins", "accumulation\ncurve", 
    "Heaps", "panstripe"), c(nrow(pannic_test), nrow(zamani_test), nrow(collins_test), 
    nrow(curve_test), nrow(heap_test), nrow(panstripe_test))), t.statistic = c(pannic_test$statistic, 
    zamani_test$statistic, collins_test$statistic, curve_test$statistic, heap_test$statistic, 
    panstripe_test$statistic), p.value = c(pannic_test$p.value, zamani_test$p.value, 
    collins_test$p.value, curve_test$p.value, heap_test$p.value, panstripe_test$p.value), 
    error.rate = c(pannic_test$error_rate, zamani_test$error_rate, collins_test$error_rate, 
        curve_test$error_rate, heap_test$error_rate, panstripe_test$error_rate))

curves_res_subset <- curves_res %>% filter(N == 20)
pdf <- tibble(values = c(pp_results$value, pannic_res$value, zamani_res$estimate, 
    collins_res$value, heap_results$value, curves_res_subset$`0`, curves_res_subset$`1`, 
    curves_res_subset$`2`, curves_res_subset$`5`, curves_res_subset$`10`), error.rate = c(pp_results$error_rate, 
    pannic_res$error_rate, zamani_res$error_rate, collins_res$error_rate, heap_results$error_rate, 
    rep(c(0, 1, 2, 5, 10), each = nrow(curves_res_subset))), method = rep(c("panstripe", 
    "panicmage", "Zamani-Dahaj", "Collins", "Heaps", "accumulation\ncurve"), c(nrow(pp_results), 
    nrow(pannic_res), nrow(zamani_res), nrow(collins_res), nrow(heap_results), 5 * 
        nrow(curves_res_subset))))

pdf$error.rate <- factor(pdf$error.rate, levels = sort(unique(as.numeric(pdf$error.rate))))
pdf$method <- factor(pdf$method, levels = c("panstripe", "Zamani-Dahaj", "Collins", 
    "panicmage", "Heaps", "accumulation\ncurve"))


pdf$is.sig <- ifelse(paste(pdf$method, pdf$error.rate) %in% paste(tests$method, tests$error.rate)[tests$p.value < 
    0.05], "significant", "not significant")

ggplot(pdf, aes(x = error.rate, y = values, col = is.sig)) + geom_boxplot(outlier.colour = NA, 
    position = position_dodge(width = 0.8)) + geom_point(size = 2, position = position_dodge(width = 0.8)) + 
    theme_clean(base_size = 20) + facet_wrap(~method, scales = "free", nrow = 1) + 
    scale_color_manual(values = c("#4d4d4d", "#b2182b")) + theme(plot.background = element_blank(), 
    legend.background = element_blank(), axis.title.x = element_blank(), panel.spacing = unit(2, 
        "lines")) + # scale_alpha_discrete(guide = 'none') +
ylab("estimated parameter value") + labs(color = "")
```

```
ggsave("./figures/simulation_error_rate_summary.png", width = 17, height = 7)
ggsave("./figures/simulation_error_rate_summary.pdf", width = 17, height = 7)
```
