# Supplementary material for: Robust analysis of prokaryotic pangenome gene gain and loss rates with Panstripe
Source: Genome Res. 2023 Jan;33(1):129–40. doi: 10.1101/gr.277340.122 (PMC9977150; doi:10.1101/gr.277340.122)
Supplement: Supplemental Material [file supp_gr.277340.122_Supplemental_Code_0.1.0.tar.gz.zip › panstripe-manuscript-0.1.0/simulations_sampling_bias.html]

Method comparison - simulated sampling bias


# Method comparison - simulated sampling bias

```
library(panstripe)
library(tidyverse)
library(data.table)
library(ape)
library(ggthemes)
```

## Simulation

Simulate pangenome evolution with sampling error rate.

### Simulate sampling bias

```
set.seed(12345)

nreps <- 5
sim_bias <- map(1:nreps, ~{
    print(.x)
    tsim <- simulate_pan(rate = 0.001, mean_trans_size = 1, fn_error_rate = 0, fp_error_rate = 0, 
        ngenomes = 200)
    size <- 0
    i <- 1
    subset <- tsim
    nodes <- sample(tsim$tree$Nnode + 2:tsim$tree$Nnode)
    while ((size <= 30) || (size > 100)) {
        n <- nodes[[i]]
        subset$tree <- ape::extract.clade(tsim$tree, n)
        size <- length(subset$tree$tip.label)
        i <- i + 1
    }
    
    subset$pa <- subset$pa[rownames(subset$pa) %in% subset$tree$tip.label, ]
    subset$pa <- subset$pa[, colSums(subset$pa) > 0]
    
    return(list(full = tsim, subset = subset))
})
#> [1] 1
#> [1] 2
#> [1] 3
#> [1] 4
#> [1] 5

names(sim_bias) <- 1:nreps
```

Create the necessary input files and run each algorithm

#### panicmage

set up input data

```
imap(sim_bias, function(rep, i) {
    imap(rep, ~{
        ape::write.tree(.x$tree, file = paste(c("./data/sampling_bias/panicmage/", 
            nrow(.x$pa), "_bias_", .y, "_rep_", i, ".tree"), collapse = ""))
        gc <- table(factor(colSums(.x$pa), levels = 1:nrow(.x$pa)))
        writeLines(paste(gc, collapse = " "), paste(c("./data/sampling_bias/panicmage/", 
            nrow(.x$pa), "_bias_", .y, "_rep_", i, ".txt"), collapse = ""))
    })
})
```

run panicmage

```
for f in ./data/sampling_bias/panicmage/*.tree
do
prefix="${f%.*}"
num=$(basename $f)
num="$(cut -d'_' -f1 <<<"$num")"
echo $prefix
~/Documents/panicmage/panicmage ${prefix}.tree ${prefix}.txt $num -n > ${prefix}_results.txt
done
```

load results

```
resfiles <- Sys.glob("./data/sampling_bias/panicmage/*_results.txt")

panicmage_results <- map_dfr(resfiles, ~{
    print(.x)
    l <- read_lines(.x)
    i <- which(grepl("Some characteristics.*", l))
    params <- as.numeric(gsub(".*= ", "", unlist(str_split(l[[i + 1]], " \t "))))
    tibble(set = gsub("_rep.*", "", gsub(".*bias_", "", .x)), rep = gsub("_results.*", 
        "", gsub(".*rep_", "", .x)), theta = params[[1]], tho = params[[2]], core = params[[3]])
})


pdf <- panicmage_results %>% pivot_longer(cols = colnames(panicmage_results)[3:5])

ggplot(pdf, aes(x = set, y = value)) + geom_point() + facet_wrap(~name, scales = "free_y")
```

#### Collins et al.

We first need to convert the newick files into ‘tree table’ format.

```
for f in ./data/sampling_bias/panicmage/*.tree
do
prefix=$(basename $f)
prefix="${prefix%.*}"
echo $prefix
perl ~/Documents/pangenome/tre2table.pl $f > ./data/sampling_bias/collins/${prefix}.txt
done
```

We can now run the scripts from Collins et al., 2012.

```
source("./scripts/f-pangenome.R")

# coalescent
mymaxit <- 10000
myreltol <- 1e-06
mymodel <- "coalescent.spec"  #use the coalescent tree w/G(k)
myfitting <- "chi2"  #fit it using this error function
constr <- 1  # G0 constrained to the mean genome size during fitting
mymethod <- "Nelder"

collins_results <- imap_dfr(sim_bias, function(rep, i) {
    imap_dfr(rep, ~{
        mat <- t(.x$pa)
        ng <- nrow(.x$pa)
        G0 <- mean(colSums(mat > 0))  # mean genome size measured in gene families
        f <- Sys.glob(paste(c("./data/sampling_bias/collins/*bias_", .y, "_rep_", 
            i, ".txt"), collapse = ""))
        print(f)
        treetable <- read.table(f, sep = "\t", row.names = 1)
        colnames(treetable) <- c("desc_a", "desc_b", "dist")
        # calculate gene family frequency spectrum
        Gk <- f.getspectrum(mat)
        # set initial parameters and recursively optimize
        opt.spec.cde <- f.recurse(c(1, 100), r.data = Gk, r.genomesize = G0, r.ng = ng)
        # get optimized parameters, calculate constrained parameter
        params.spec.cde <- c(opt.spec.cde$par[1], opt.spec.cde$par[1] * (G0 - opt.spec.cde$par[2]), 
            opt.spec.cde$par[2])
        # set initial parameters and recursively optimize
        pinitial.spec.c2de <- c(params.spec.cde[1]/10, params.spec.cde[2]/100, params.spec.cde[3], 
            params.spec.cde[1] * 10000)
        opt.spec.c2de <- f.recurse(pinitial.spec.c2de, r.data = Gk, r.genomesize = G0, 
            r.ng = ng)
        
        # get optimized parameters, calculate constrained parameter
        params.spec.c2de <- c(opt.spec.c2de$par, opt.spec.c2de$par[4] * (G0 - opt.spec.c2de$par[2]/opt.spec.c2de$par[1] - 
            opt.spec.c2de$par[3]))
        print(params.spec.c2de)
        spec.c2de <- f.coalescent.spec(params.spec.c2de, ng)
        
        # summarise paramters
        return(tibble(set = .y, rep = i, Gess = params.spec.c2de[3], theta1 = params.spec.c2de[2], 
            rho1 = params.spec.c2de[1], theta2 = params.spec.c2de[5], rho2 = params.spec.c2de[4], 
            fslow = params.spec.c2de[2]/params.spec.c2de[1]/G0, ffast = params.spec.c2de[5]/params.spec.c2de[4]/G0, 
            fess = params.spec.c2de[3]/G0, Gnew100 = f.coalescent(params.spec.c2de, 
                100)$pan[100] - f.coalescent(params.spec.c2de, 100)$pan[99], Gnew1000 = f.coalescent(params.spec.c2de, 
                1000)$pan[1000] - f.coalescent(params.spec.c2de, 1000)$pan[999], 
            Gcore100 = f.coalescent(params.spec.c2de, 100)$core[100], Gcore1000 = f.coalescent(params.spec.c2de, 
                1000)$core[1000], Gpan100 = f.coalescent(params.spec.c2de, 100)$pan[100], 
            Gpan1000 = f.coalescent(params.spec.c2de, 1000)$pan[1000]))
    })
})

pdf <- collins_results[, 1:10] %>% pivot_longer(cols = colnames(collins_results)[3:10])

ggplot(pdf, aes(x = set, y = value)) + geom_point() + facet_wrap(~name, scales = "free_y")
```

#### Finitely Many Genes model

Here we make use of the implementation of the FMG model as described in Zamani-Dahaj et al., 2016 and implemented in the Panaroo package.

```
imap(sim_bias, function(rep, i) {
    imap(rep, ~{
        ape::write.tree(.x$tree, file = paste(c("./data/sampling_bias/zamani-dahaj/bias_", 
            .y, "_rep_", i, ".tree"), collapse = ""))
        tb <- as_tibble(t(.x$pa)) %>% add_column(gene = colnames(.x$pa), .before = 1)
        write.table(tb, sep = "\t", quote = FALSE, row.names = FALSE, file = paste(c("./data/sampling_bias/zamani-dahaj/bias_", 
            .y, "_rep_", i, "_pa.txt"), collapse = ""))
    })
})
```

```
for f in ./data/sampling_bias/zamani-dahaj/*.tree
do
prefix=$(basename $f)
prefix="${prefix%.*}"
echo $prefix
python ~/Documents/panaroo/panaroo-estimate-fmg.py -o ./data/sampling_bias/zamani-dahaj/${prefix}.txt --pa ./data/sampling_bias/zamani-dahaj/${prefix}_pa.txt --tree $f
done
```

Load results

```
resfiles <- Sys.glob("./data/sampling_bias/zamani-dahaj/bias_*[0-9].txt")

zamani_results <- map_dfr(resfiles, ~{
    df <- fread(.x, skip = 4, header = FALSE, col.names = c("parameter", "estimate", 
        "NA1", "NA2"))[, 1:2] %>% as_tibble() %>% add_column(set = gsub("_rep.*", 
        "", gsub(".*bias_", "", .x)), .before = 1) %>% add_column(rep = gsub("\\.txt", 
        "", gsub(".*rep_", "", .x)), .before = 1)
    return(df)
})

pdf <- zamani_results %>% group_by(set, rep) %>% summarise(parameter = c("u", "v"), 
    estimate = c(estimate[which(parameter == "a")] * estimate[which(parameter == 
        "M")], estimate[which(parameter == "v")]))

ggplot(pdf, aes(x = set, y = estimate)) + geom_point() + facet_wrap(~parameter)
```

#### Accumulation curves (overlapping)

Accumulation curves do not use a statistical model by are very commonly used. Uncertainty is typically quantified using permutations. Here, we consider two curves to be different if there is no overlap in their curves generated by permutation.

```
nperm <- 100
curves <- imap_dfr(sim_bias, function(rep, i) {
    print(i)
    return(imap_dfr(rep, ~{
        .x <- t(.x$pa)
        plotdf <- purrr::map_dfr(1:nperm, function(i) {
            ppa <- .x[sample(nrow(.x), replace = FALSE), sample(ncol(.x), replace = FALSE)]
            cumlative <- rowSums(apply(ppa, 1, cumsum) > 0)
            cumlative <- cumlative - cumlative[[1]]
            df <- tibble::tibble(N = 1:length(cumlative), naccessory = cumlative, 
                permutation = i)
            return(df)
        }) %>% tibble::add_column(pangenome = .y)
        
        plotdf <- plotdf %>% dplyr::group_by(N, pangenome) %>% dplyr::summarise(`accessory size` = mean(naccessory), 
            std = sd(naccessory)) %>% add_column(rep = i, .before = 1) %>% add_column(set = .y, 
            .before = 1)
        return(plotdf)
    }))
})
#> [1] "1"
#> [1] "2"
#> [1] "3"
#> [1] "4"
#> [1] "5"

curves$group <- paste(curves$set, curves$rep, sep = "_")

ggplot(curves, aes(N, `accessory size`, col = set, fill = set, group = group)) + 
    geom_ribbon(aes(ymin = `accessory size` - std, ymax = `accessory size` + std), 
        alpha = 0.5, col = NA) + scale_color_brewer(type = "qual", palette = 5) + 
    scale_fill_brewer(type = "qual", palette = 5) + geom_line(size = 1) + theme_bw(base_size = 14) + 
    xlab("Number of genomes") + ylab("Accessory size") + labs(fill = "error rate", 
    color = "error rate")
```

### Heaps Law approach of Tettlin et al.

```
heap_df <- function(pa) {
    cm <- do.call(rbind, map(1:10, ~{
        ppa <- pa
        ppa <- ppa[sample(nrow(ppa), replace = FALSE), sample(ncol(ppa), replace = FALSE)]
        cumlative <- rowSums(apply(t(ppa), 1, cumsum) > 0)
        cumlative <- cumlative - cumlative[[1]]
        return(cumlative)
    }))
    cumulative_median <- apply(cm, 2, median)
    
    res <- broom::tidy(lm(nunique ~ logN, tibble(logN = log(1:length(cumulative_median)), 
        nunique = log(cumulative_median + 0.001))))
    return(res)
}

heap_results <- imap_dfr(sim_bias, function(rep, i) {
    return(imap_dfr(rep, ~{
        pdf <- heap_df(.x$pa) %>% add_column(rep = i, .before = 1) %>% add_column(set = .y, 
            .before = 1)
    }))
    return(pdf)
})

heap_results$term <- ifelse(heap_results$term == "logN", "alpha", "log(K)")
heap_results <- heap_results %>% filter(term == "alpha")
```

```
ggplot(heap_results, aes(x = set, y = estimate, col = rep)) + geom_point()
```

#### Panstripe

```
pp_results <- imap_dfr(sim_bias, function(rep, i) {
    return(imap_dfr(rep, ~{
        fit <- panstripe(.x$pa, .x$tree, nboot = 0, quiet = TRUE)
        pdf <- fit$summary %>% add_column(rep = i, .before = 1) %>% add_column(set = .y, 
            .before = 1)
        return(pdf)
    }))
})
pp_results <- pp_results %>% filter(term == "core")
```

```
ggplot(pp_results, aes(x = set, y = estimate, col = rep)) + geom_point()
```

#### Summary plot

```
# panicmage
pannic_res <- panicmage_results %>% pivot_longer(cols = colnames(panicmage_results)[3:5]) %>% 
    filter(name == "theta")
pannic_res$value <- c(pannic_res$value)
pannic_test <- t.test(pannic_res$value[pannic_res$set == "full"], pannic_res$value[pannic_res$set == 
    "subset"], alternative = "two.sided")
pannic_test
#> 
#>  Welch Two Sample t-test
#> 
#> data:  pannic_res$value[pannic_res$set == "full"] and pannic_res$value[pannic_res$set == "subset"]
#> t = 18.911, df = 7.8825, p-value = 7.525e-08
#> alternative hypothesis: true difference in means is not equal to 0
#> 95 percent confidence interval:
#>   87.56391 111.95609
#> sample estimates:
#> mean of x mean of y 
#>    119.78     20.02

# collins
collins_res <- collins_results[, 1:10] %>% pivot_longer(cols = colnames(collins_results)[3:10]) %>% 
    filter(name == "theta1")
collins_res$value <- c(collins_res$value)
collins_test <- t.test(collins_res$value[collins_res$set == "full"], collins_res$value[collins_res$set == 
    "subset"], alternative = "two.sided")
collins_test
#> 
#>  Welch Two Sample t-test
#> 
#> data:  collins_res$value[collins_res$set == "full"] and collins_res$value[collins_res$set == "subset"]
#> t = 0.63886, df = 7.4116, p-value = 0.5421
#> alternative hypothesis: true difference in means is not equal to 0
#> 95 percent confidence interval:
#>  -36.13954  63.31167
#> sample estimates:
#> mean of x mean of y 
#>  41.59998  28.01391

# Zamani-Dahaj
zamani_res <- zamani_results %>% filter(parameter == "v")
zamani_res$estimate <- c(zamani_res$estimate)
zamani_test <- t.test(zamani_res$estimate[zamani_res$set == "full"], zamani_res$estimate[zamani_res$set == 
    "subset"], alternative = "two.sided")
zamani_test
#> 
#>  Welch Two Sample t-test
#> 
#> data:  zamani_res$estimate[zamani_res$set == "full"] and zamani_res$estimate[zamani_res$set == "subset"]
#> t = -0.24186, df = 7.3051, p-value = 0.8155
#> alternative hypothesis: true difference in means is not equal to 0
#> 95 percent confidence interval:
#>  -0.0001294222  0.0001052191
#> sample estimates:
#>   mean of x   mean of y 
#> 0.001189721 0.001201822

# accumulation curves
curves_res <- map_dfr(split(curves %>% filter(N > 1), curves$N), ~{
    .x$value = c(.x$`accessory size`)
    return(.x)
})

curves_res <- curves_res %>% group_by(N, rep) %>% summarise(subset = value[set == 
    "subset"], full = value[set == "full"])

curve_test <- t.test(curves_res$subset, curves_res$full, paired = TRUE, alternative = "two.sided")
curve_test
#> 
#>  Paired t-test
#> 
#> data:  curves_res$subset and curves_res$full
#> t = -34.192, df = 292, p-value < 2.2e-16
#> alternative hypothesis: true difference in means is not equal to 0
#> 95 percent confidence interval:
#>  -44.39696 -39.56413
#> sample estimates:
#> mean of the differences 
#>               -41.98055

# Heaps
heap_results$value <- heap_results$estimate
heap_test <- t.test(heap_results$value[heap_results$set == "full"], heap_results$value[heap_results$set == 
    "subset"], alternative = "two.sided")
heap_test
#> 
#>  Welch Two Sample t-test
#> 
#> data:  heap_results$value[heap_results$set == "full"] and heap_results$value[heap_results$set == "subset"]
#> t = -9.9126, df = 4.9617, p-value = 0.0001861
#> alternative hypothesis: true difference in means is not equal to 0
#> 95 percent confidence interval:
#>  -0.5266685 -0.3093622
#> sample estimates:
#> mean of x mean of y 
#> 0.8394112 1.2574265

# panstripe
pp_results$value <- pp_results$estimate
panstripe_test <- t.test(pp_results$value[pp_results$set == "full"], pp_results$value[pp_results$set == 
    "subset"], alternative = "two.sided")
panstripe_test
#> 
#>  Welch Two Sample t-test
#> 
#> data:  pp_results$value[pp_results$set == "full"] and pp_results$value[pp_results$set == "subset"]
#> t = 0.31476, df = 5.3857, p-value = 0.7648
#> alternative hypothesis: true difference in means is not equal to 0
#> 95 percent confidence interval:
#>  -0.5340696  0.6867940
#> sample estimates:
#> mean of x mean of y 
#>  2.044384  1.968022


tests <- tibble(method = c("panicmage", "Zamani-Dahaj", "Collins", "Heaps", "accumulation\ncurve", 
    "panstripe"), t.statistic = c(pannic_test$statistic, zamani_test$statistic, collins_test$statistic, 
    heap_test$statistic, curve_test$statistic, panstripe_test$statistic), p.value = c(pannic_test$p.value, 
    zamani_test$p.value, collins_test$p.value, heap_test$p.value, curve_test$p.value, 
    panstripe_test$p.value))

curves_res_subset <- curves_res %>% filter(N == 20)
pdf <- tibble(values = c(pp_results$value, pannic_res$value, zamani_res$estimate, 
    collins_res$value, heap_results$value, curves_res_subset$subset, curves_res_subset$full), 
    set = c(pp_results$set, pannic_res$set, zamani_res$set, collins_res$set, heap_results$set, 
        rep(c("full", "subset"), each = nrow(curves_res_subset))), method = rep(c("panstripe", 
        "panicmage", "Zamani-Dahaj", "Collins", "Heaps", "accumulation\ncurve"), 
        c(nrow(pp_results), nrow(pannic_res), nrow(zamani_res), nrow(collins_res), 
            nrow(heap_results), 2 * nrow(curves_res_subset))))

pdf$method <- factor(pdf$method, levels = c("panstripe", "Zamani-Dahaj", "Collins", 
    "panicmage", "Heaps", "accumulation\ncurve"))


pdf$is.sig <- ifelse(pdf$method %in% tests$method[tests$p.value < 0.05], "significant", 
    "not significant")

ggplot(pdf, aes(x = set, y = values, col = is.sig)) + geom_boxplot(outlier.colour = NA, 
    position = position_dodge(width = 0.8)) + geom_point(size = 2, position = position_dodge(width = 0.8)) + 
    theme_clean(base_size = 20) + facet_wrap(~method, scales = "free", nrow = 1) + 
    scale_color_manual(values = c("#4d4d4d", "#b2182b")) + theme(plot.background = element_blank(), 
    legend.background = element_blank(), axis.title.x = element_blank(), panel.spacing = unit(2, 
        "lines")) + # scale_alpha_discrete(guide = 'none') +
ylab("estimated parameter value (scaled)") + labs(color = "")
```

```
ggsave("./figures/simulation_sampling_bias_summary.png", width = 17, height = 7)
ggsave("./figures/simulation_sampling_bias_summary.pdf", width = 17, height = 7)
```
