# Supplementary material for: Robust analysis of prokaryotic pangenome gene gain and loss rates with Panstripe
Source: Genome Res. 2023 Jan;33(1):129–40. doi: 10.1101/gr.277340.122 (PMC9977150; doi:10.1101/gr.277340.122)
Supplement: Supplemental Material [file supp_gr.277340.122_Supplemental_Code_0.1.0.tar.gz.zip › panstripe-manuscript-0.1.0/spneumo.html]

S. pneumoniae


# S. pneumoniae

```
library(panstripe)
library(tidyverse)
library(data.table)
library(ape)
library(ggthemes)
library(ggtree)
library(ggtreeExtra)
```

## Load data

```
rtab_files <- Sys.glob("./data/spneumoniae/*.Rtab")
pas <- map(rtab_files, read_rtab)
names(pas) <- gsub("_strict.*", "", gsub(".*/", "", rtab_files))

tree_files <- Sys.glob("./data/spneumoniae/*.tre")
trees <- map(tree_files, read.tree)
names(trees) <- gsub("_BD.*", "", gsub(".*GPSC", "", tree_files))
trees <- trees[names(pas)]

or_data <- fread("./data/spneumoniae/GPSC_OR.csv") %>% as_tibble()
```

## Plot trees

```
patchwork::wrap_plots(imap(trees, ~ggtree(.x) + ggtree::theme_tree2() + ggtitle(paste("GPSC", 
    .y))))
```

## Run panstripe

```
fits <- map2(pas, trees, ~panstripe(.x, .y, quiet = FALSE, nboot = 0))
saveRDS(fits, file = "./data/spneumo_fits.RDS")
```

## Plot results

```
or_data$invasive <- (or_data$pvalue < 0.05) & (log(or_data$logOR) > 0)
or_data$GPSC <- as.character(or_data$GPSC)
lineage_size <- map_dbl(fits, ~nrow(.x$pa))
or_data_subset <- or_data[or_data$GPSC %in% names(lineage_size)[lineage_size >= 0], 
    ]
# or_data_subset <- or_data_subset[or_data_subset$pvalue<0.1,]
fit_subset <- fits[names(fits) %in% or_data_subset$GPSC]
comp <- compare_pangenome_covariates(fit_subset, or_data_subset, keep = "invasive", 
    nboot = 100)
comp$summary
#> # A tibble: 4 × 7
#>   term   estimate std.error statistic  p.value `bootstrap CI …` `bootstrap CI …`
#>   <chr>     <dbl>     <dbl>     <dbl>    <dbl>            <dbl>            <dbl>
#> 1 depth… -1.57e-3  0.000162     -9.69 3.77e-22         -0.00199         -0.00117
#> 2 istip… -1.29e-1  0.0439       -2.92 3.45e- 3         -0.225            0.00610
#> 3 core:… -9.99e-4  0.000451     -2.21 2.68e- 2         -0.00552          0.00313
#> 4 istip… -3.54e-3  0.000625     -5.66 1.50e- 8         -0.0142           0.00297
```

```
pdf <- comp$model$model %>% as_tibble()
pdf$invasive <- ifelse(pdf$invasive, "Invasive", "Not invasive")

ggplot(pdf %>% filter(istip), aes(x = invasive, y = acc, col = invasive)) + ggbeeswarm::geom_quasirandom(alpha = 0.2, 
    width = 0.2) + geom_boxplot(outlier.colour = NA, fill = NA, col = "#4d4d4d") + 
    scale_colour_manual(values = c("#e41a1c", "#377eb8")) + theme_clean(base_size = 20) + 
    theme(plot.background = element_blank(), legend.background = element_blank(), 
        legend.position = "none") + theme(panel.border = element_blank(), axis.line = element_line()) + 
    ylab("accessory gene gain/loss events at tip") + xlab("")
```

```
ggsave("./figures/pneumo_tip_acc_count_histogram.pdf", width = 10, height = 7)
ggsave("./figures/pneumo_tip_acc_count_histogram.png", width = 10, height = 7)


pdf <- tibble(core_estimate = map_dbl(fit_subset, ~.x$summary$estimate[.x$summary$term == 
    "core"]), invasive = ifelse(or_data_subset$invasive[match(names(fit_subset), 
    or_data_subset$GPSC)], "Invasive", "Not Invasive"))

ggplot(pdf, aes(x = invasive, y = core_estimate, colour = invasive)) + geom_boxplot(outlier.color = NA, 
    fill = NA) + ggbeeswarm::geom_quasirandom(width = 0.2, size = 2) + scale_colour_manual(values = c("#e41a1c", 
    "#377eb8")) + theme_clean(base_size = 20) + theme(plot.background = element_blank(), 
    legend.background = element_blank(), legend.position = "none") + theme(panel.border = element_blank(), 
    axis.line = element_line()) + ylab("Estimated core coefficient") + xlab("")
```

```
ggsave("./figures/pneumo_core_estimate_boxplot.pdf", width = 10, height = 7)
ggsave("./figures/pneumo_core_estimate_boxplot.png", width = 10, height = 7)
```

```
dist_df <- imap_dfr(trees, ~{
    d <- cophenetic.phylo(.x)
    d[lower.tri(d, diag = TRUE)] <- NA
    d <- matrixStats::colMins(d, na.rm = TRUE)
    tibble(GPSC = .y, distance = d)
})

dist_df <- dist_df[dist_df$GPSC %in% or_data_subset$GPSC, ]
dist_df$invasive <- or_data_subset$invasive[match(dist_df$GPSC, or_data_subset$GPSC)]
dist_df$invasive <- ifelse(dist_df$invasive, "Invasive", "Not invasive")

ggplot(dist_df %>% filter(distance < 50), aes(x = distance, fill = invasive)) + geom_histogram() + 
    facet_wrap(~invasive, ncol = 1, scales = "free_y") + scale_fill_manual(values = c("#e41a1c", 
    "#377eb8")) + theme_clean(base_size = 20) + theme(plot.background = element_blank(), 
    legend.background = element_blank(), legend.position = "none") + theme(panel.border = element_blank(), 
    axis.line = element_line()) + xlab("distance to nearest neighbour in phylogeny (years)")
```

```
ggsave("./figures/GPSC_nn_histogram.pdf", width = 12, height = 7)
ggsave("./figures/GPSC_nn_histogram.png", width = 12, height = 7)
```

```
trees_sig <- trees[names(trees) %in% or_data_subset$GPSC]

ggs <- imap(trees_sig, ~{
    colour <- ifelse(.y %in% or_data_subset$GPSC[or_data_subset$invasive], "#e41a1c", 
        "#377eb8")
    gg <- ggtree(.x, col = colour) + ggtree::theme_tree2() + xlim_tree(xlim = 500) + 
        ggtitle(paste("GPSC", .y))
    return(gg)
})

patchwork::wrap_plots(ggs)
```

```
ggsave("./figures/GPSC_phylogenies_sigOR.pdf", width = 12, height = 10)
ggsave("./figures/GPSC_phylogenies_sigOR.png", width = 12, height = 10)
```
