# Supplementary figures and images for: Robust analysis of prokaryotic pangenome gene gain and loss rates with Panstripe
Source: Genome Res. 2023 Jan;33(1):129–40. doi: 10.1101/gr.277340.122 (PMC9977150; doi:10.1101/gr.277340.122)

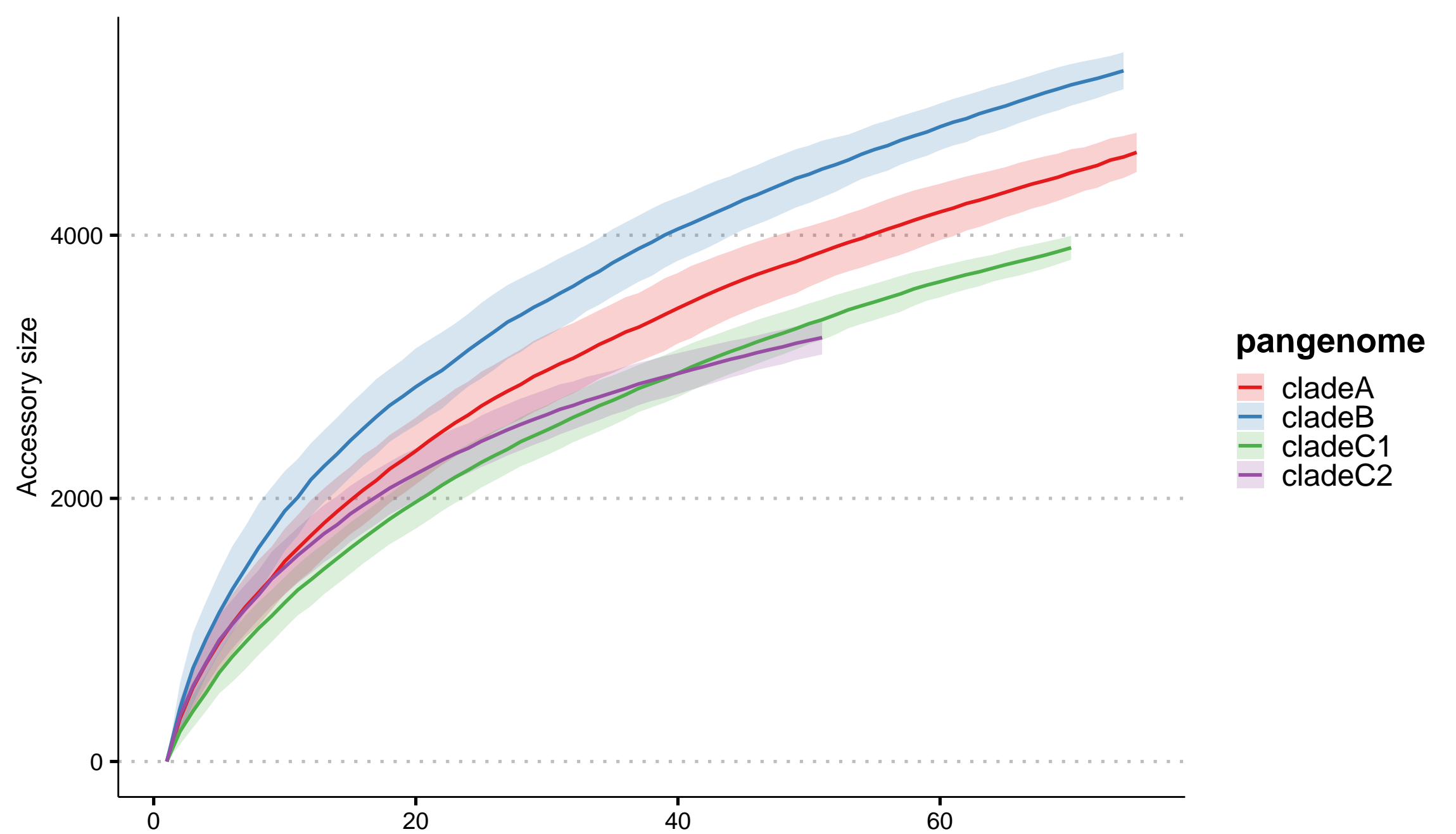

Supplement: Supplemental Material [file supp_gr.277340.122_Supplemental_Code_0.1.0.tar.gz.zip › panstripe-manuscript-0.1.0/figures/ecoli_accumulation_curves.pdf]

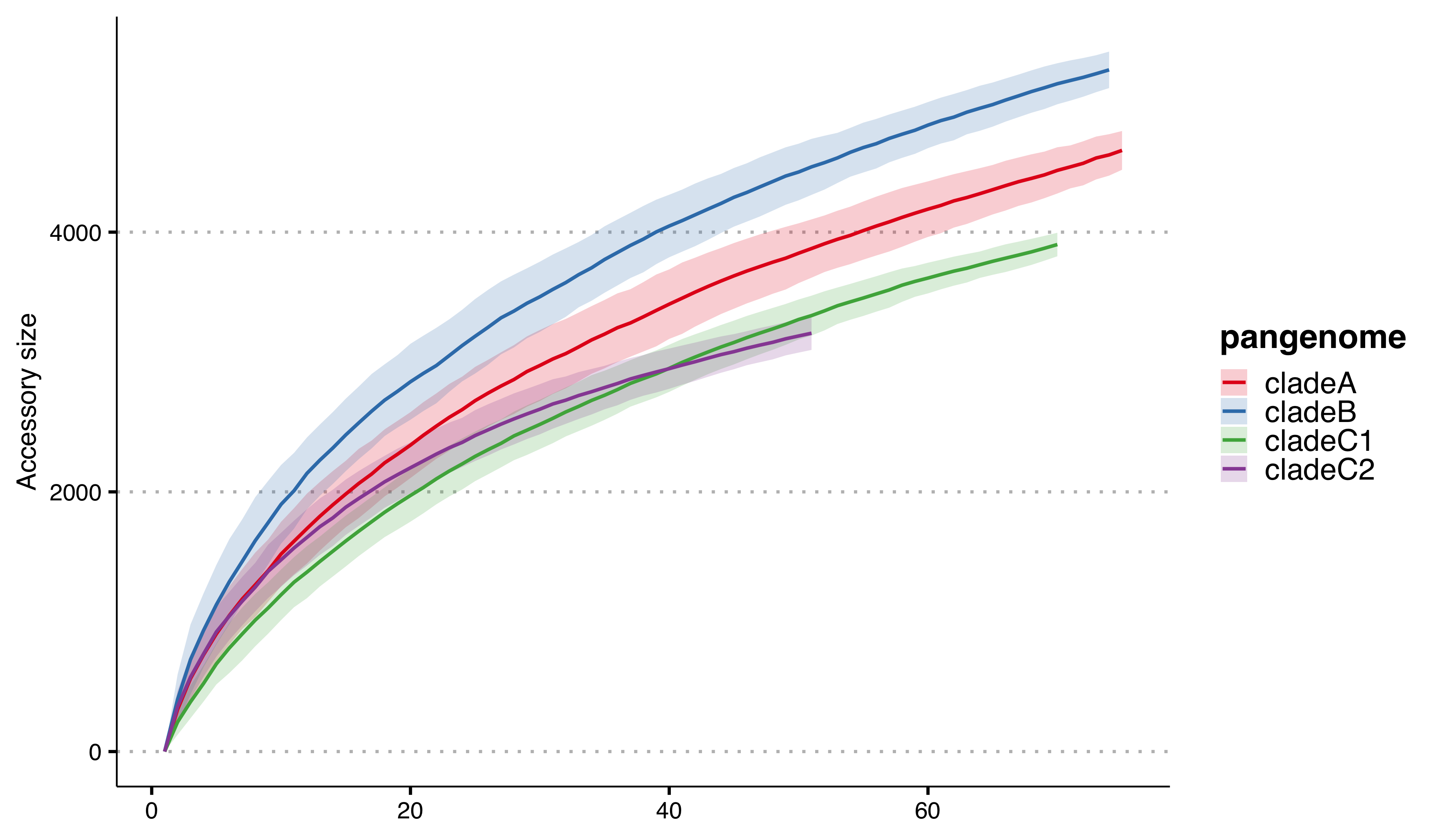

Supplement: Supplemental Material [file supp_gr.277340.122_Supplemental_Code_0.1.0.tar.gz.zip › panstripe-manuscript-0.1.0/figures/ecoli_accumulation_curves.png]

A.

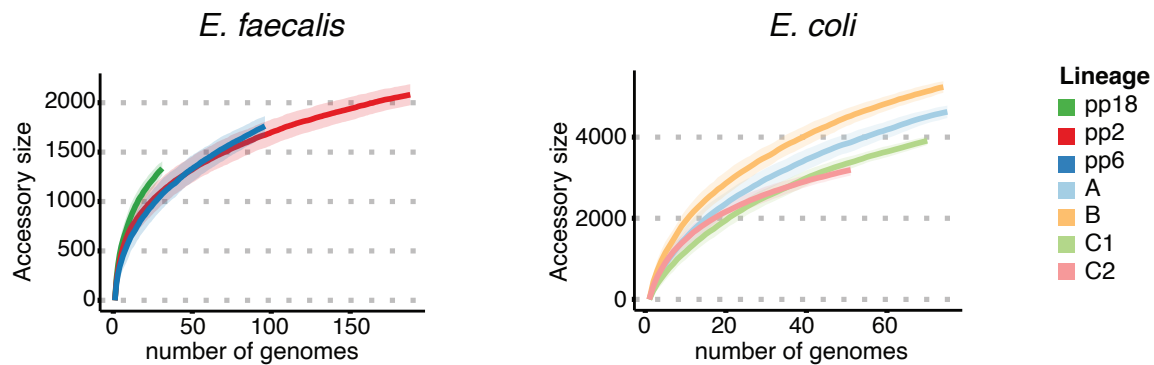

B.

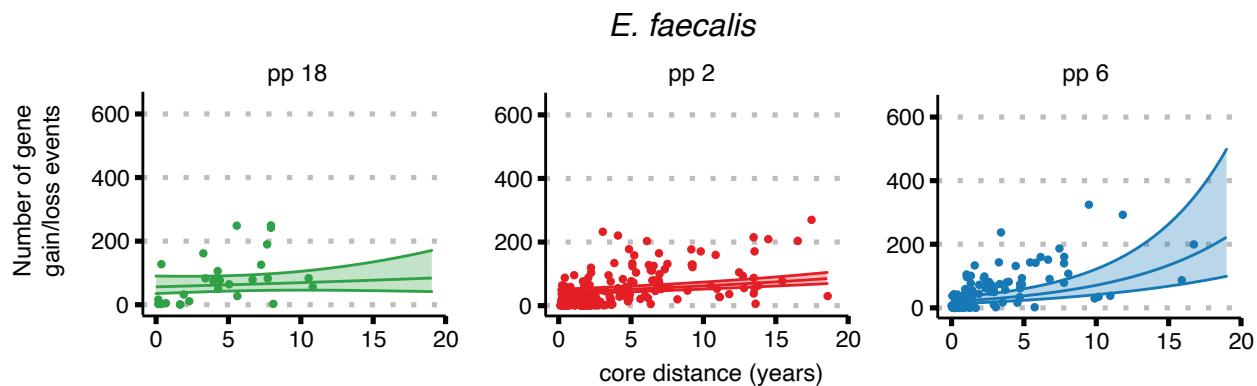

C.

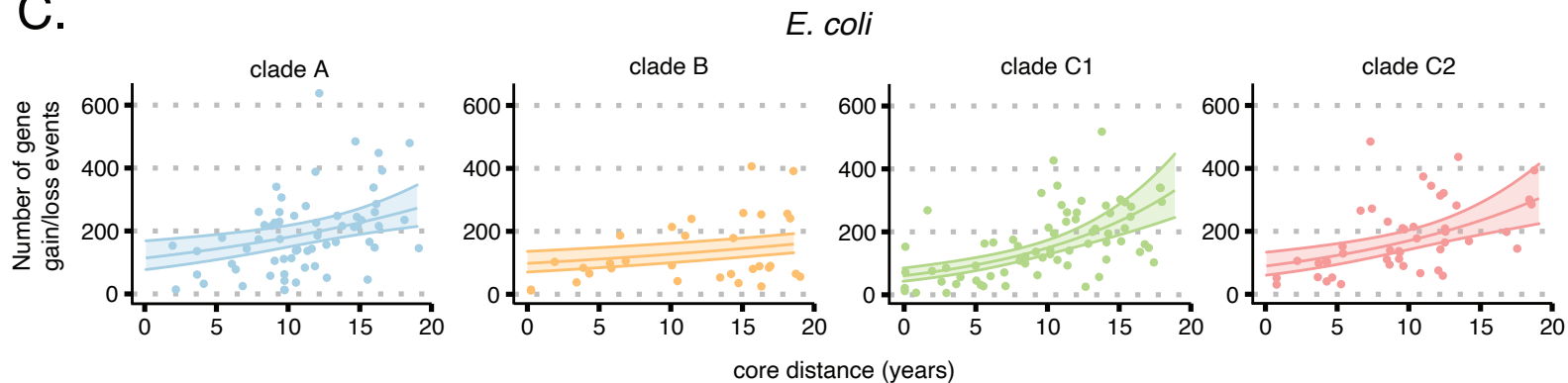

Supplement: Supplemental Material [file supp_gr.277340.122_Supplemental_Code_0.1.0.tar.gz.zip › panstripe-manuscript-0.1.0/figures/ecoli_efaecalis.pdf]

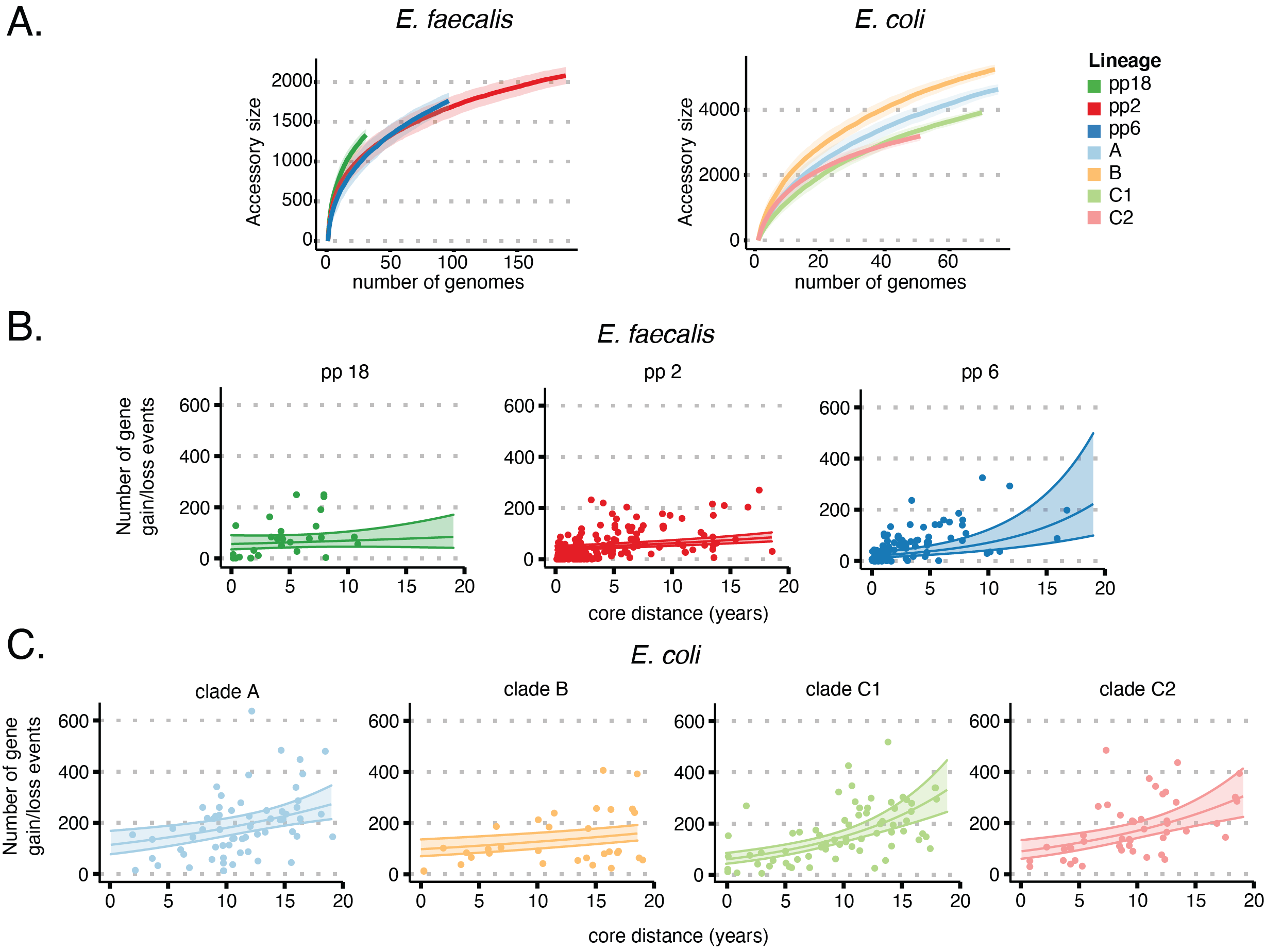

Supplement: Supplemental Material [file supp_gr.277340.122_Supplemental_Code_0.1.0.tar.gz.zip › panstripe-manuscript-0.1.0/figures/ecoli_efaecalis.png]

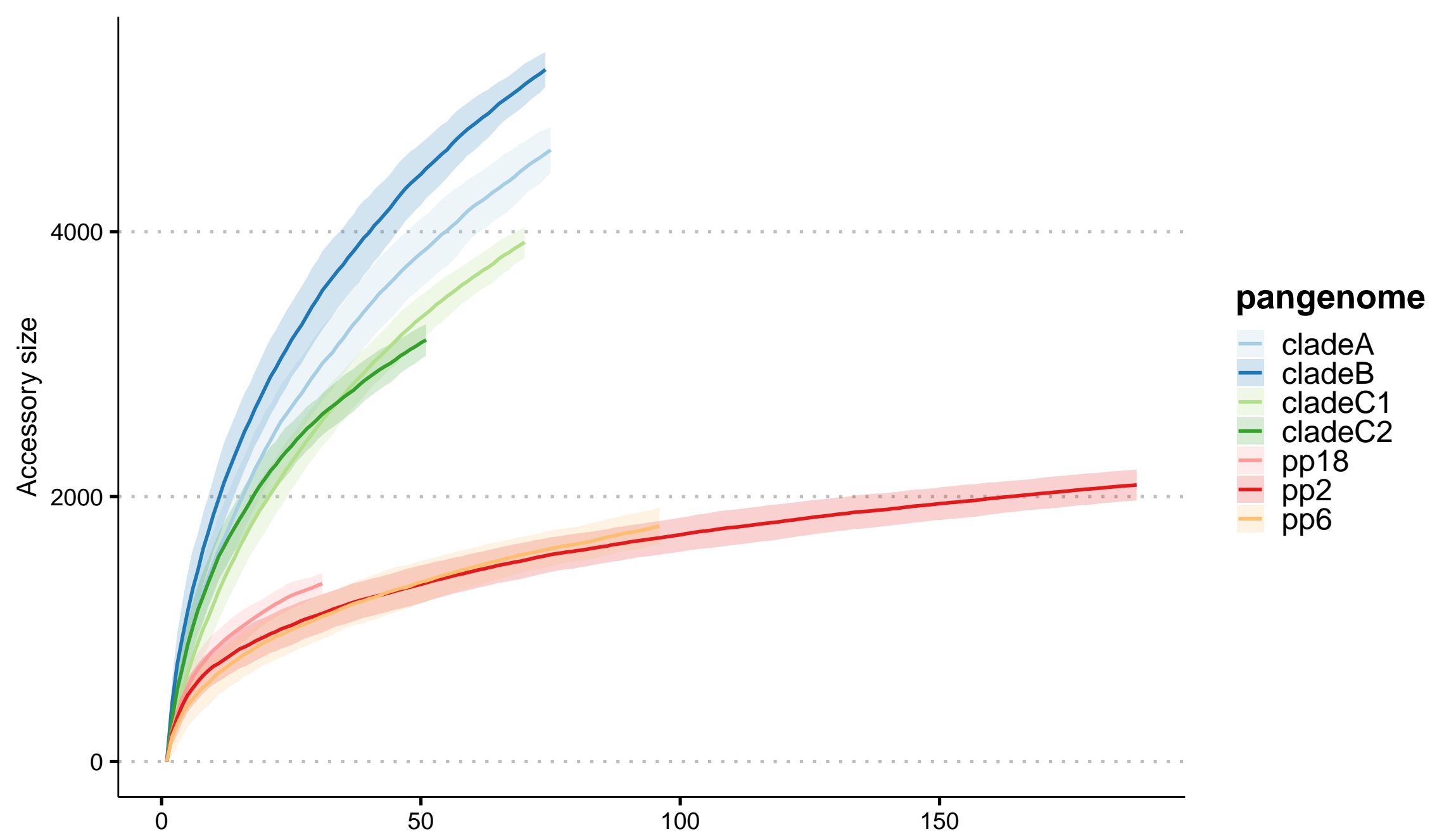

Supplement: Supplemental Material [file supp_gr.277340.122_Supplemental_Code_0.1.0.tar.gz.zip › panstripe-manuscript-0.1.0/figures/ecoli_efaecalis_accumulation_curves.pdf]

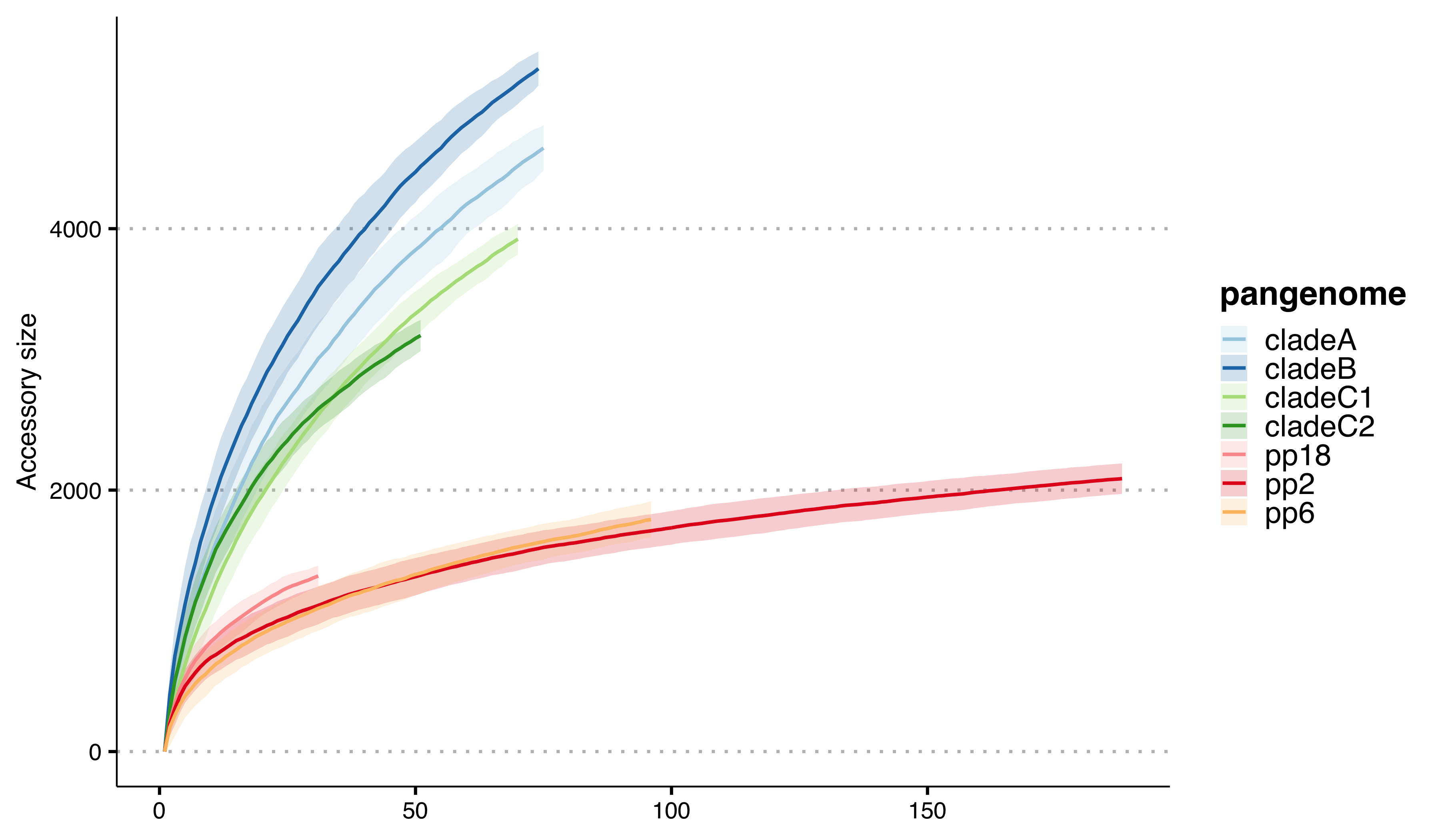

Supplement: Supplemental Material [file supp_gr.277340.122_Supplemental_Code_0.1.0.tar.gz.zip › panstripe-manuscript-0.1.0/figures/ecoli_efaecalis_accumulation_curves.png]

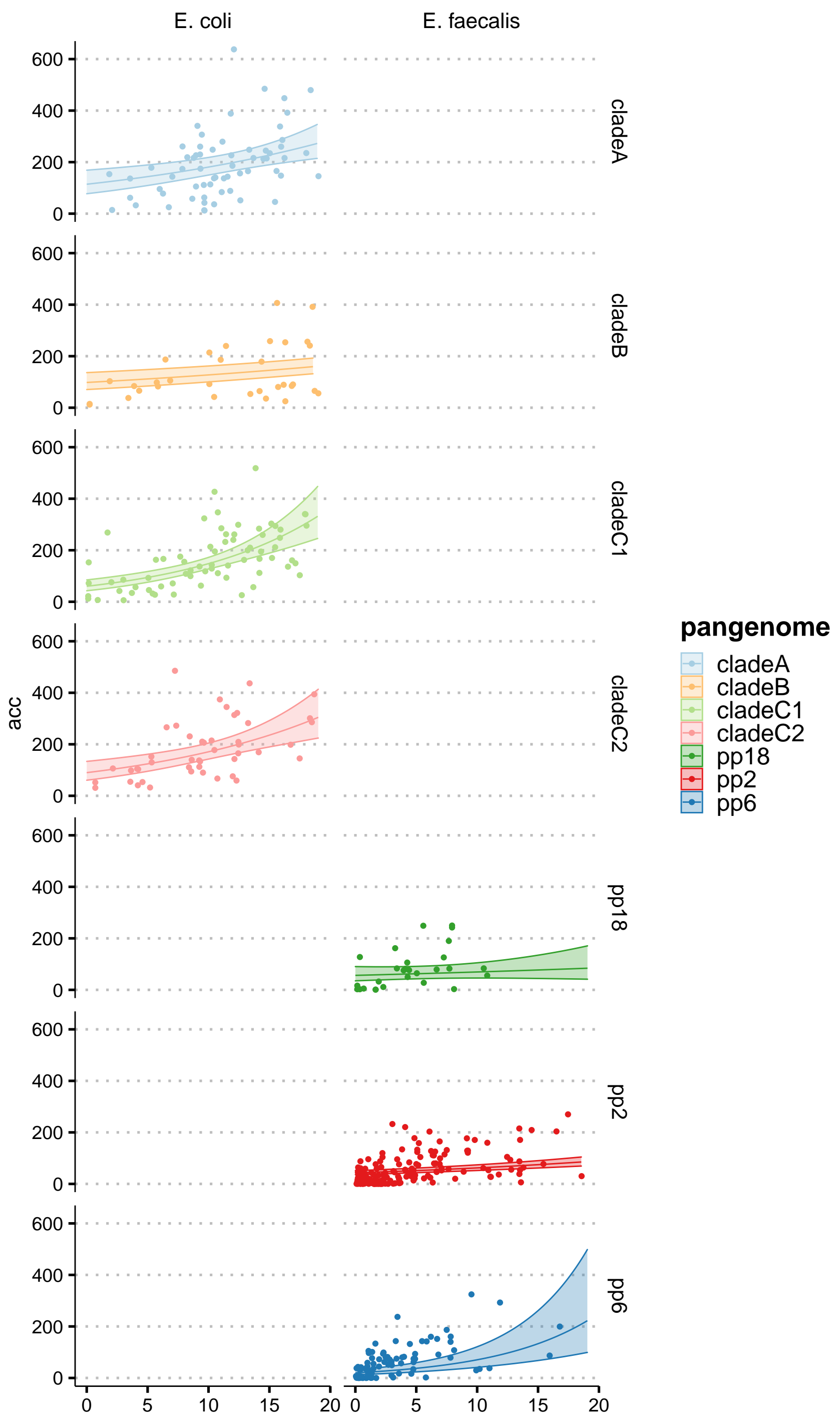

Supplement: Supplemental Material [file supp_gr.277340.122_Supplemental_Code_0.1.0.tar.gz.zip › panstripe-manuscript-0.1.0/figures/ecoli_efaecalis_pangenome_fits.pdf]

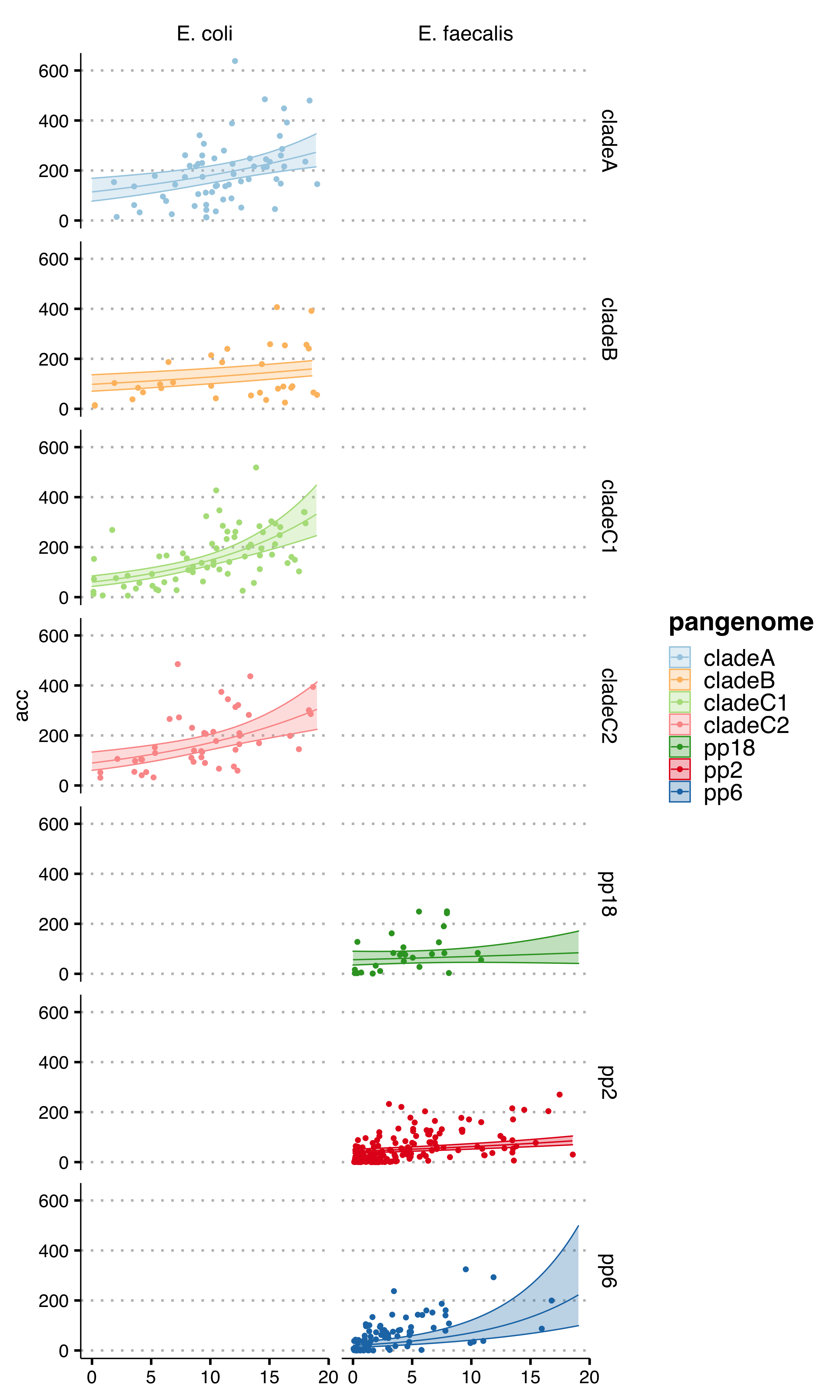

Supplement: Supplemental Material [file supp_gr.277340.122_Supplemental_Code_0.1.0.tar.gz.zip › panstripe-manuscript-0.1.0/figures/ecoli_efaecalis_pangenome_fits.png]

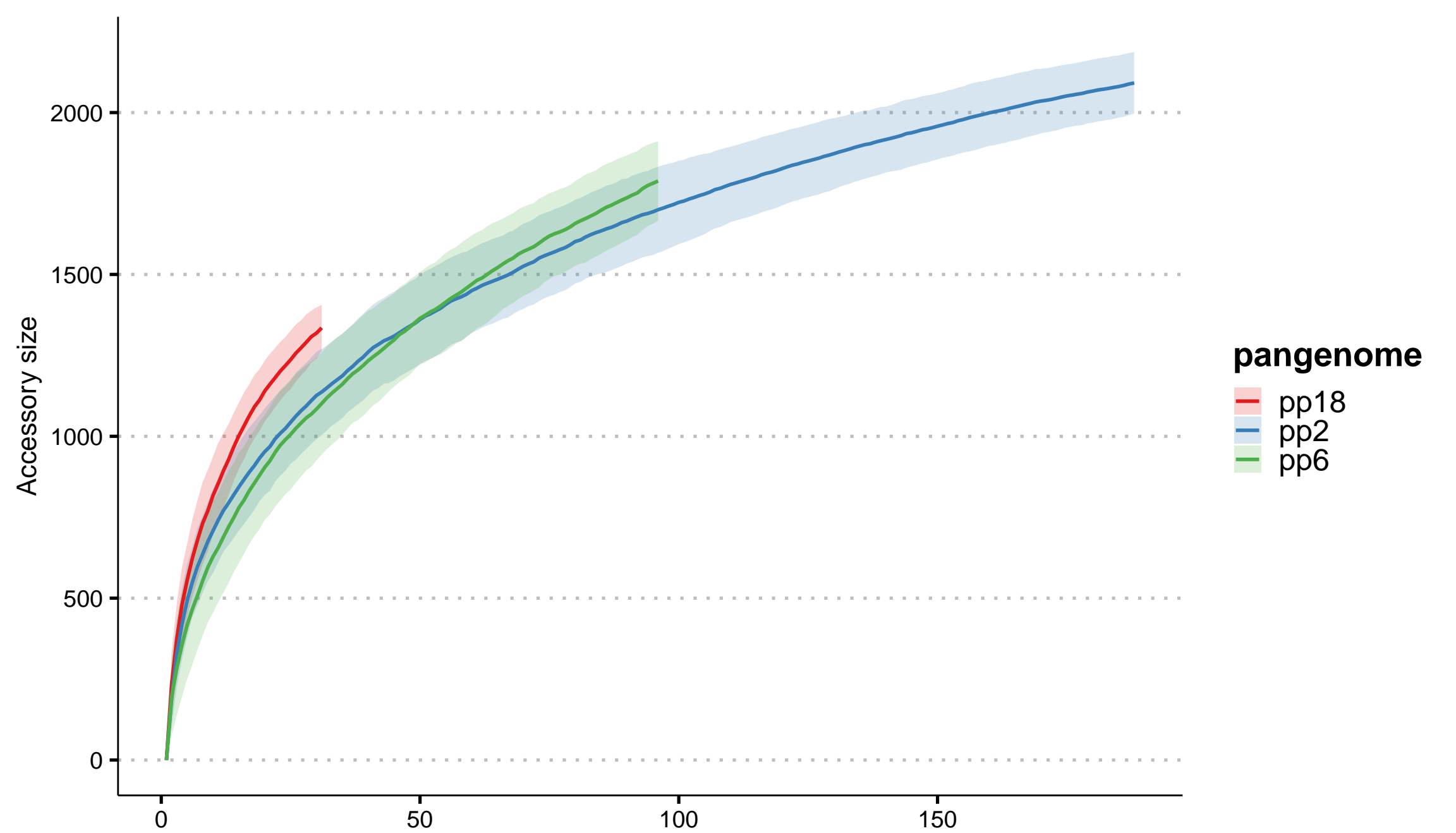

Supplement: Supplemental Material [file supp_gr.277340.122_Supplemental_Code_0.1.0.tar.gz.zip › panstripe-manuscript-0.1.0/figures/efaecalis_pangenome_accumulation_curves.pdf]

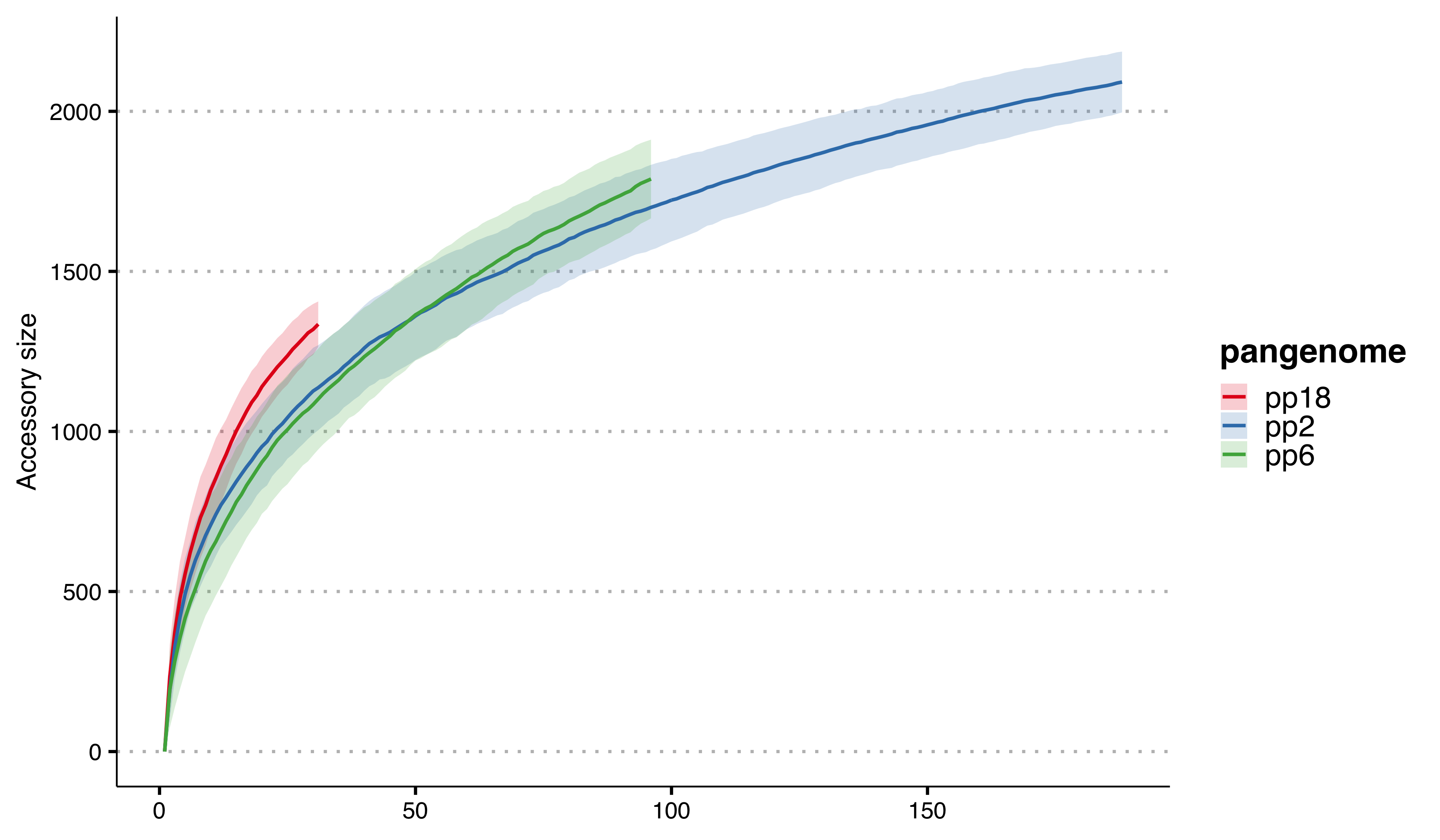

Supplement: Supplemental Material [file supp_gr.277340.122_Supplemental_Code_0.1.0.tar.gz.zip › panstripe-manuscript-0.1.0/figures/efaecalis_pangenome_accumulation_curves.png]

pp18

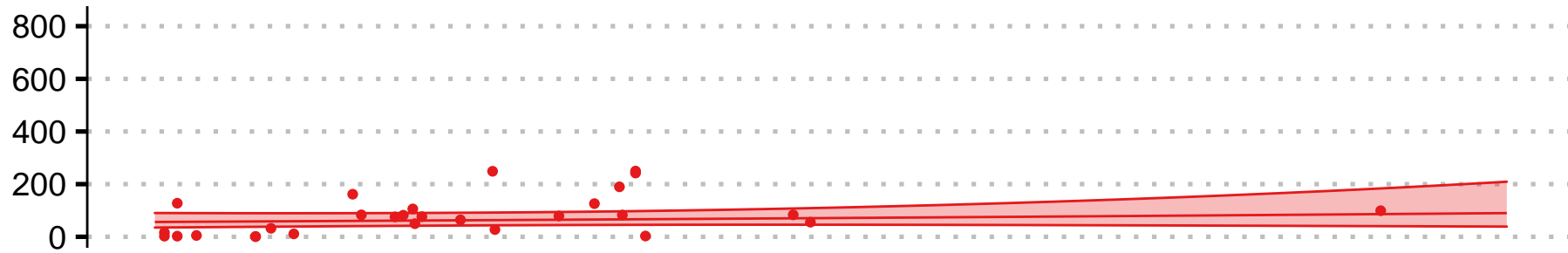

pp2

accessory distance

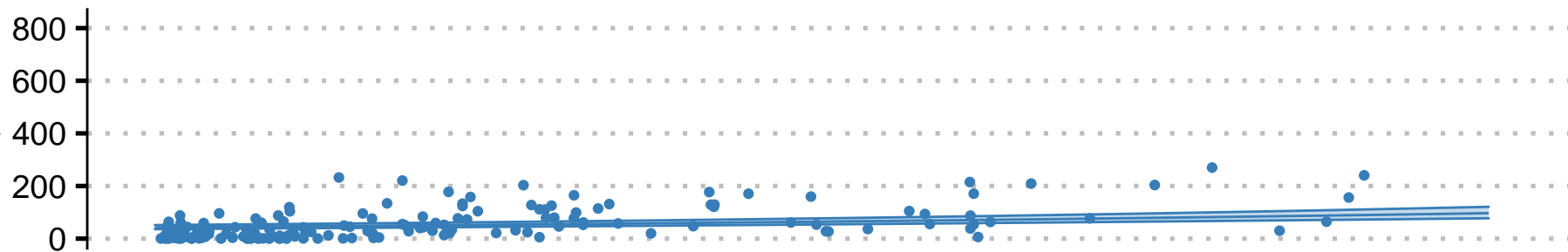**pangenome**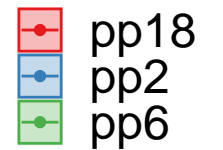

pp6

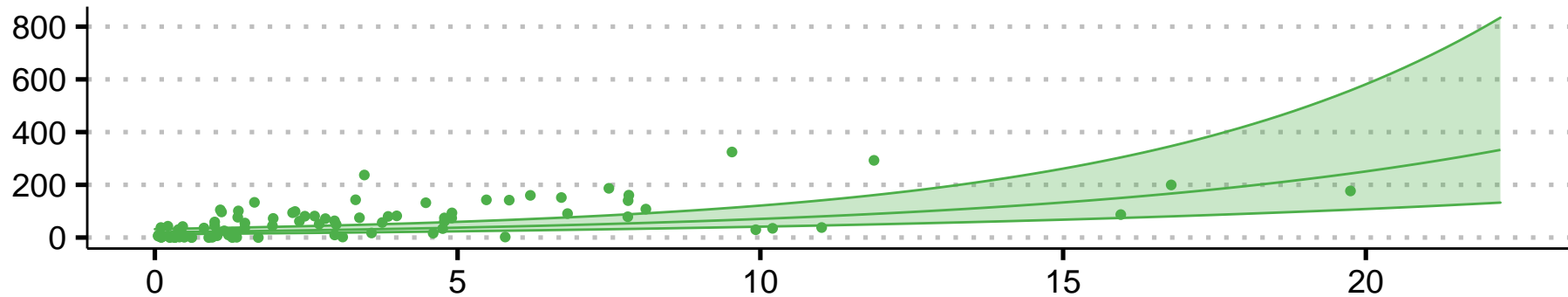

Supplement: Supplemental Material [file supp_gr.277340.122_Supplemental_Code_0.1.0.tar.gz.zip › panstripe-manuscript-0.1.0/figures/efaecalis_pangenome_fits.pdf]

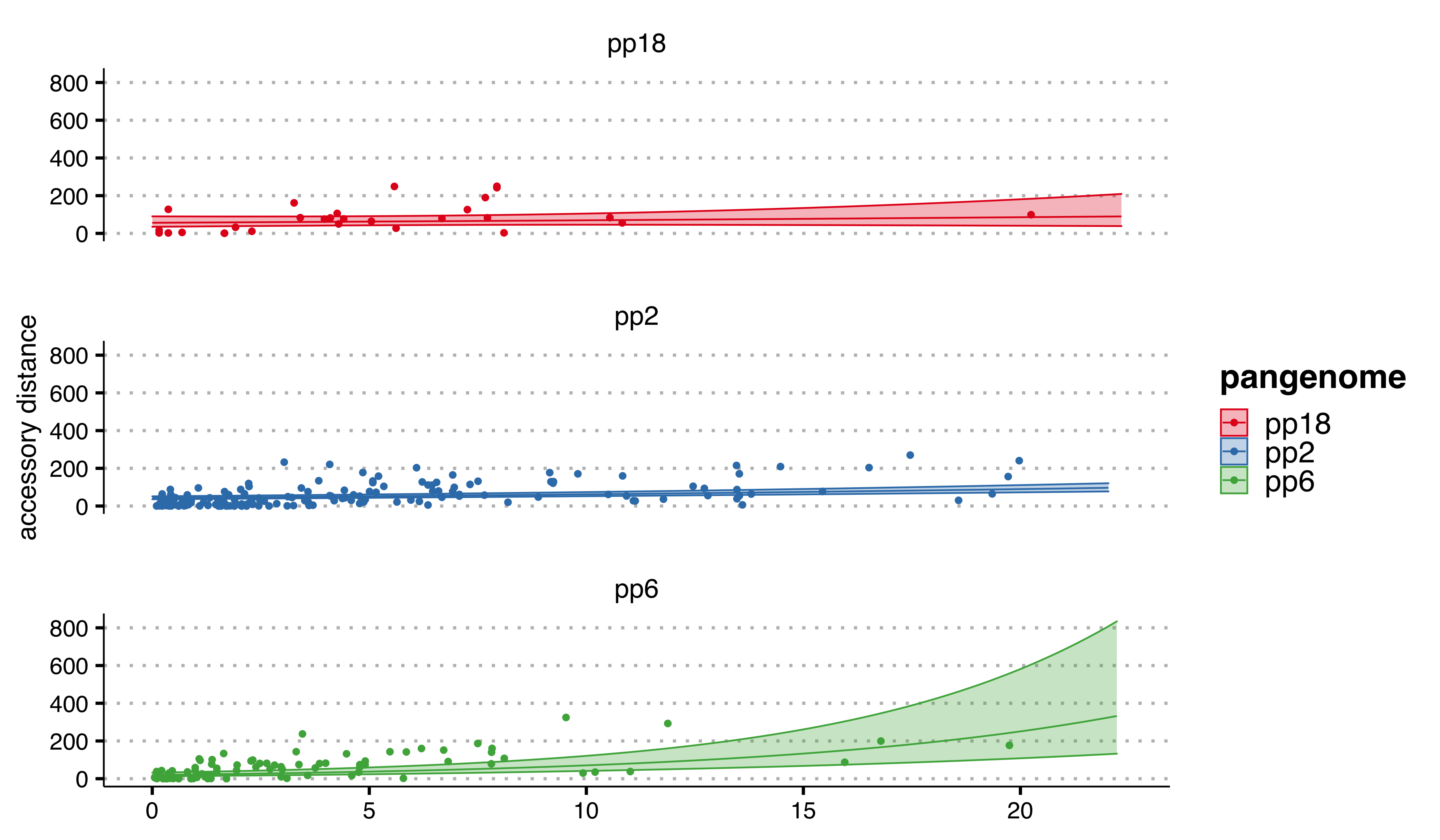

Supplement: Supplemental Material [file supp_gr.277340.122_Supplemental_Code_0.1.0.tar.gz.zip › panstripe-manuscript-0.1.0/figures/efaecalis_pangenome_fits.png]

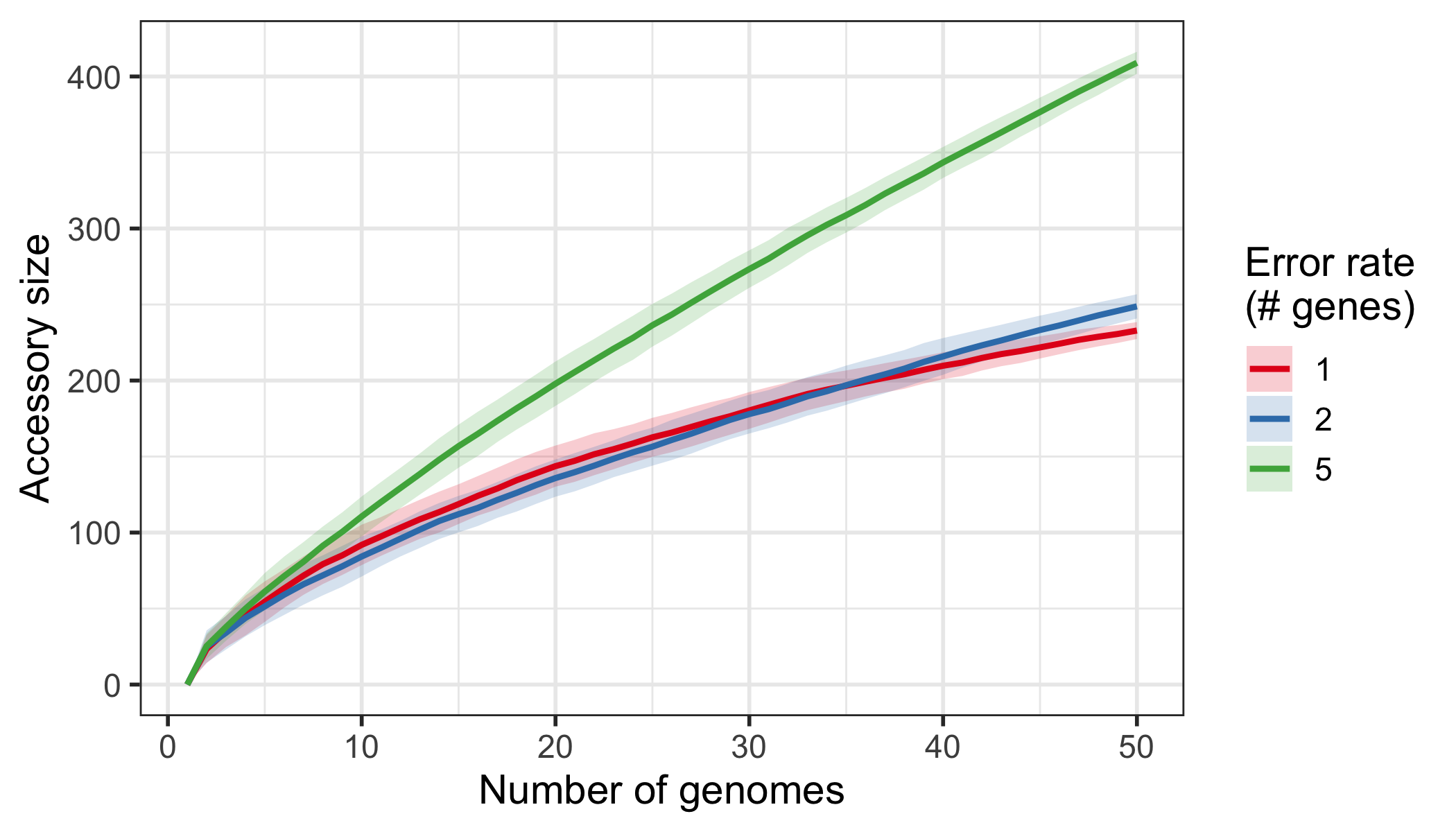

Supplement: Supplemental Material [file supp_gr.277340.122_Supplemental_Code_0.1.0.tar.gz.zip › panstripe-manuscript-0.1.0/figures/error_acc.png]

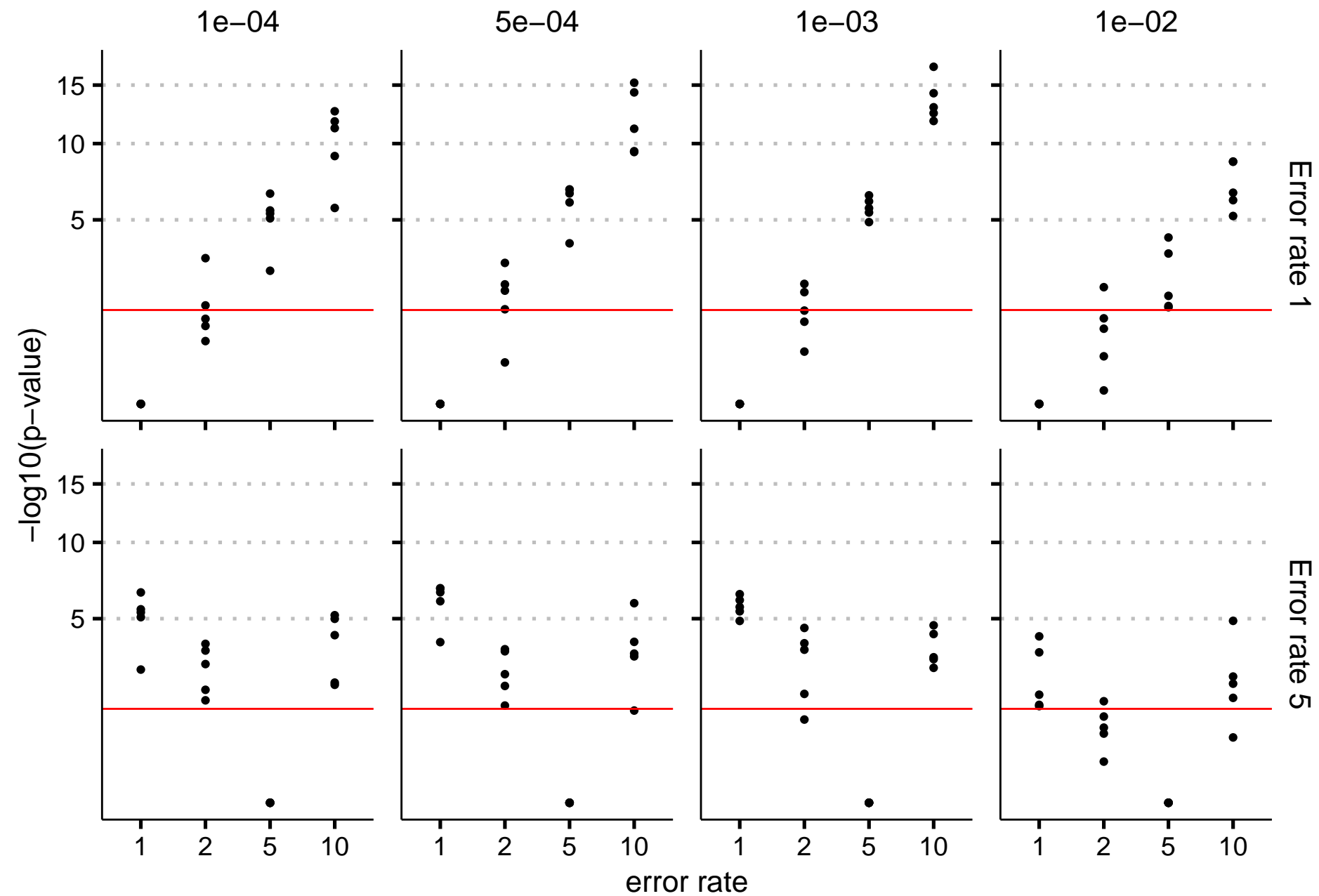

Supplement: Supplemental Material [file supp_gr.277340.122_Supplemental_Code_0.1.0.tar.gz.zip › panstripe-manuscript-0.1.0/figures/error_rate_comparison.pdf]

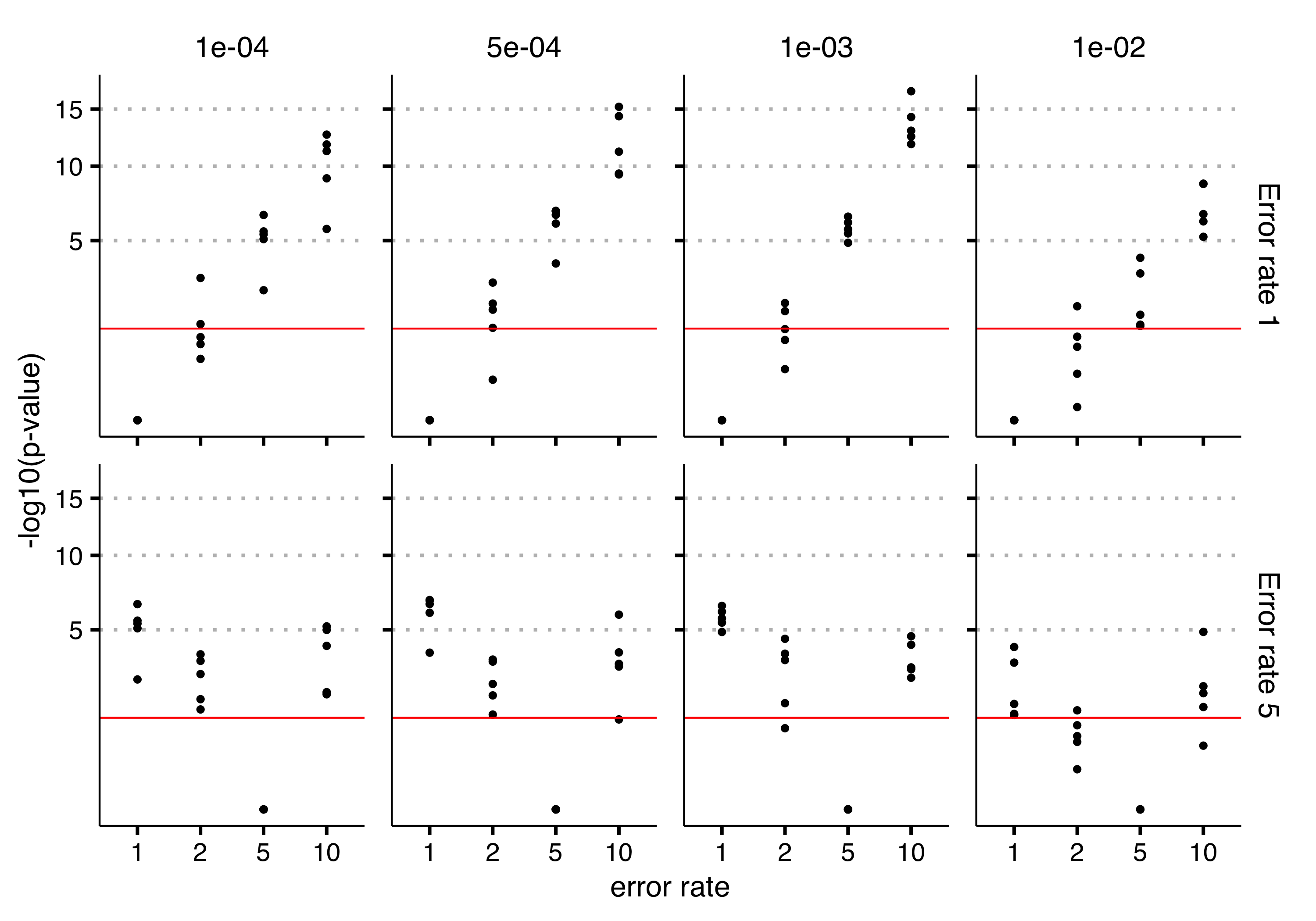

Supplement: Supplemental Material [file supp_gr.277340.122_Supplemental_Code_0.1.0.tar.gz.zip › panstripe-manuscript-0.1.0/figures/error_rate_comparison.png]

**A.**

Accumulation curve

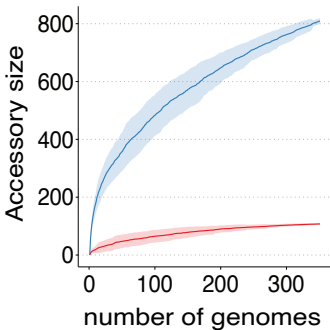**B.**

Panstripe

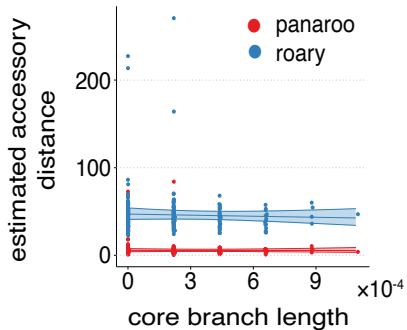

Supplement: Supplemental Material [file supp_gr.277340.122_Supplemental_Code_0.1.0.tar.gz.zip › panstripe-manuscript-0.1.0/figures/fig2.pdf]

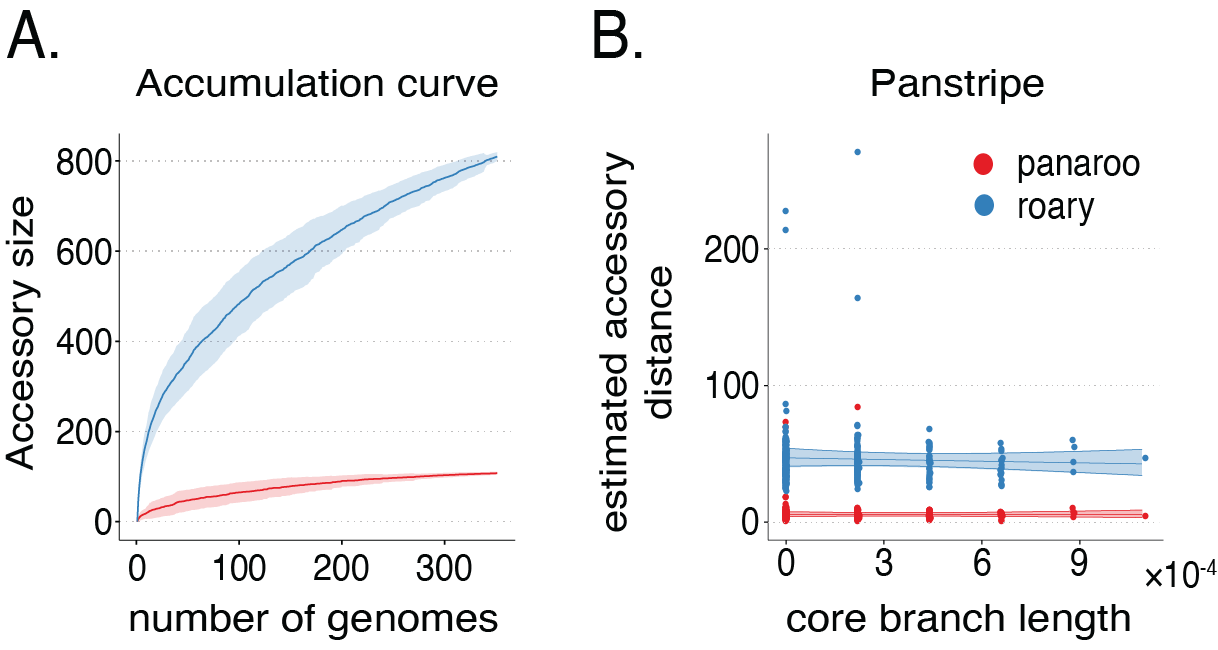

Supplement: Supplemental Material [file supp_gr.277340.122_Supplemental_Code_0.1.0.tar.gz.zip › panstripe-manuscript-0.1.0/figures/fig2.png]

**A.**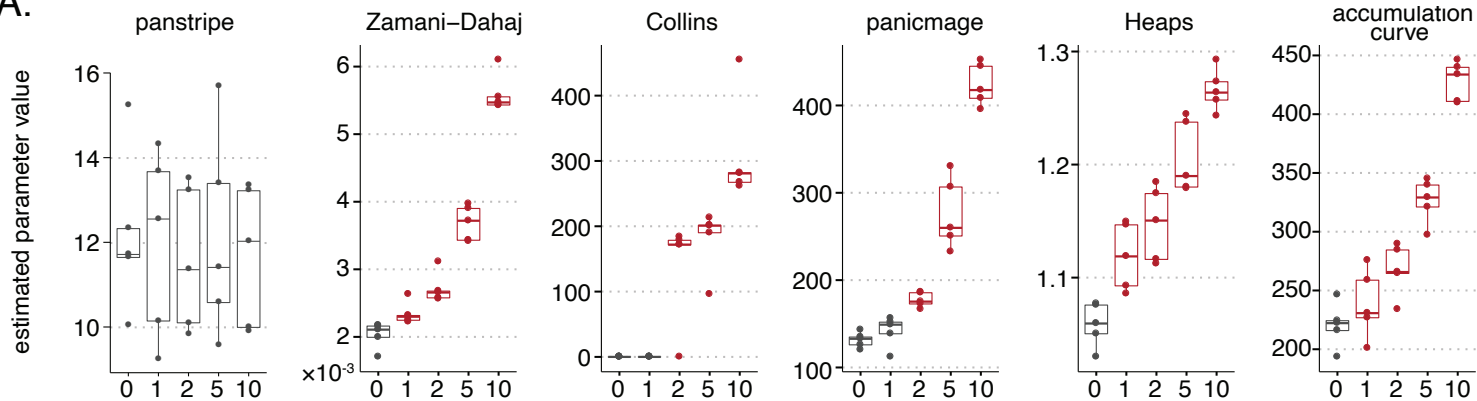**B.**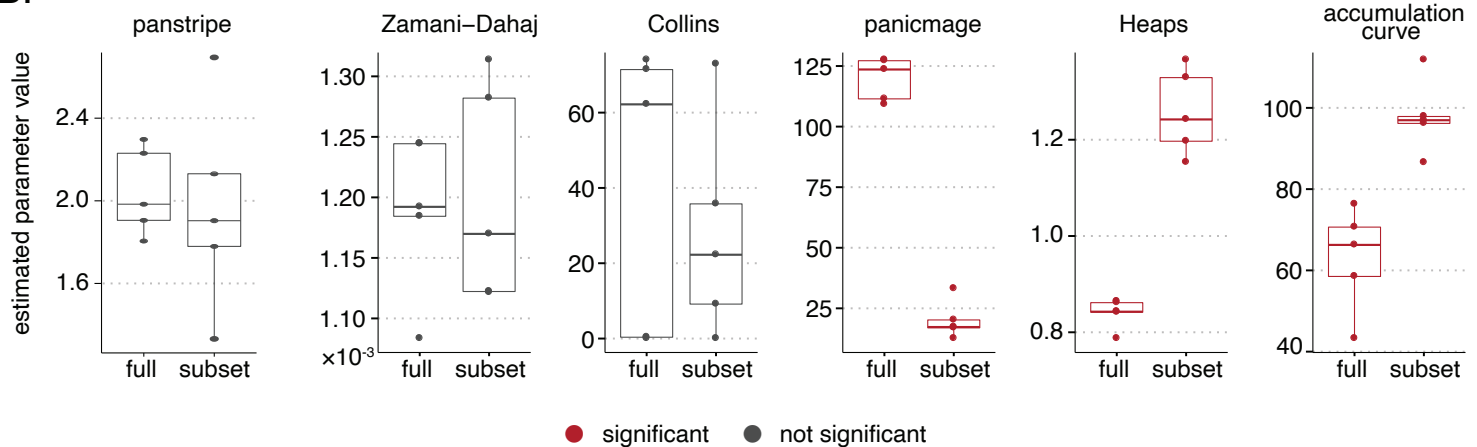

Supplement: Supplemental Material [file supp_gr.277340.122_Supplemental_Code_0.1.0.tar.gz.zip › panstripe-manuscript-0.1.0/figures/fig3.pdf]

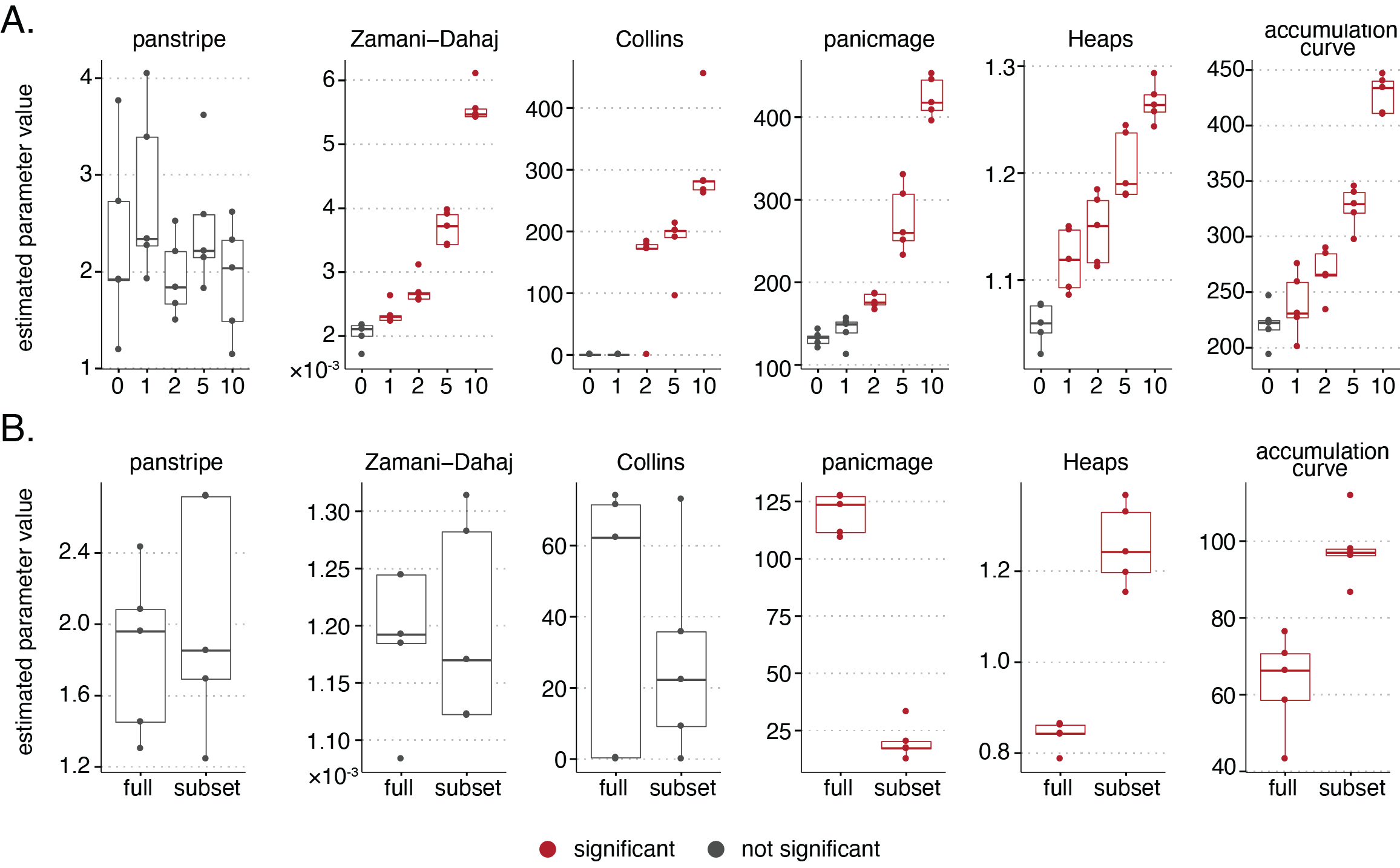

Supplement: Supplemental Material [file supp_gr.277340.122_Supplemental_Code_0.1.0.tar.gz.zip › panstripe-manuscript-0.1.0/figures/fig3.png]

A.

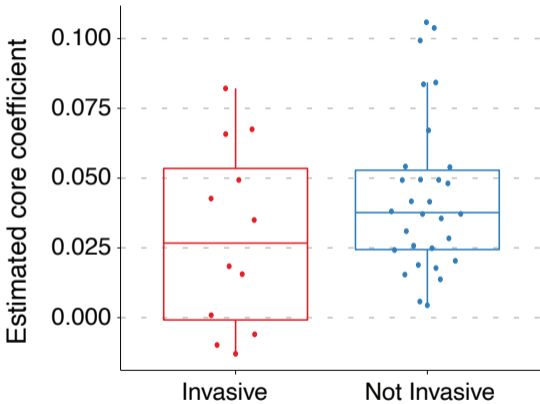

B.

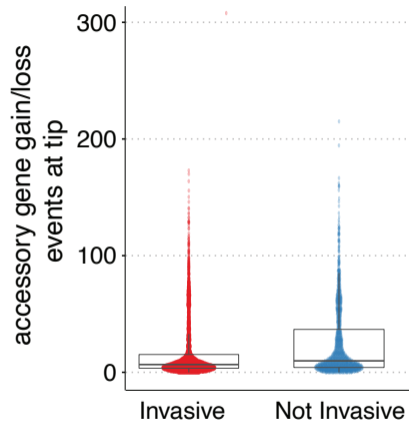

C.

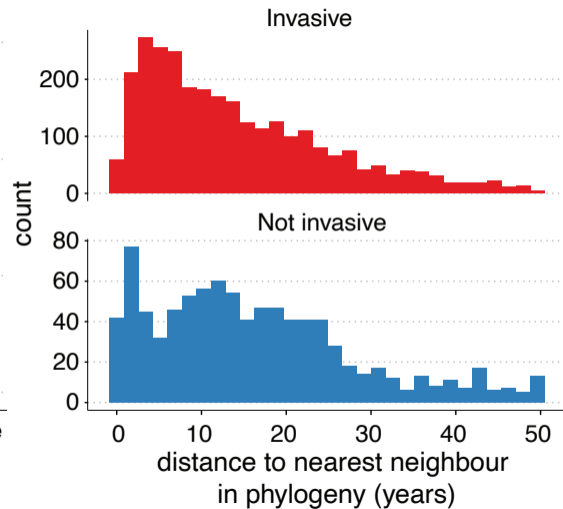

Supplement: Supplemental Material [file supp_gr.277340.122_Supplemental_Code_0.1.0.tar.gz.zip › panstripe-manuscript-0.1.0/figures/fig4.pdf]

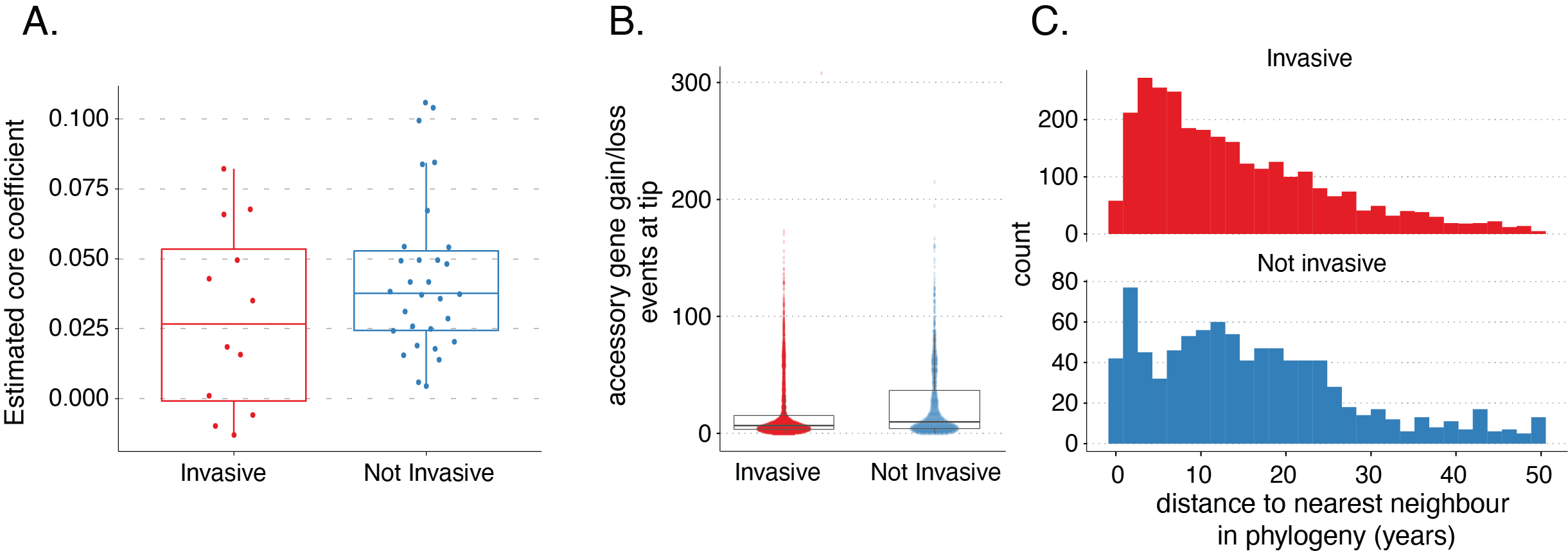

Supplement: Supplemental Material [file supp_gr.277340.122_Supplemental_Code_0.1.0.tar.gz.zip › panstripe-manuscript-0.1.0/figures/fig4.png]

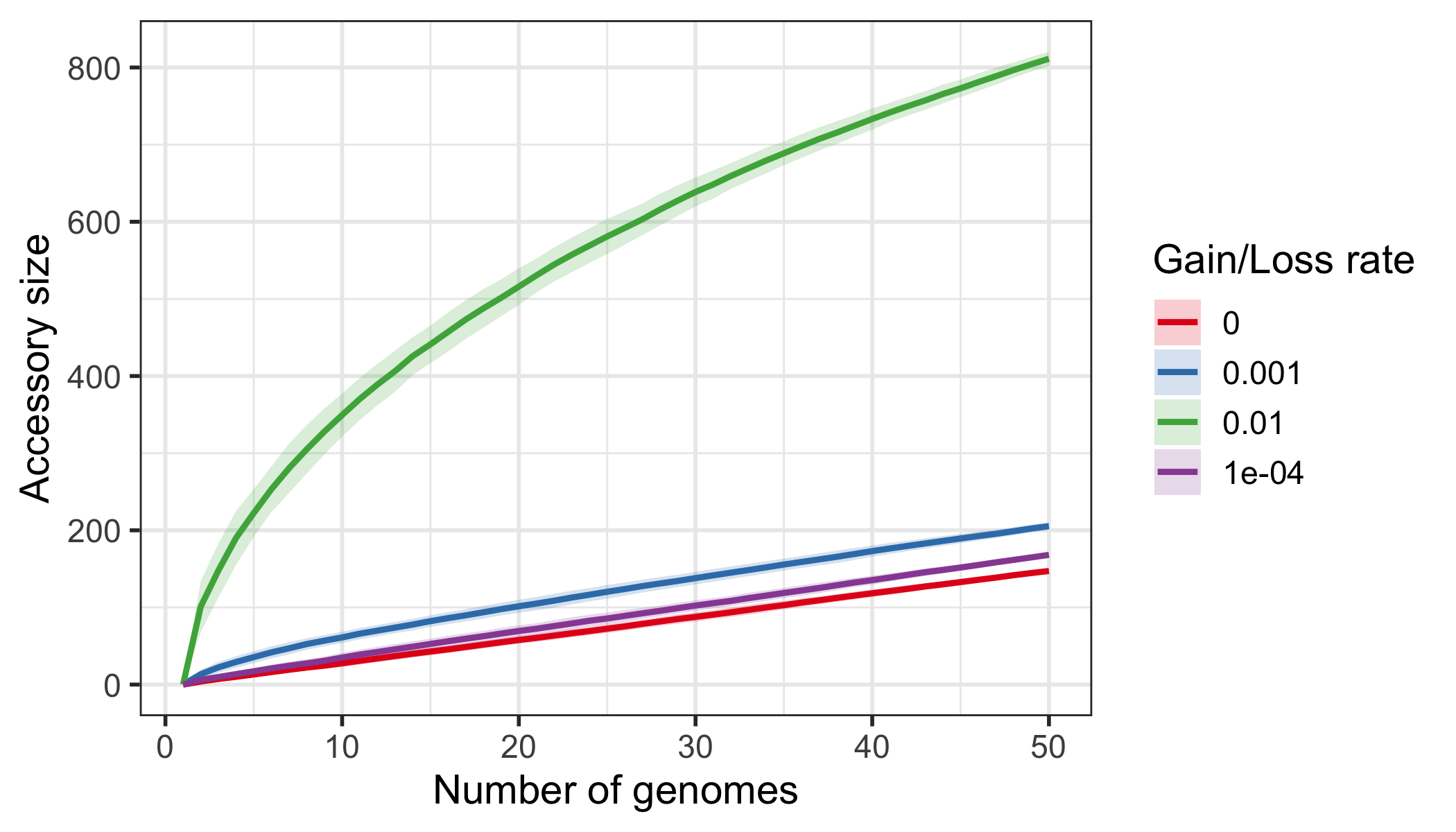

Supplement: Supplemental Material [file supp_gr.277340.122_Supplemental_Code_0.1.0.tar.gz.zip › panstripe-manuscript-0.1.0/figures/gain_loss_rate_acc.png]

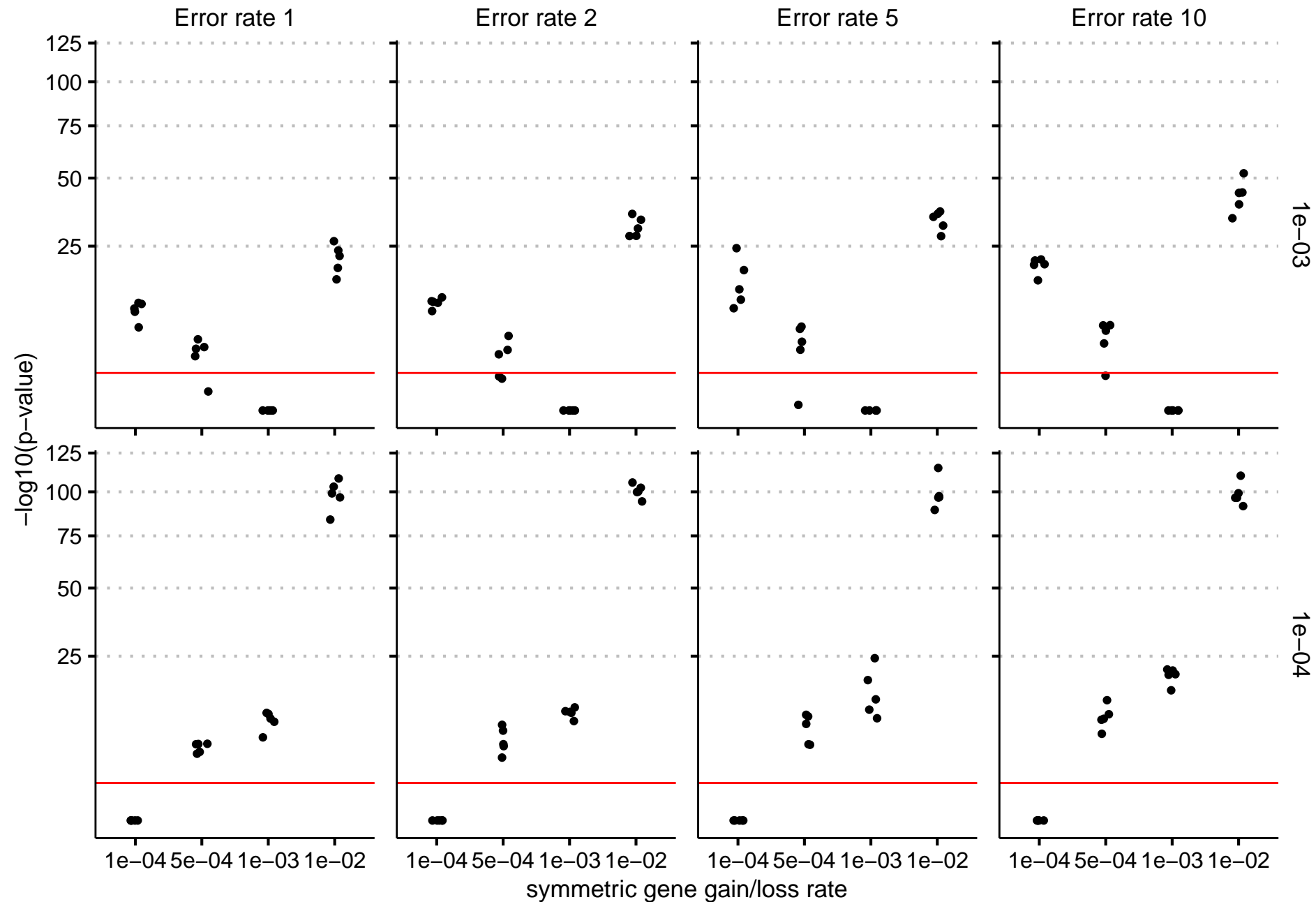

Supplement: Supplemental Material [file supp_gr.277340.122_Supplemental_Code_0.1.0.tar.gz.zip › panstripe-manuscript-0.1.0/figures/gl_rate_comparison.pdf]

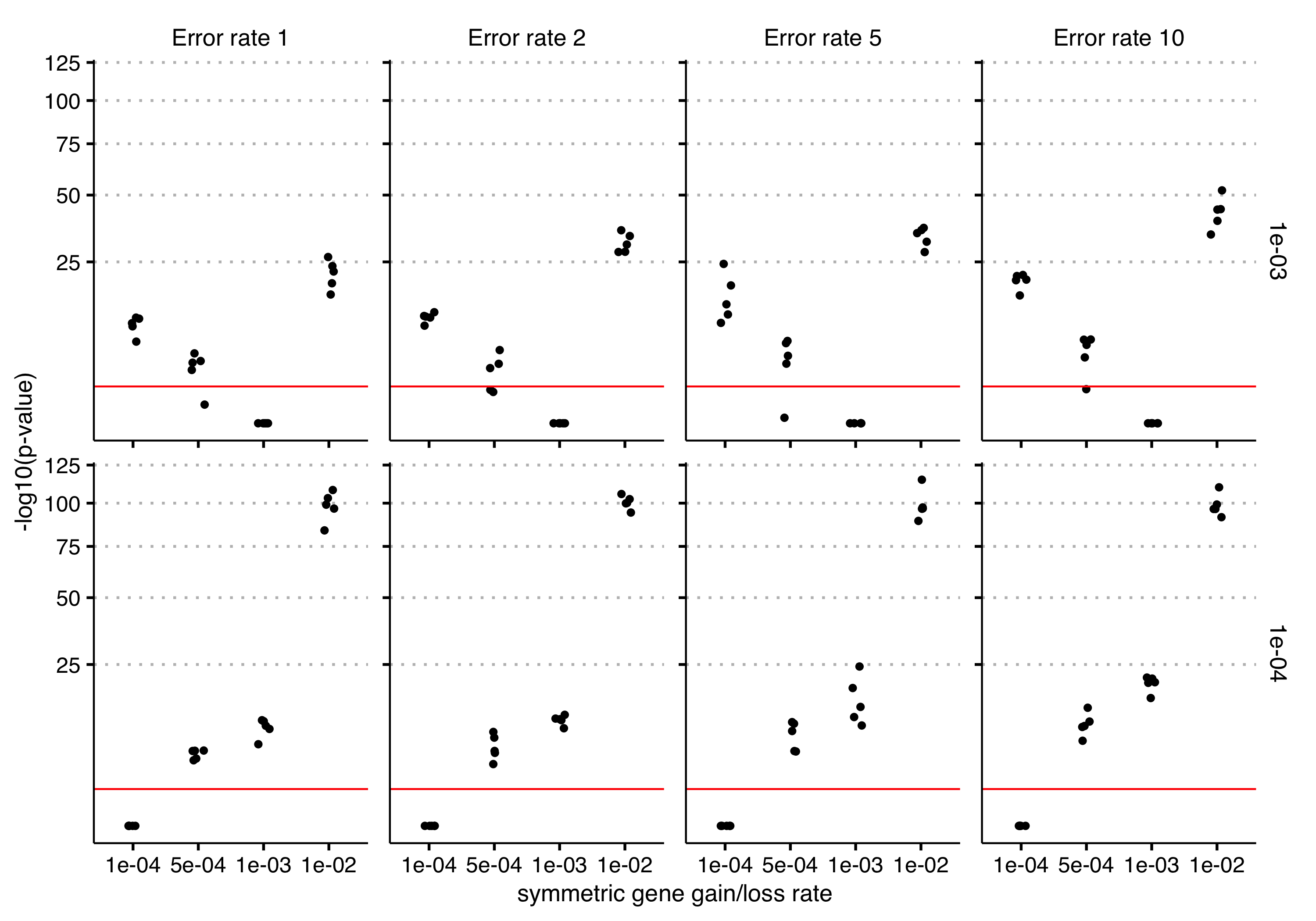

Supplement: Supplemental Material [file supp_gr.277340.122_Supplemental_Code_0.1.0.tar.gz.zip › panstripe-manuscript-0.1.0/figures/gl_rate_comparison.png]

Invasive

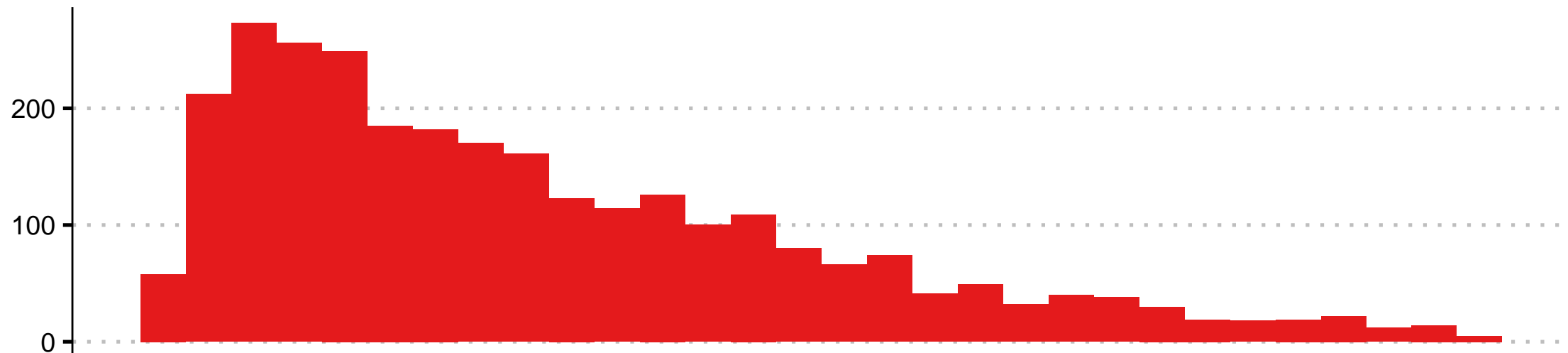

Not invasive

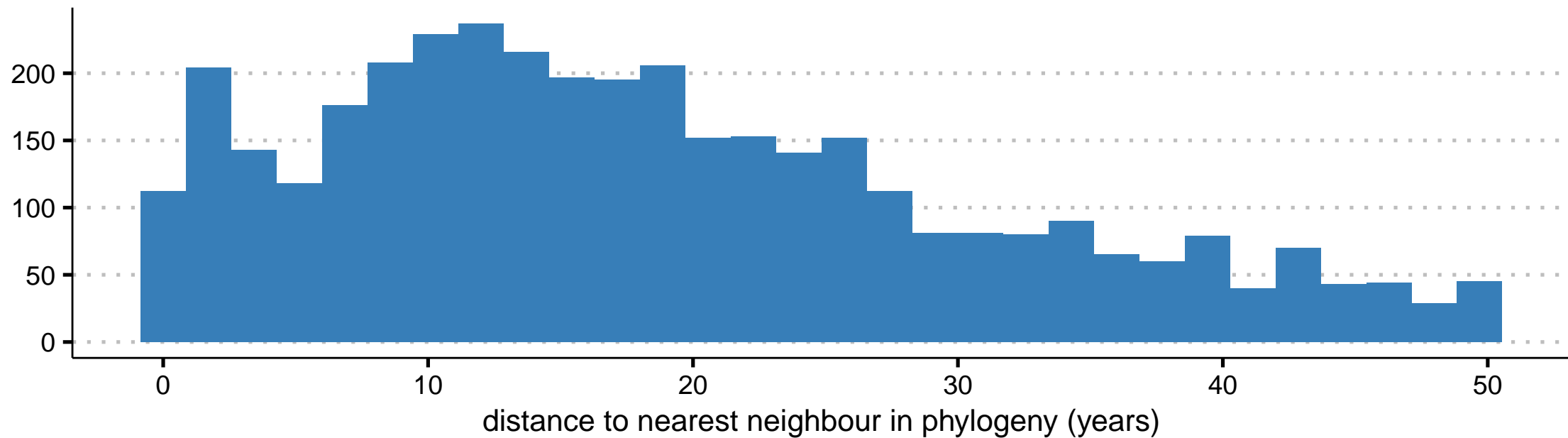

Supplement: Supplemental Material [file supp_gr.277340.122_Supplemental_Code_0.1.0.tar.gz.zip › panstripe-manuscript-0.1.0/figures/GPSC_nn_histogram.pdf]

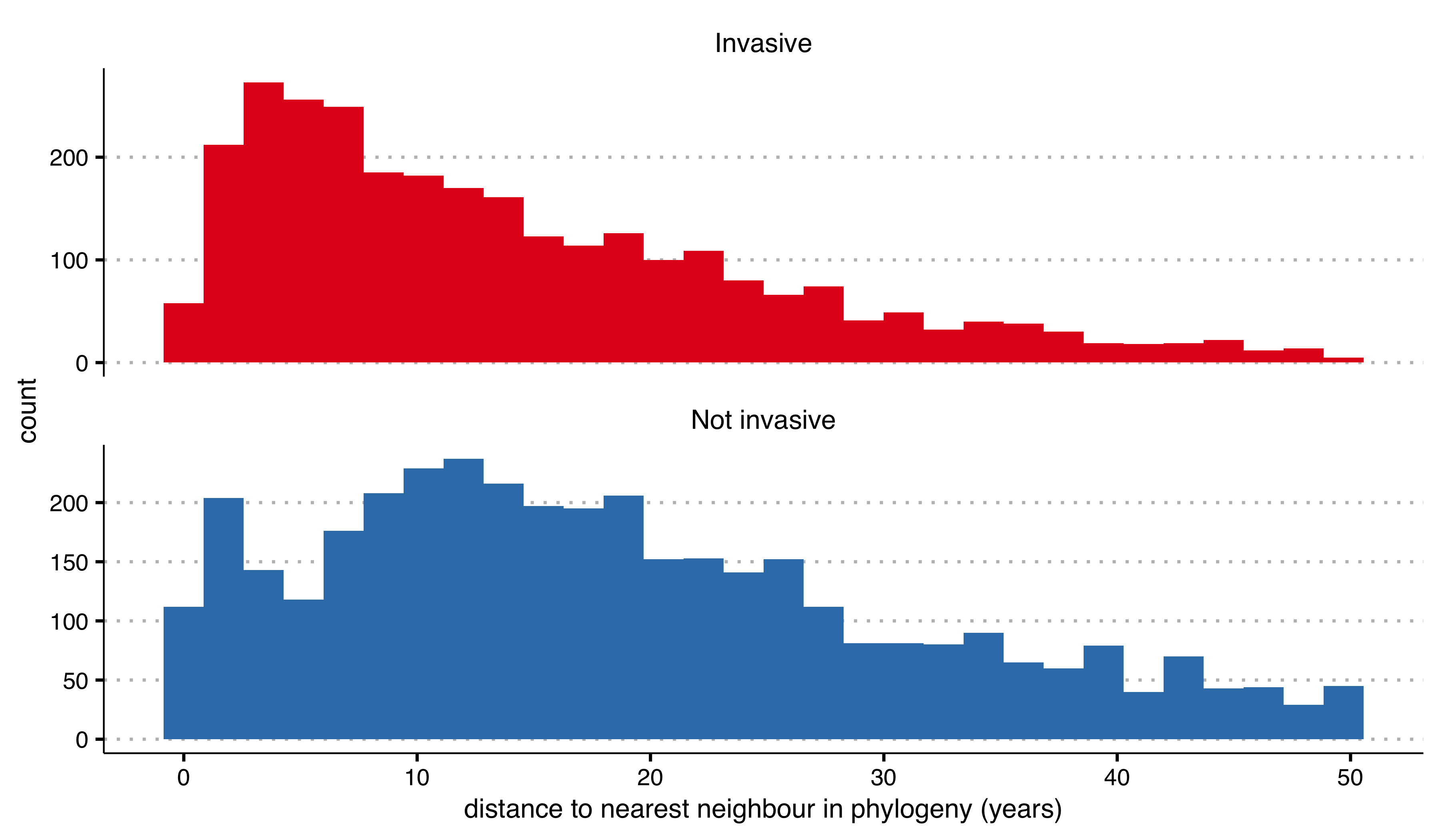

Supplement: Supplemental Material [file supp_gr.277340.122_Supplemental_Code_0.1.0.tar.gz.zip › panstripe-manuscript-0.1.0/figures/GPSC_nn_histogram.png]

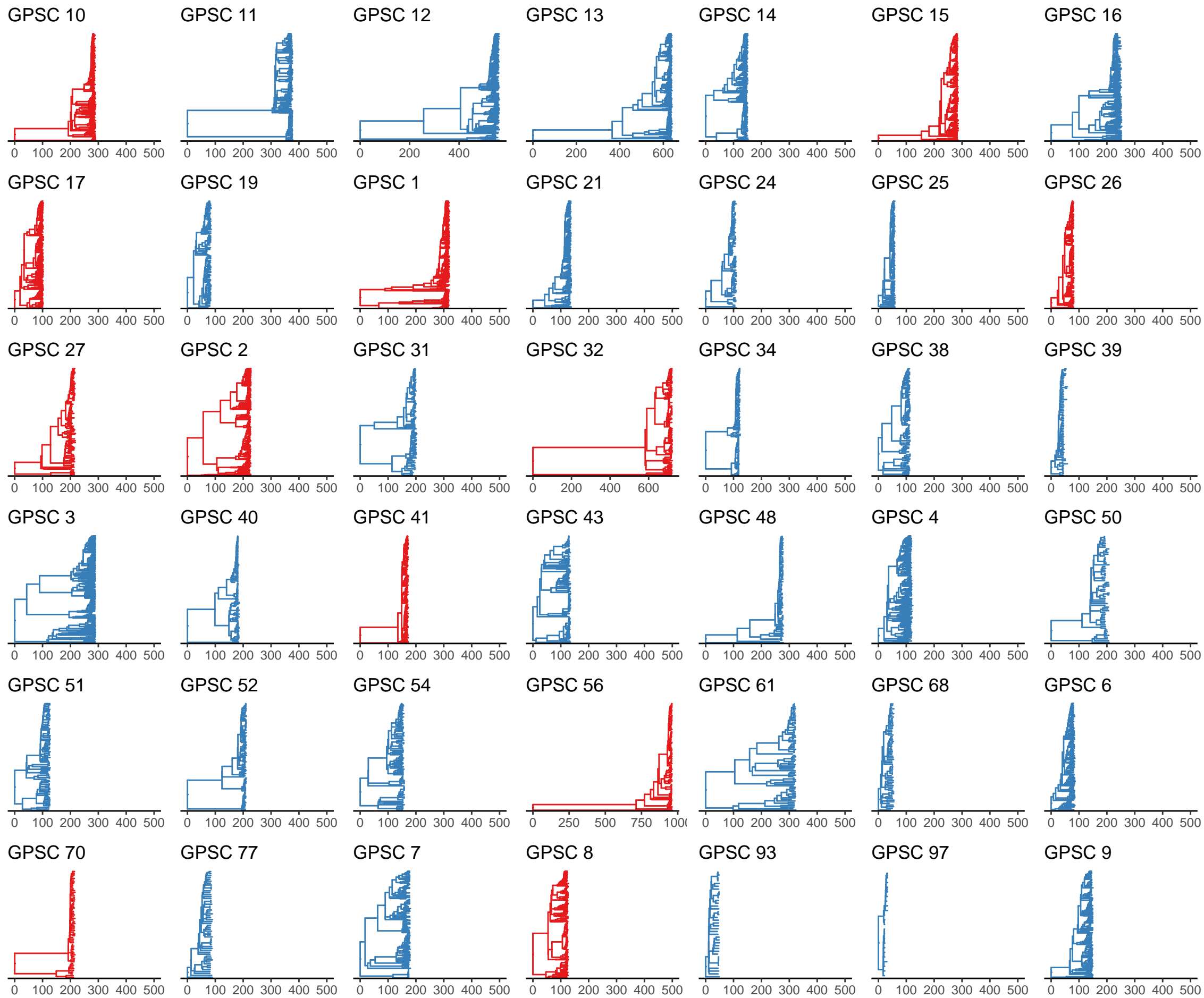

Supplement: Supplemental Material [file supp_gr.277340.122_Supplemental_Code_0.1.0.tar.gz.zip › panstripe-manuscript-0.1.0/figures/GPSC_phylogenies_sigOR.pdf]

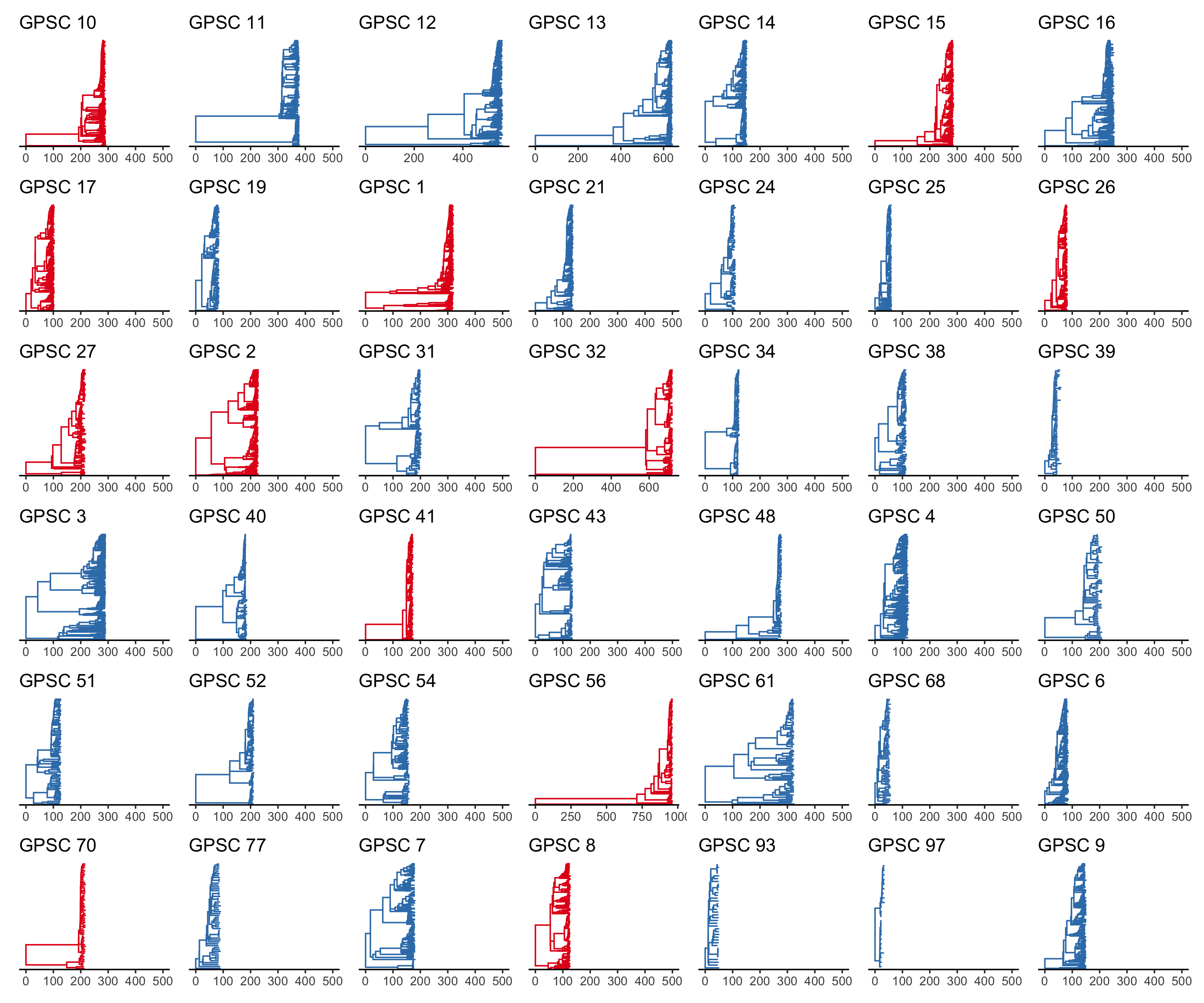

Supplement: Supplemental Material [file supp_gr.277340.122_Supplemental_Code_0.1.0.tar.gz.zip › panstripe-manuscript-0.1.0/figures/GPSC_phylogenies_sigOR.png]

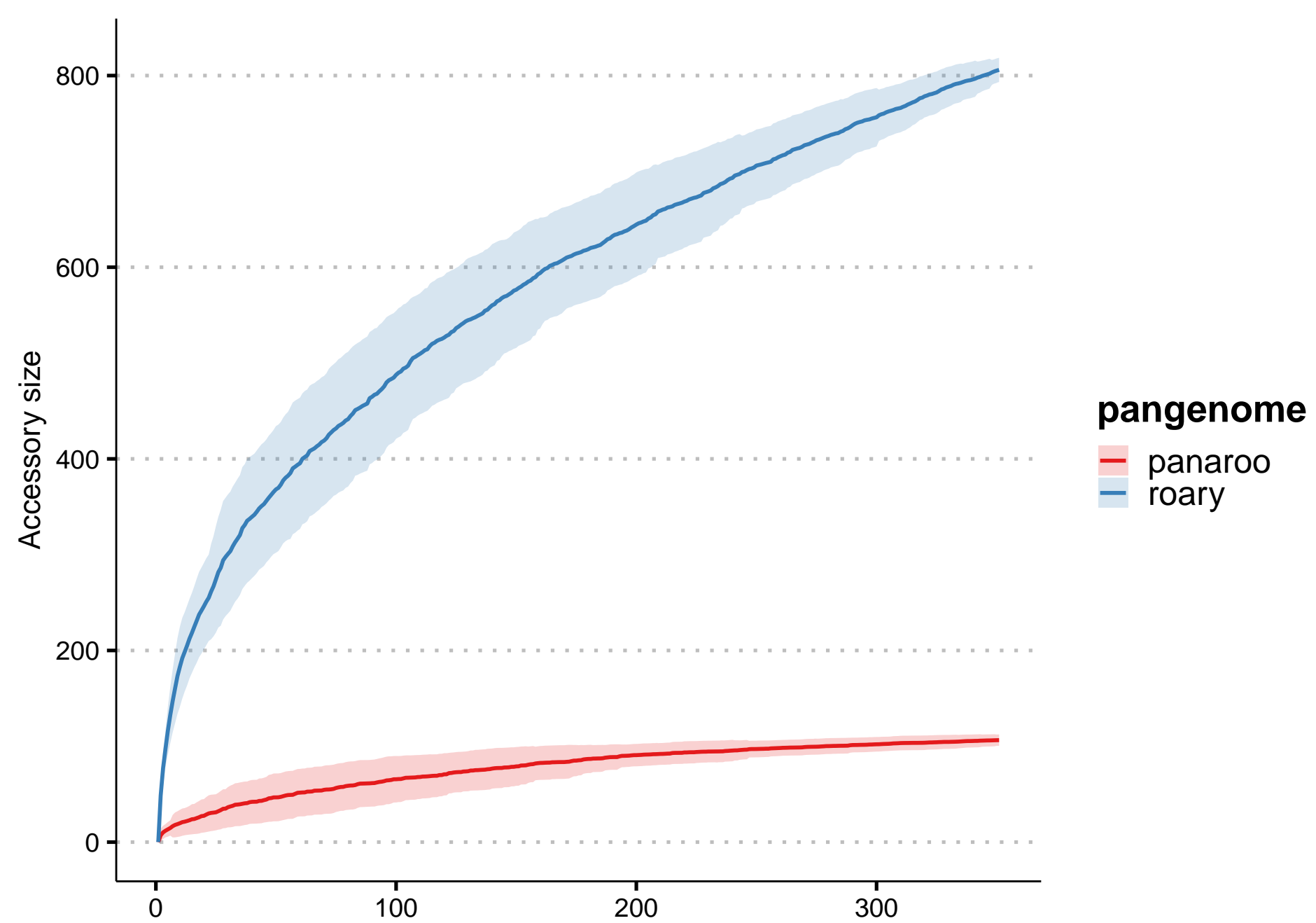

Supplement: Supplemental Material [file supp_gr.277340.122_Supplemental_Code_0.1.0.tar.gz.zip › panstripe-manuscript-0.1.0/figures/mtb_accumulation_curve_panaroo_roary.pdf]

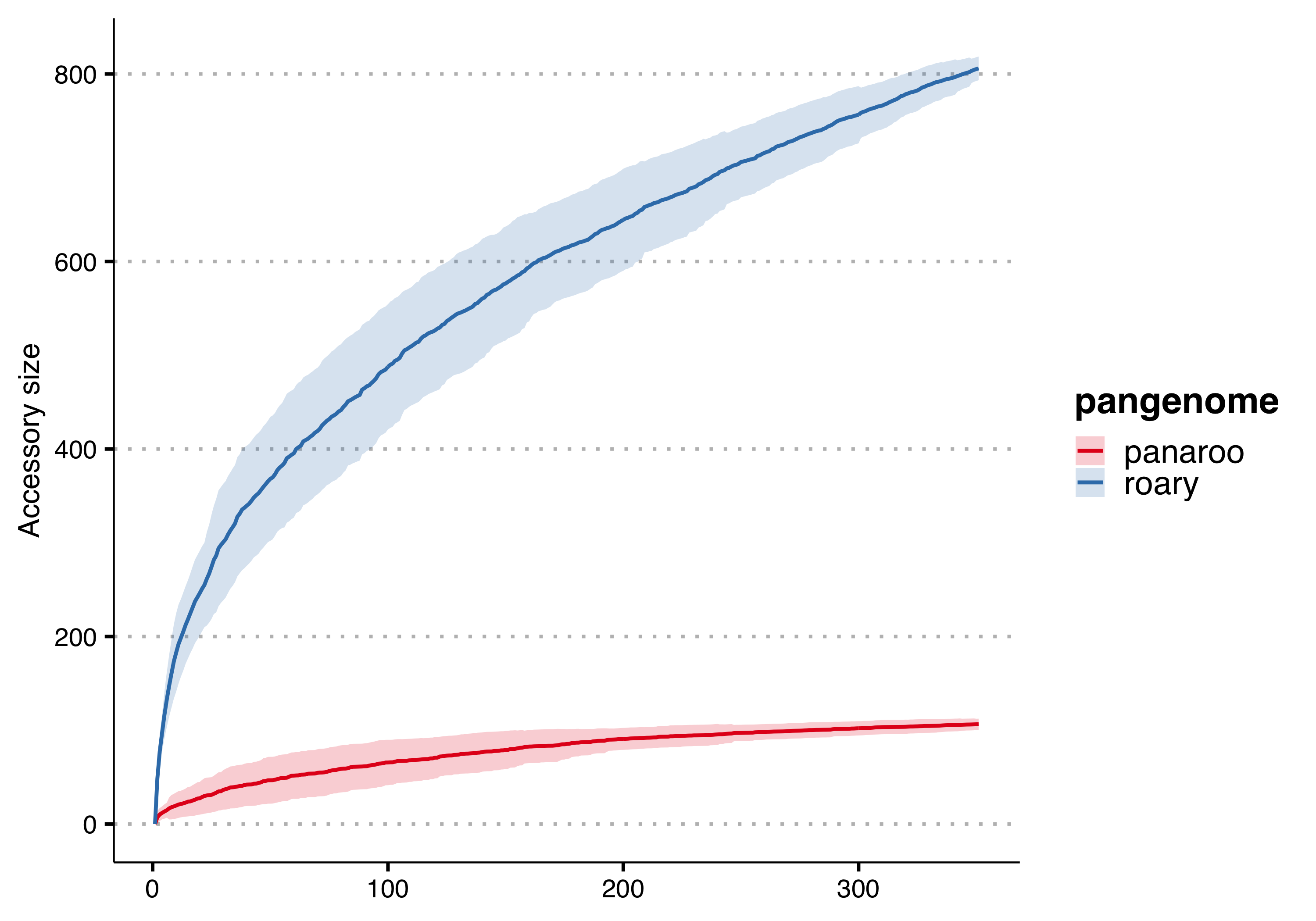

Supplement: Supplemental Material [file supp_gr.277340.122_Supplemental_Code_0.1.0.tar.gz.zip › panstripe-manuscript-0.1.0/figures/mtb_accumulation_curve_panaroo_roary.png]

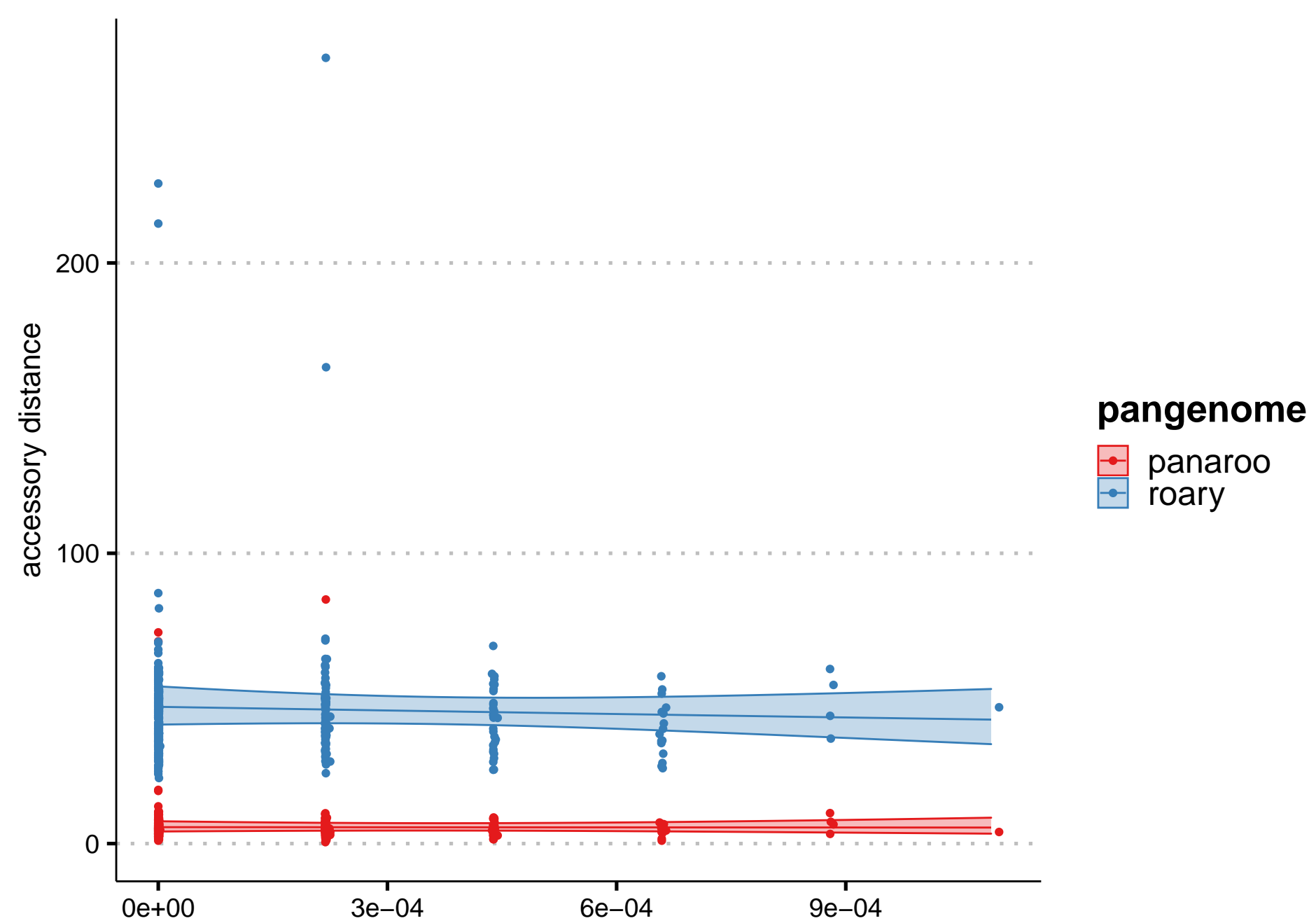

Supplement: Supplemental Material [file supp_gr.277340.122_Supplemental_Code_0.1.0.tar.gz.zip › panstripe-manuscript-0.1.0/figures/mtb_panstripe_panaroo_roary.pdf]

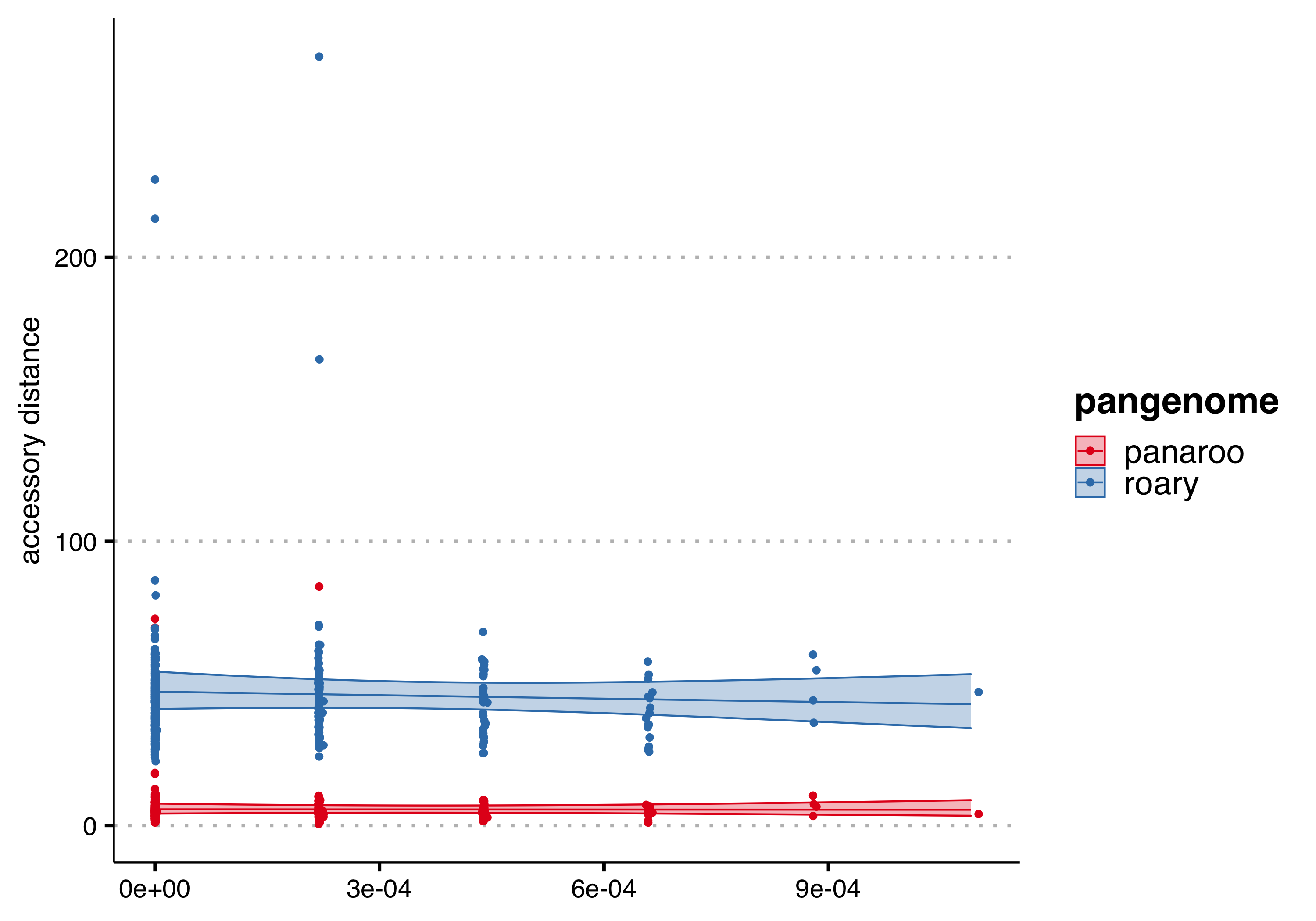

Supplement: Supplemental Material [file supp_gr.277340.122_Supplemental_Code_0.1.0.tar.gz.zip › panstripe-manuscript-0.1.0/figures/mtb_panstripe_panaroo_roary.png]

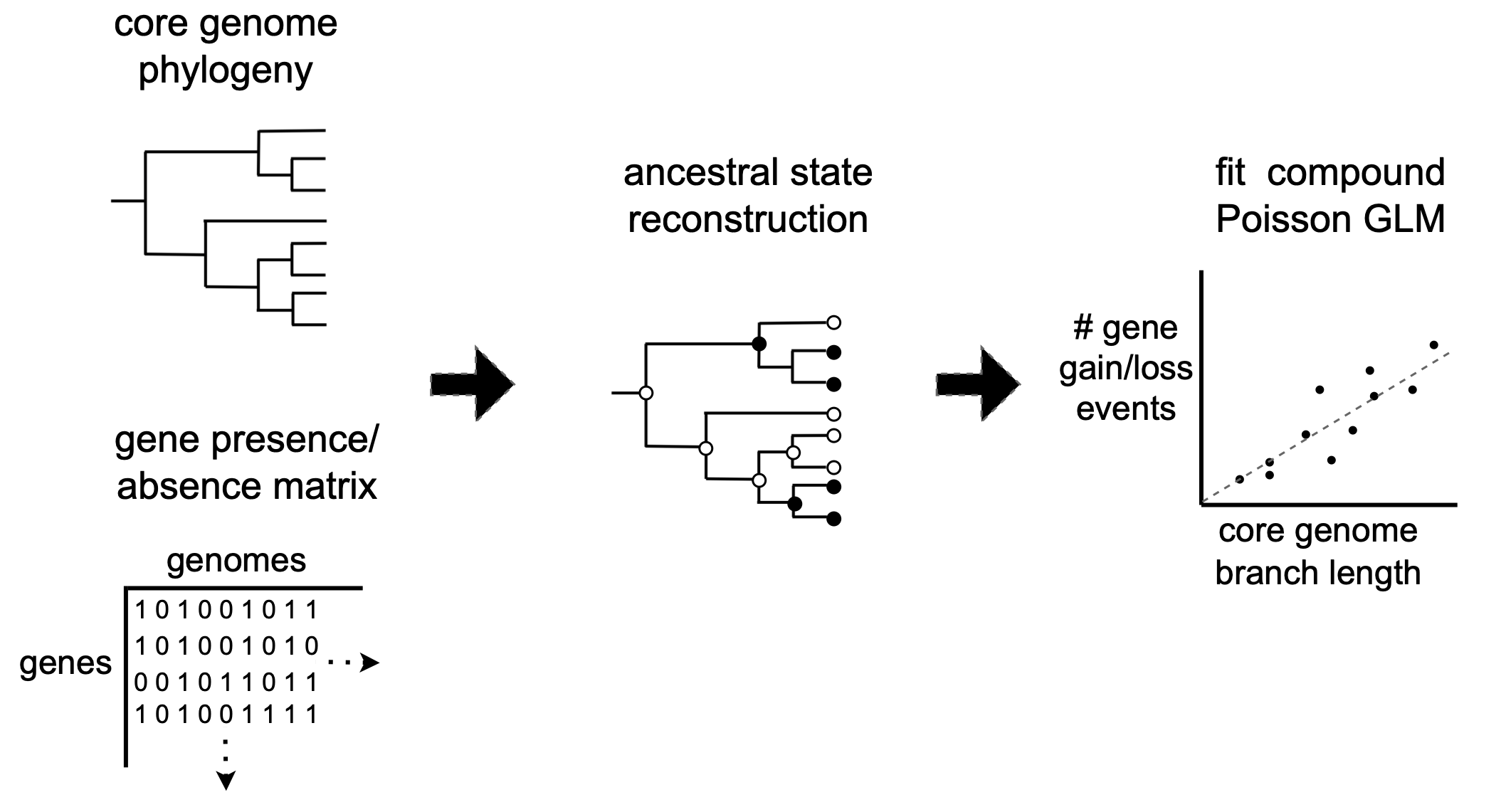

Supplement: Supplemental Material [file supp_gr.277340.122_Supplemental_Code_0.1.0.tar.gz.zip › panstripe-manuscript-0.1.0/figures/panstripe.drawio.png]

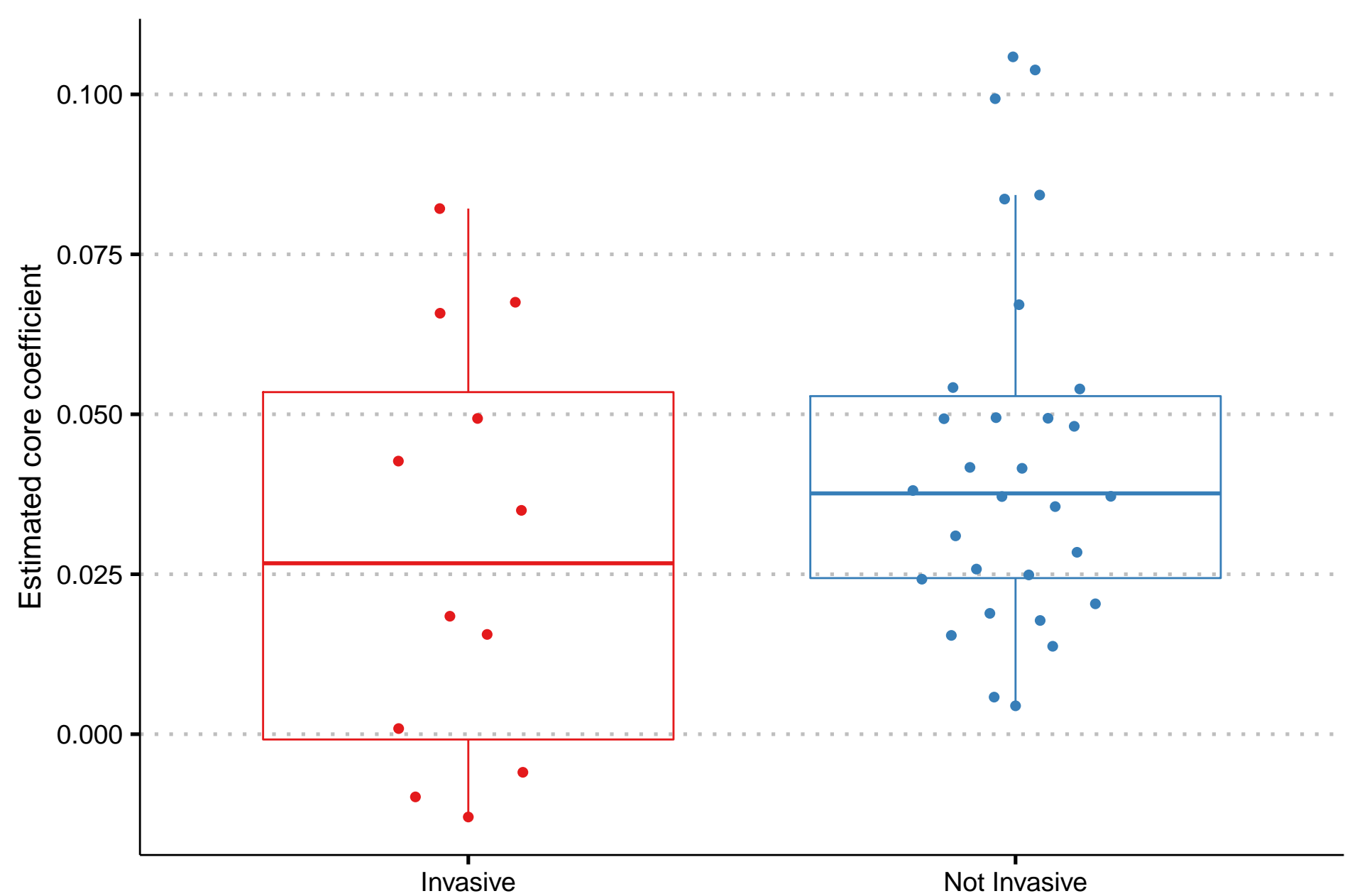

Supplement: Supplemental Material [file supp_gr.277340.122_Supplemental_Code_0.1.0.tar.gz.zip › panstripe-manuscript-0.1.0/figures/pneumo_core_estimate_boxplot.pdf]

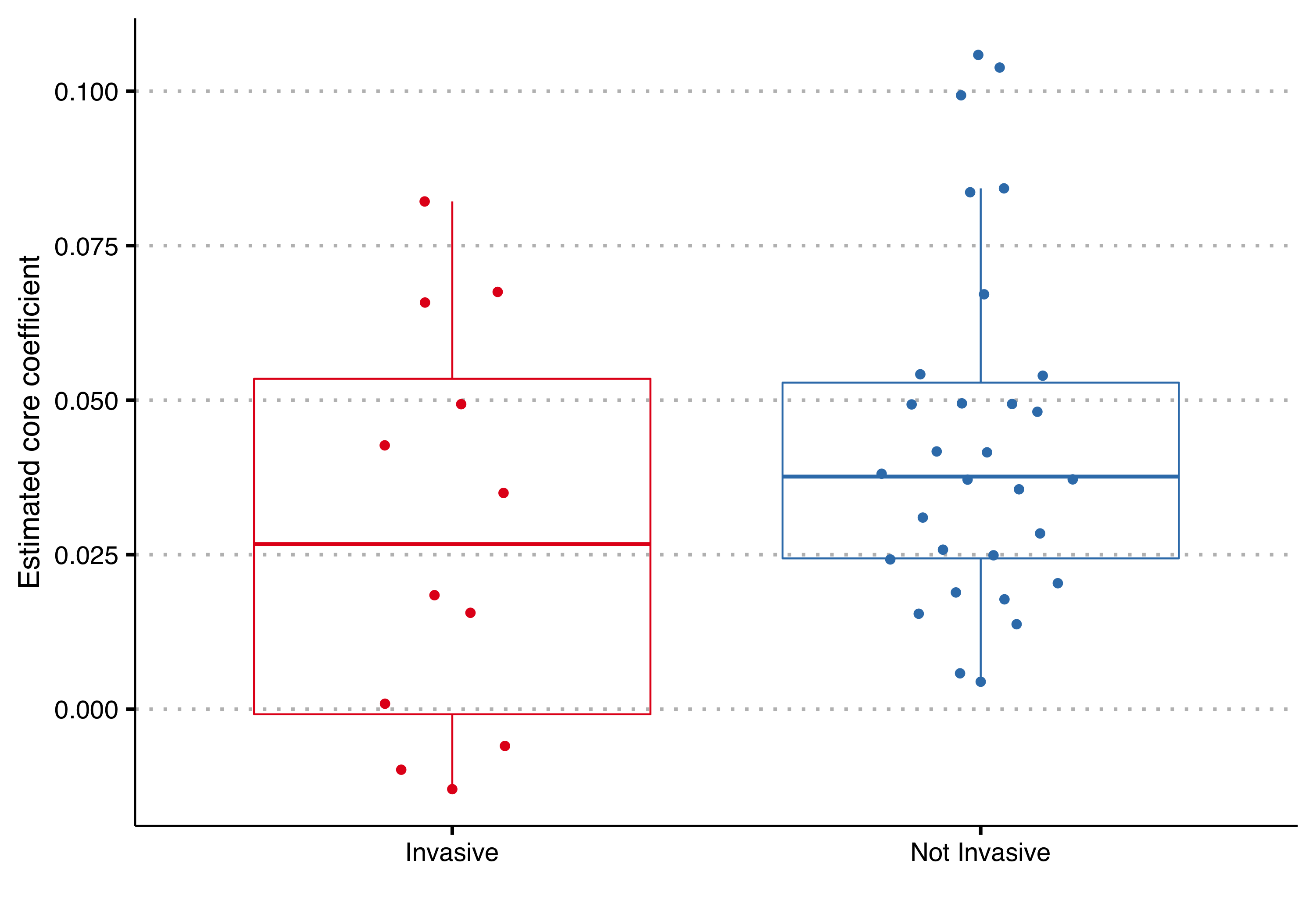

Supplement: Supplemental Material [file supp_gr.277340.122_Supplemental_Code_0.1.0.tar.gz.zip › panstripe-manuscript-0.1.0/figures/pneumo_core_estimate_boxplot.png]

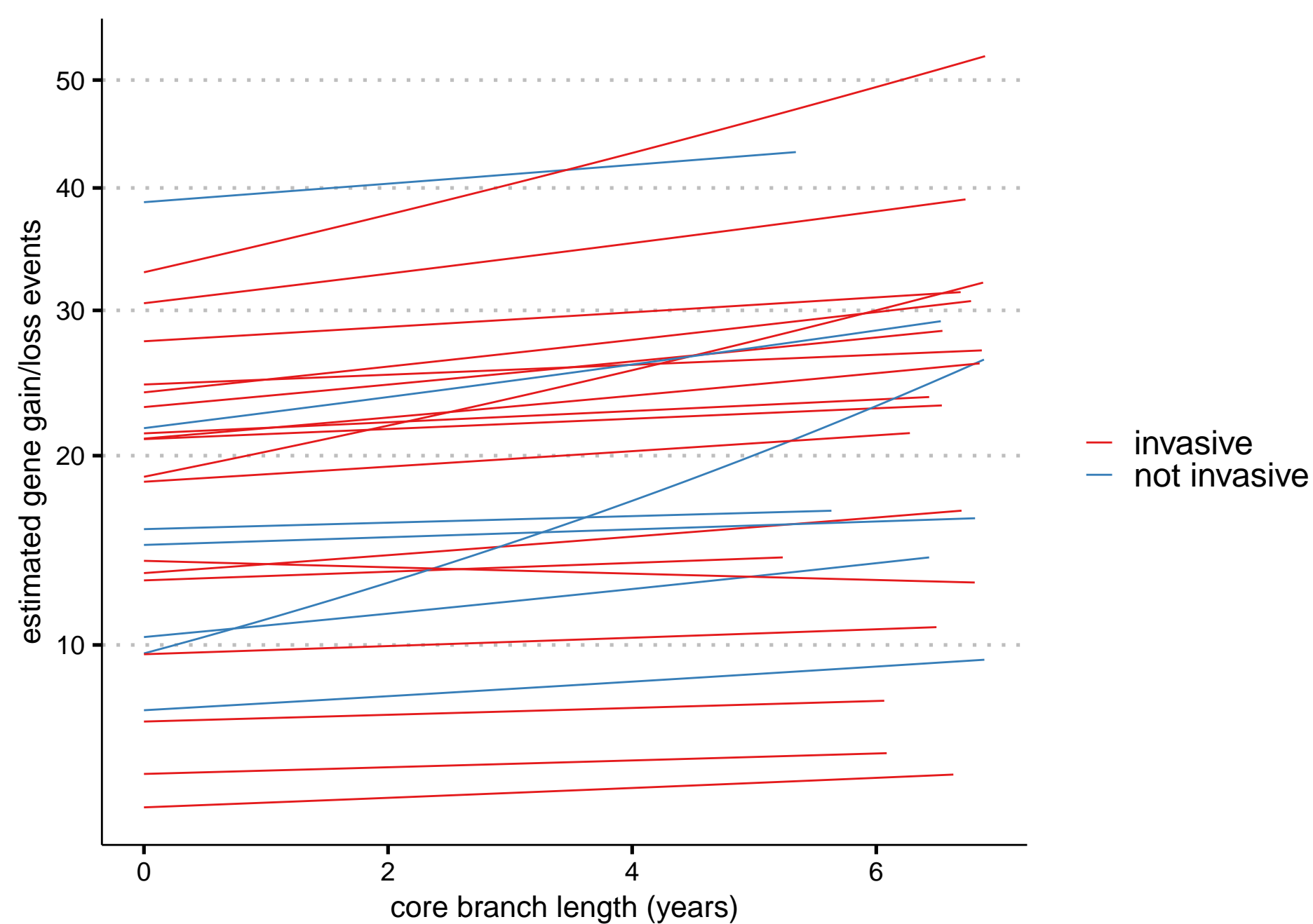

Supplement: Supplemental Material [file supp_gr.277340.122_Supplemental_Code_0.1.0.tar.gz.zip › panstripe-manuscript-0.1.0/figures/pneumo_panstripe_fits.pdf]

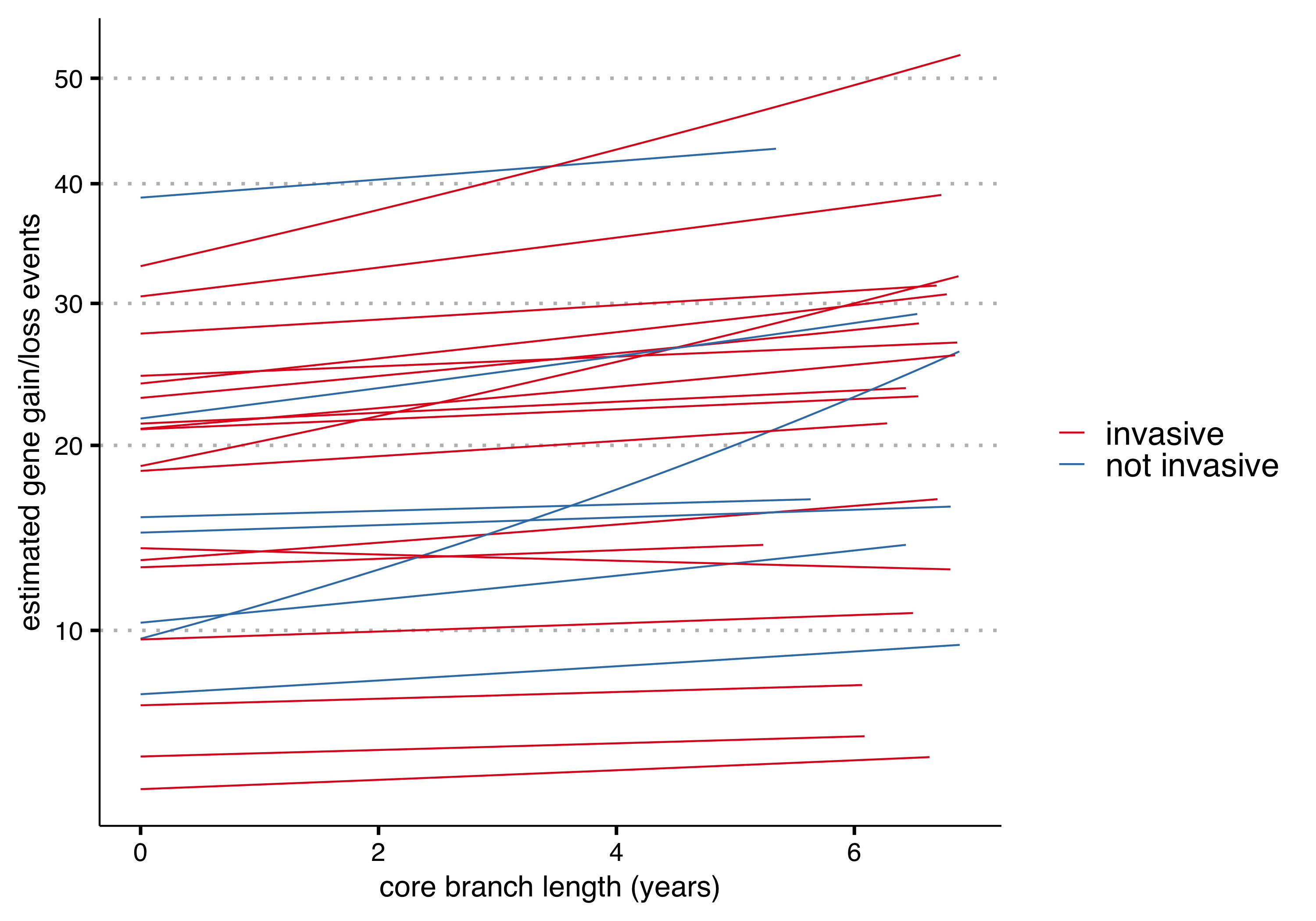

Supplement: Supplemental Material [file supp_gr.277340.122_Supplemental_Code_0.1.0.tar.gz.zip › panstripe-manuscript-0.1.0/figures/pneumo_panstripe_fits.png]

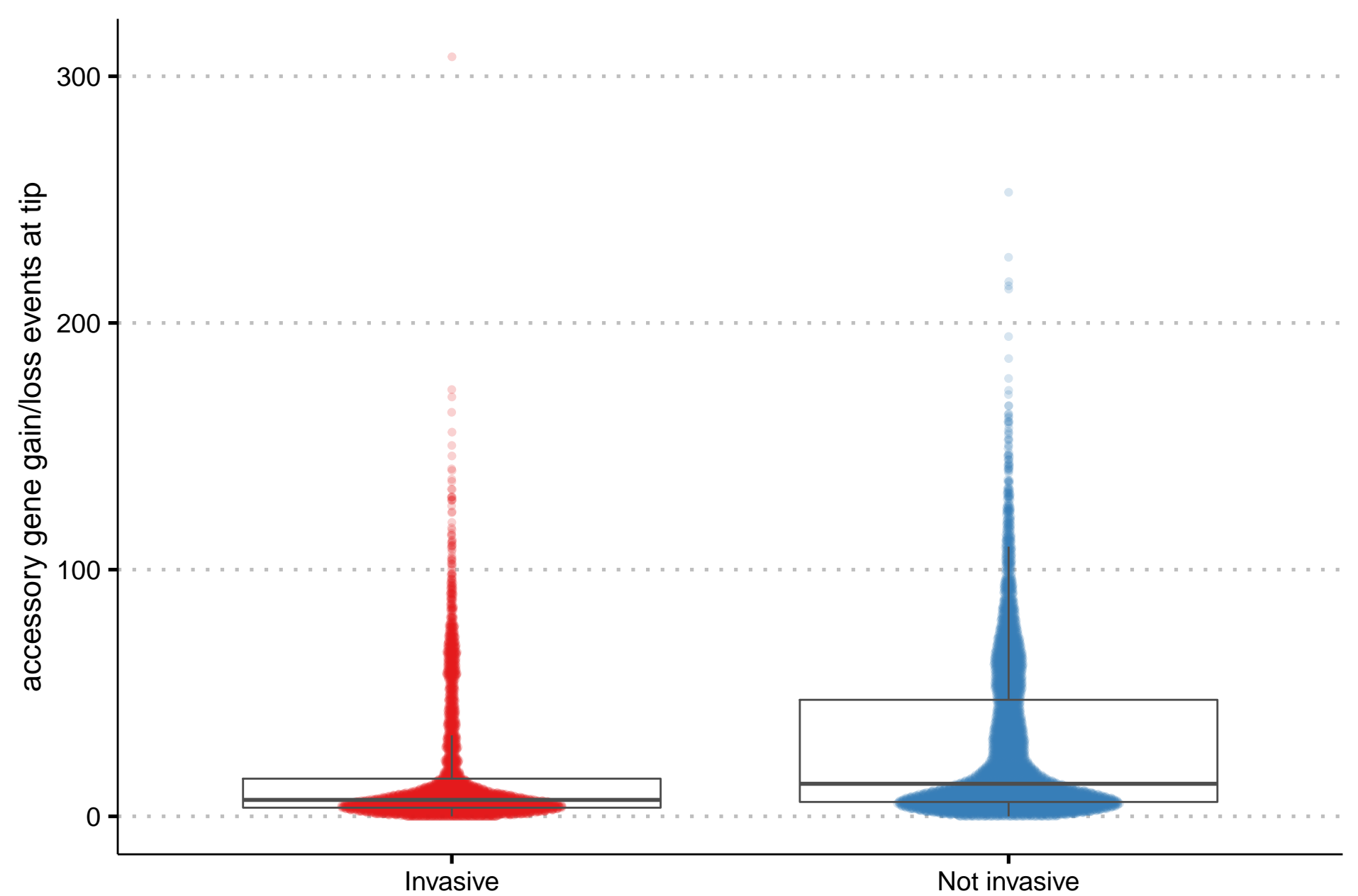

Supplement: Supplemental Material [file supp_gr.277340.122_Supplemental_Code_0.1.0.tar.gz.zip › panstripe-manuscript-0.1.0/figures/pneumo_tip_acc_count_histogram.pdf]

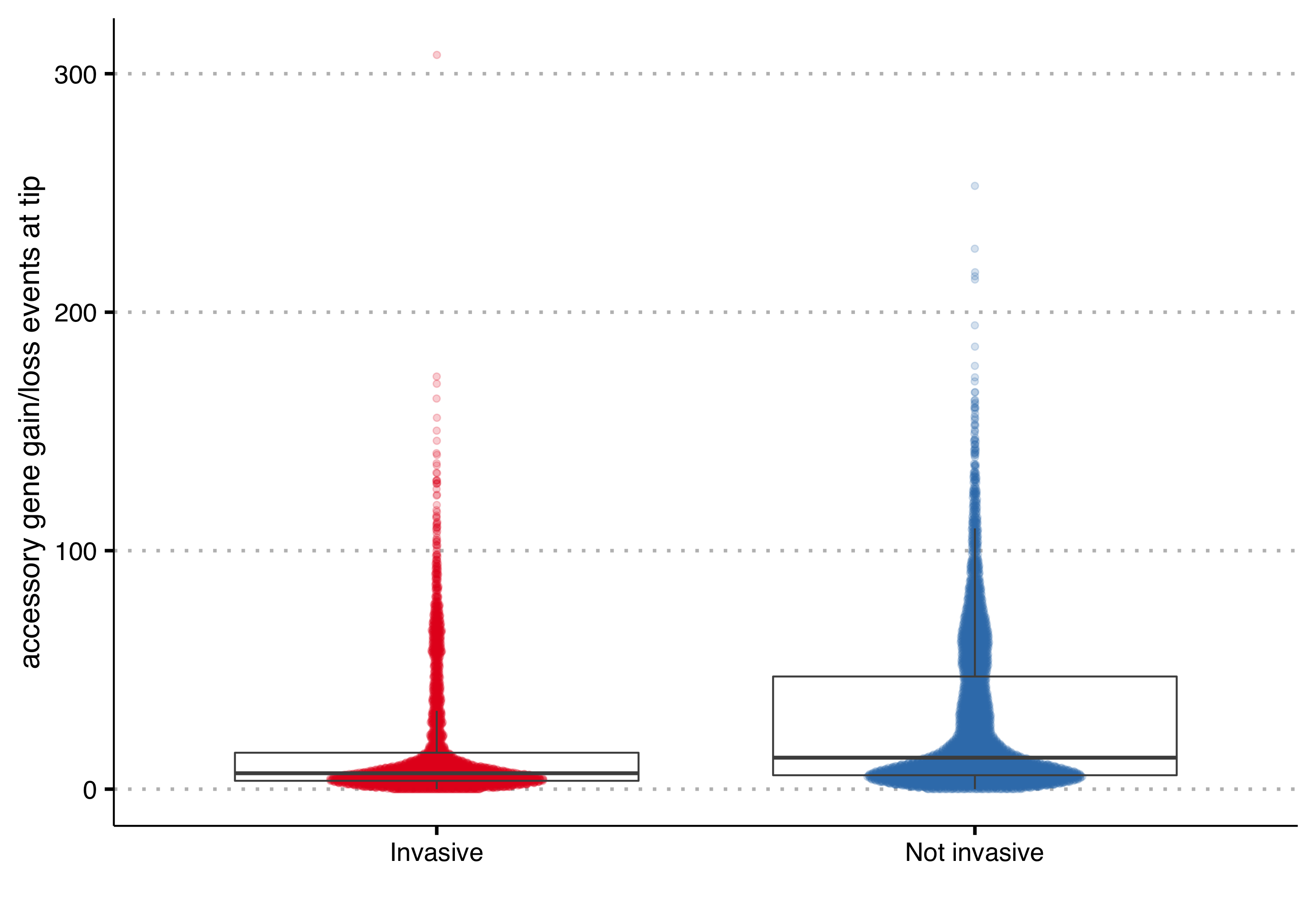

Supplement: Supplemental Material [file supp_gr.277340.122_Supplemental_Code_0.1.0.tar.gz.zip › panstripe-manuscript-0.1.0/figures/pneumo_tip_acc_count_histogram.png]

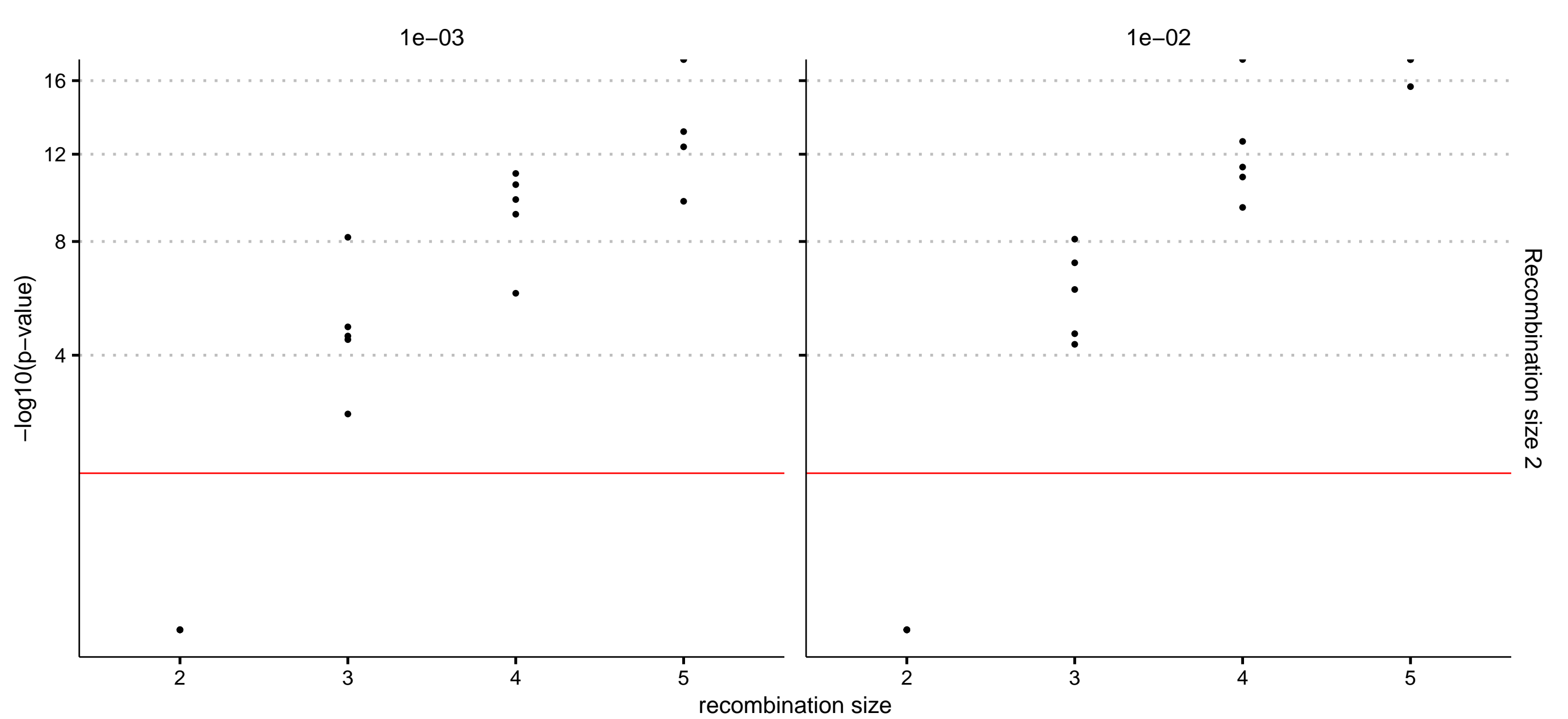

Supplement: Supplemental Material [file supp_gr.277340.122_Supplemental_Code_0.1.0.tar.gz.zip › panstripe-manuscript-0.1.0/figures/recombination_size_comparison.pdf]

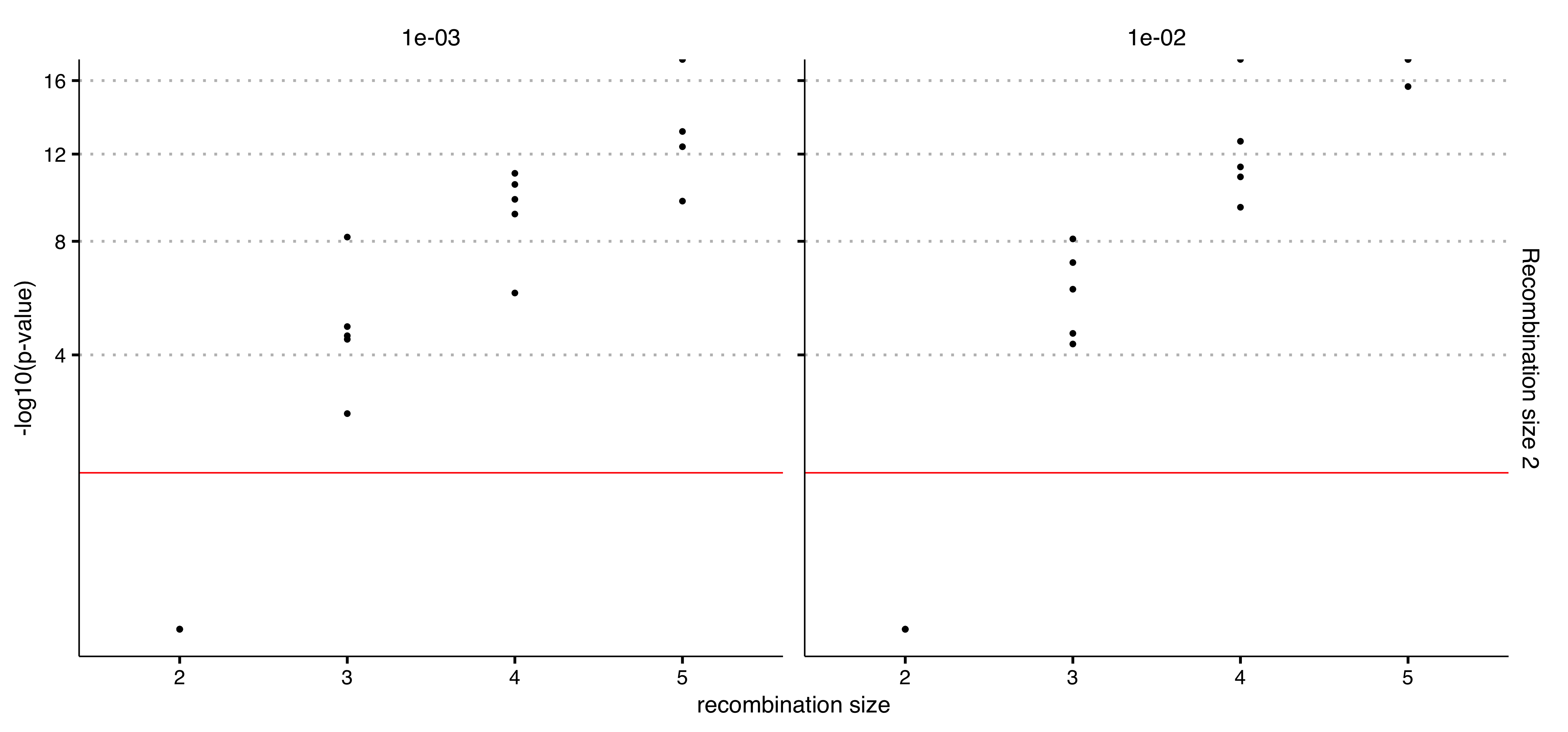

Supplement: Supplemental Material [file supp_gr.277340.122_Supplemental_Code_0.1.0.tar.gz.zip › panstripe-manuscript-0.1.0/figures/recombination_size_comparison.png]

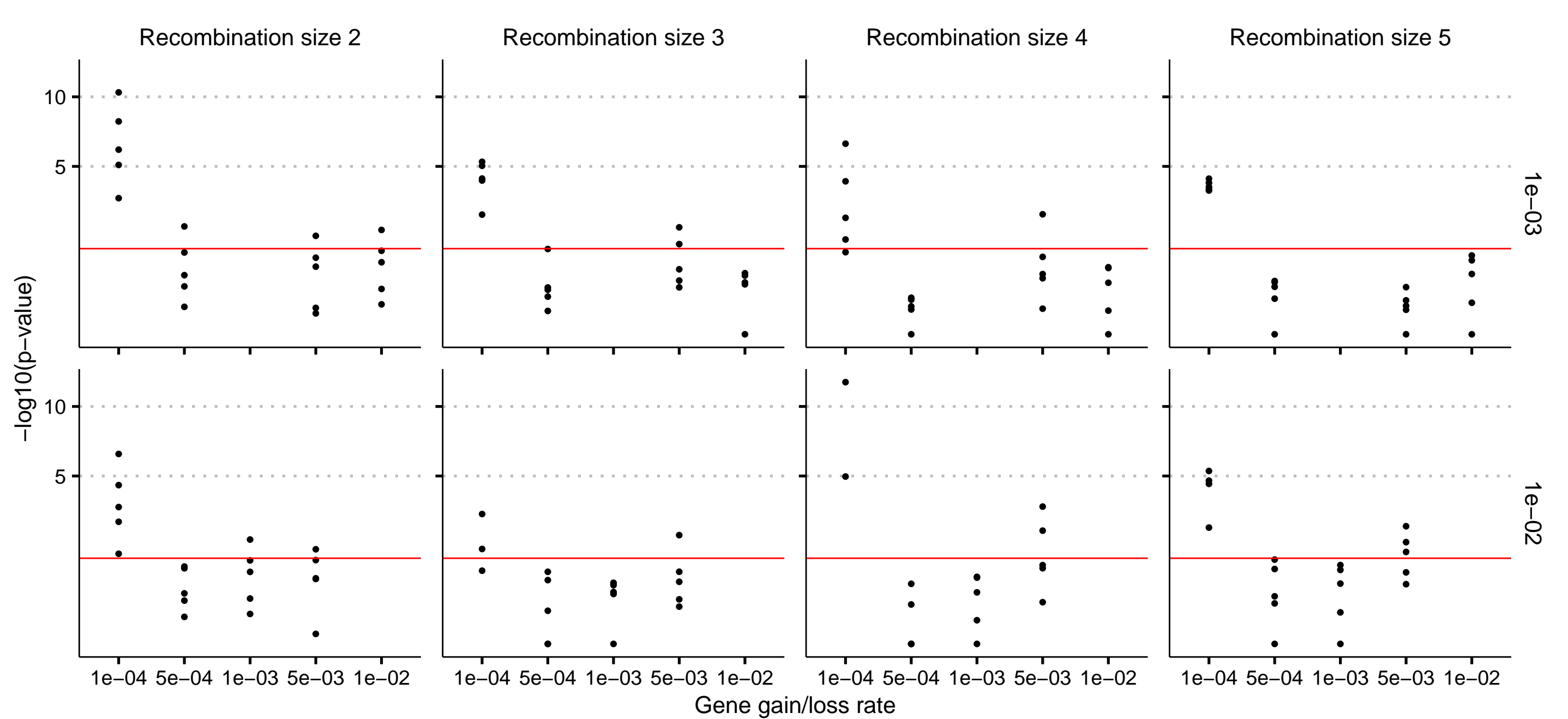

Supplement: Supplemental Material [file supp_gr.277340.122_Supplemental_Code_0.1.0.tar.gz.zip › panstripe-manuscript-0.1.0/figures/recombination_size_comparison_nochange.pdf]

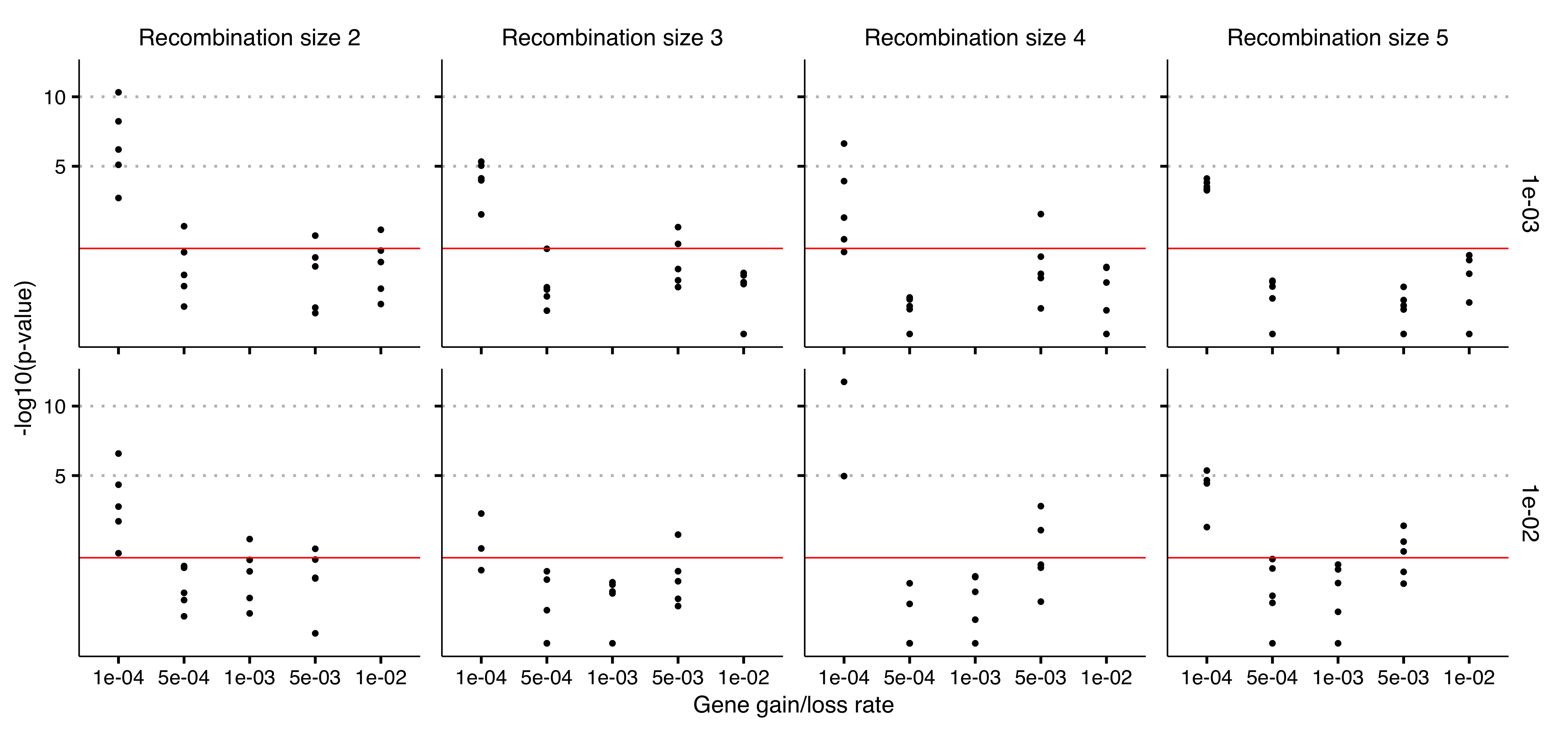

Supplement: Supplemental Material [file supp_gr.277340.122_Supplemental_Code_0.1.0.tar.gz.zip › panstripe-manuscript-0.1.0/figures/recombination_size_comparison_nochange.png]

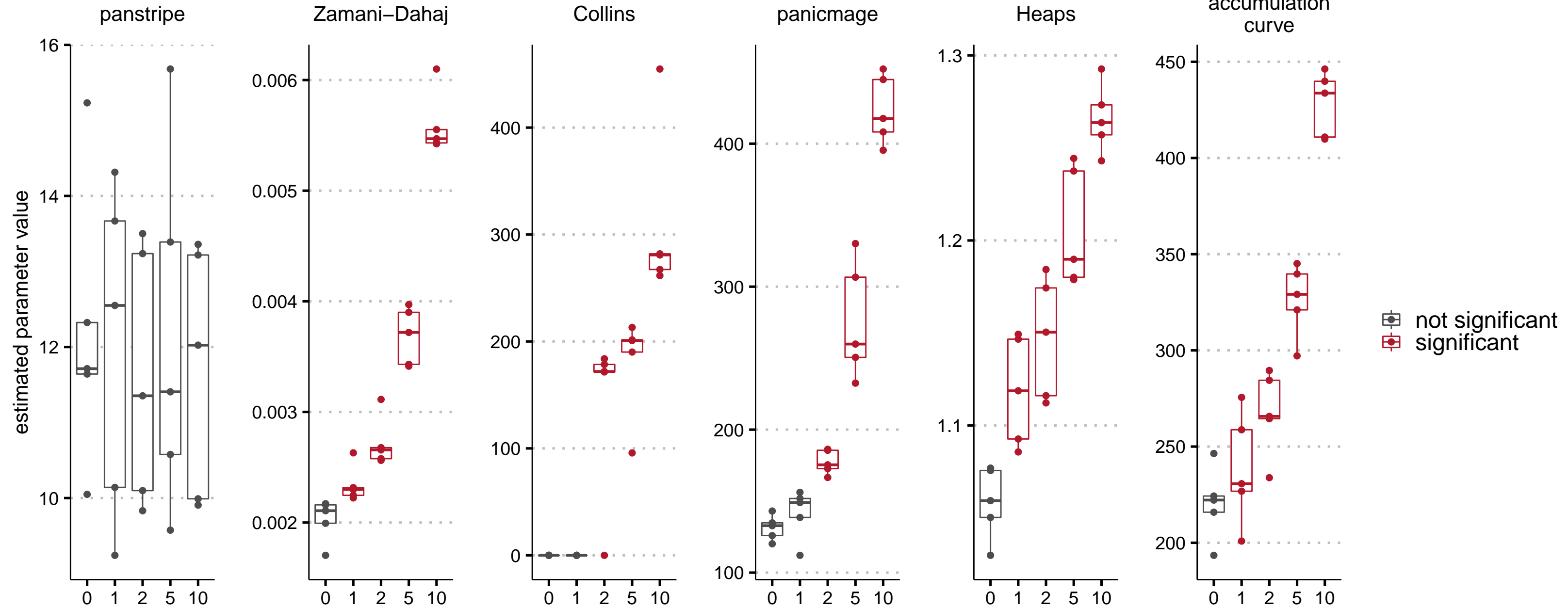

Supplement: Supplemental Material [file supp_gr.277340.122_Supplemental_Code_0.1.0.tar.gz.zip › panstripe-manuscript-0.1.0/figures/simulation_error_rate_summary.pdf]

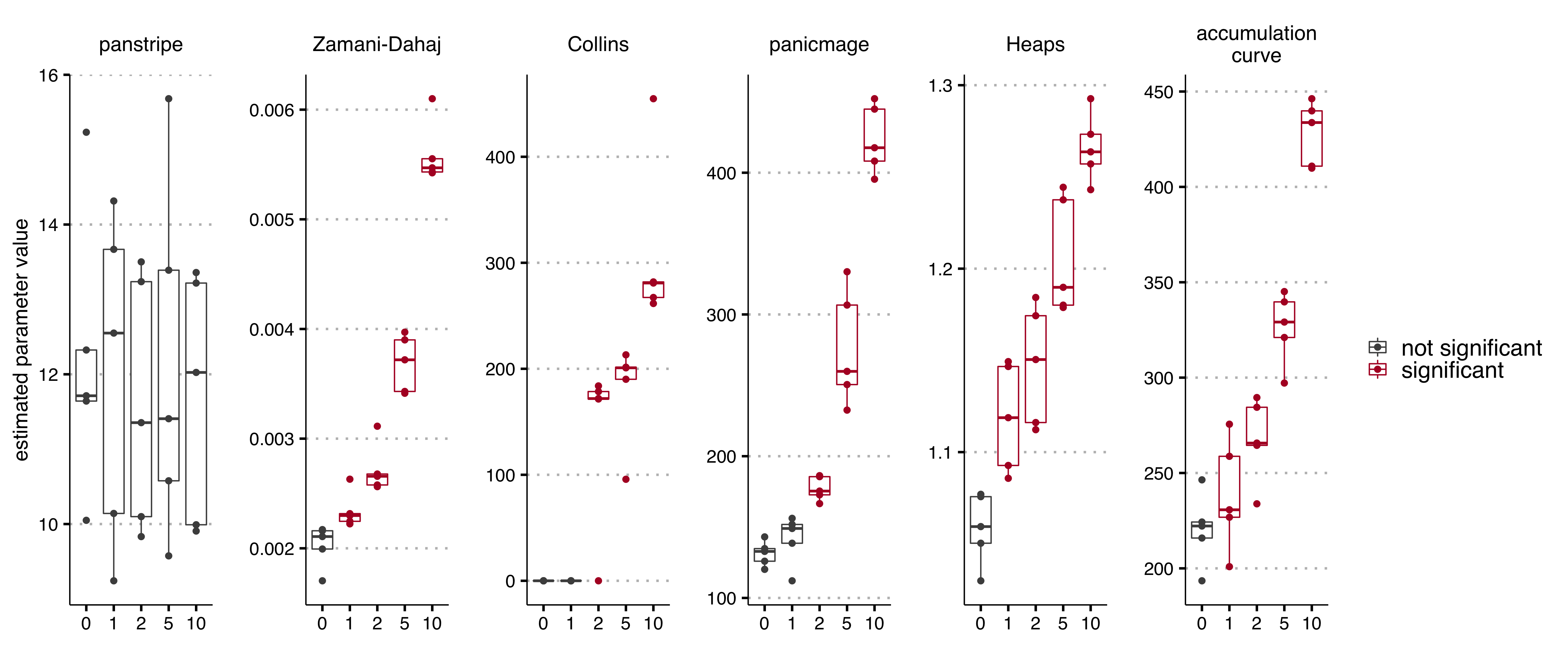

Supplement: Supplemental Material [file supp_gr.277340.122_Supplemental_Code_0.1.0.tar.gz.zip › panstripe-manuscript-0.1.0/figures/simulation_error_rate_summary.png]

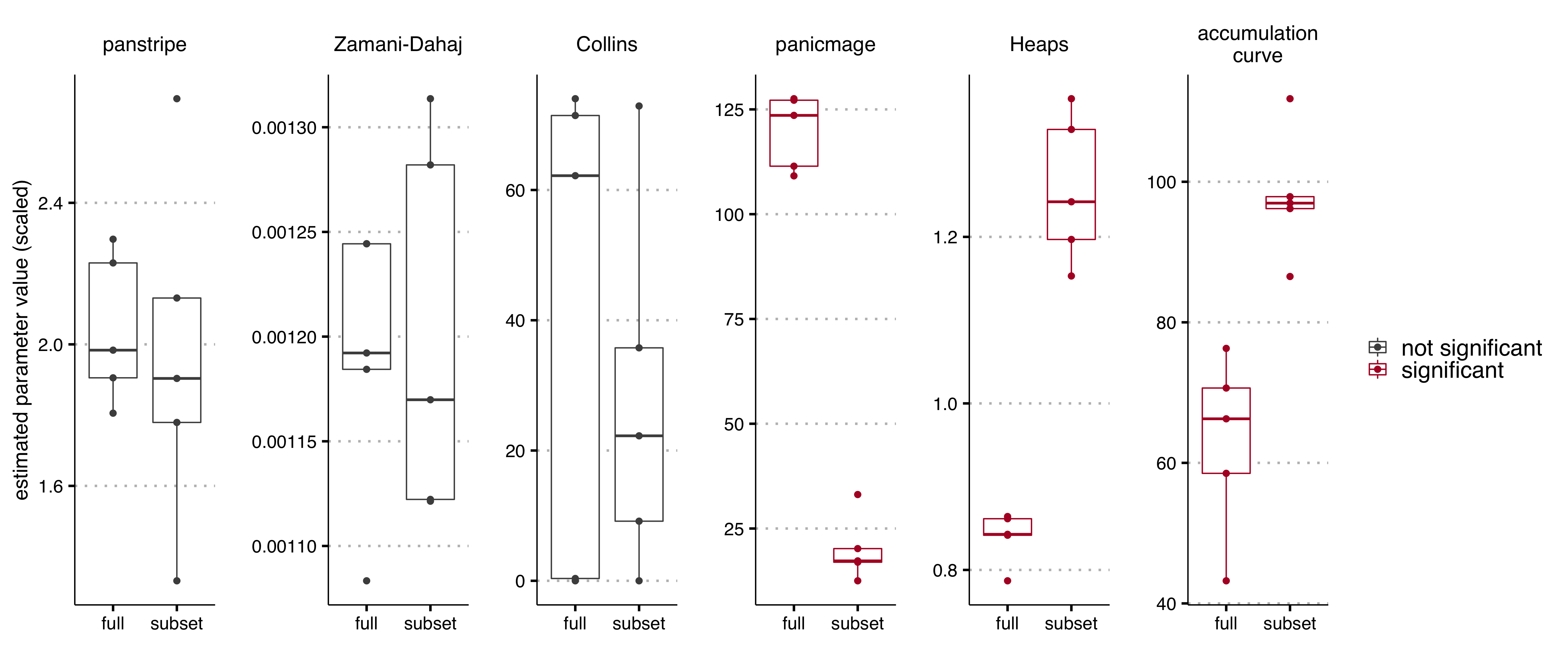

Supplement: Supplemental Material [file supp_gr.277340.122_Supplemental_Code_0.1.0.tar.gz.zip › panstripe-manuscript-0.1.0/figures/simulation_sampling_bias_summary.png]
